# L-HetNetAligner: A novel algorithm for Local Alignment of Heterogeneous Biological Networks

**DOI:** 10.1038/s41598-020-60737-5

**Published:** 2020-03-03

**Authors:** Marianna Milano, Tijana Milenković, Mario Cannataro, Pietro Hiram Guzzi

**Affiliations:** 1 Department of Surgical and Medical Sciences, University of Catanzaro, Catanzaro, 88040 Italy; 2 Department of Computer Science and Engineering, University of Notre Dame, Notre Dame, Indiana USA; 3 Data Analytics Research Center, University of Catanzaro, Catanzaro, Italy

**Keywords:** Data mining, Gene regulatory networks

## Abstract

Networks are largely used for modelling and analysing a wide range of biological data. As a consequence, many different research efforts have resulted in the introduction of a large number of algorithms for analysis and comparison of networks. Many of these algorithms can deal with networks with a single class of nodes and edges, also referred to as homogeneous networks. Recently, many different approaches tried to integrate into a single model the interplay of different molecules. A possible formalism to model such a scenario comes from node/edge coloured networks (also known as heterogeneous networks) implemented as node/ edge-coloured graphs. Therefore, the need for the introduction of algorithms able to compare heterogeneous networks arises. We here focus on the local comparison of heterogeneous networks, and we formulate it as a network alignment problem. To the best of our knowledge, the local alignment of heterogeneous networks has not been explored in the past. We here propose L-HetNetAligner a novel algorithm that receives as input two heterogeneous networks (node-coloured graphs) and builds a local alignment of them. We also implemented and tested our algorithm. Our results confirm that our method builds high-quality alignments. The following website *contains Supplementary File 1 material and the code.

## Introduction

Graph theory and its related formalisms^1,2^ may model many biological data and entities to help the elucidation of biological mechanisms. In such a scenario, biological entities are modelled using nodes of a graph, whose edges represent the associations among entities^3^. For instance, in computational biology, networks have been used to model interactions among biological macromolecules inside cells, such as protein-protein interactions (PPI), or gene-gene interactions^4,5^. Main characteristics of existing approaches are the modelling of a set of entities using a single node type (e.g., proteins or genes) and simple edge types^6^.

**Figure 1:**
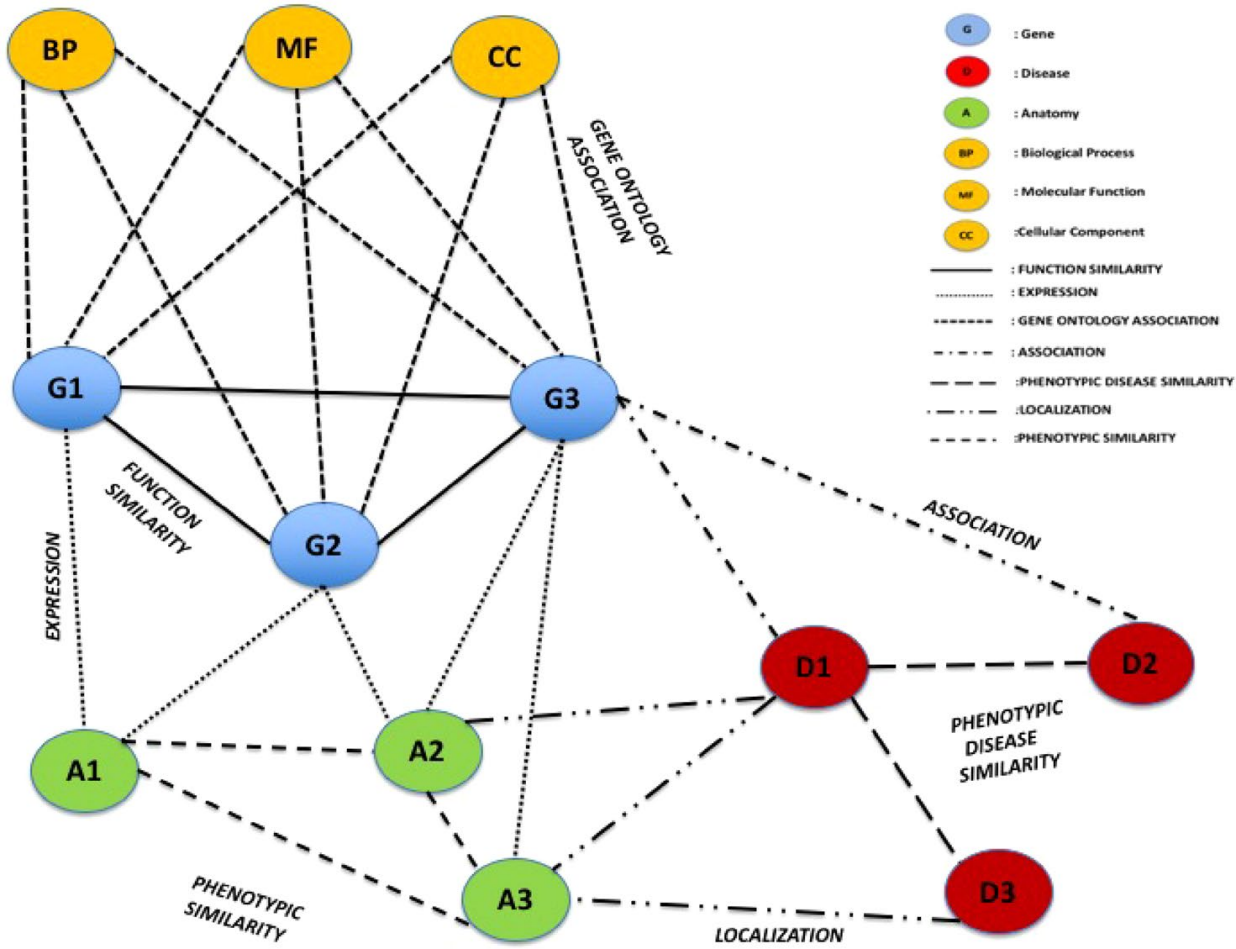
The Figure depicts an example of a heterogeneous network. The heterogeneous network contains different types (or colours) of nodes and different kinds of edge. In the given network, different node colours represent different medical data such as genes, diseases, anatomies, biological processes, molecular functions, cellular components, and different line styles represent different edge types.

Nevertheless, recent discoveries in biology have elucidated that the interplay of molecules of different types (e.g., genes, proteins and ribonucleic acids^7,8^) are constitutive blocks of mechanisms inside cells. Consequently, models describing the interplay should be able to consider the presence of multiple different agents and associations, i.e. multiple different types of nodes and edges. There is the need to use more complex network models comprising different nodes and different associations among them. Such kind of networks is often referred to as heterogeneous networks that use nodes and edge of different types. For instance, heterogeneous networks may be used to model associations among genes, diseases, anatomies and ontology concepts^9,10^ as depicted in Fig. 1.

A heterogeneous biological network is modeled by a node coloured graph ${G}_{het}=({V}_{het},{E}_{het},C)$, where ${V}_{het}$ is a set of coloured nodes, ${E}_{het}$ $\subseteq$ ${V}_{het}\times {V}_{het}$ is a set of edges, and $C$ is a set of colours that define a coverage of ${V}_{het}$. Once modelled, a set of algorithms may be adapted to analyse such networks for deriving biological insights and solving real problems. Among them, one of the most challenging problems is the comparison of two or more networks through network alignment algorithms. Let ${G}_{1}=\{{V}_{1},{E}_{1}\}$ and ${G}_{2}=\{{V}_{2},{E}_{2}\}$ be two (homogeneous) graphs, where ${V}_{1}$ and ${V}_{2}$ are set of nodes and ${E}_{1}$ and ${E}_{2}$ are set of edges, the **graph alignment problem** consists of finding an alignment relation (or a mapping) $f:{V}_{1}\to {V}_{2}$ such that the similarity between mapped entities is maximised. Thus, the alignment problem relies on the *(sub)-graph isomorphism problem*, which is computationally hard in some general formulations^11^. Algorithms for alignment of networks fall into two main classes: local and global ones. Global Network Alignment (GNA) algorithms try to find a global mapping among all the nodes of the input networks, while Local Network Alignment (LNA) algorithms focus on mapping among (relatively) small single regions of input networks^12^. LNA has been defined in the past for homogeneous networks (LNA${}_{hom}$), and it has been formalised in many papers, such as the first paper by Berg and Lassig^13^ and the different formalisation proposed by Mina and Guzzi^14^. LNA algorithms try to find a mapping among (small) subregions of the input graphs^14^.

Despite the existence of many algorithms for the local alignment of homogeneous networks^12^ (see related work section for a detailed synopsis), they are not able to deal with heterogeneous networks since existing algorithms may process only homogeneous networks. Therefore they fail to discriminate among different node types. The alignment of heterogeneous networks is a relatively new field; Gu *et al*.^15^ presented a novel GNA algorithm for heterogeneous networks, while to the best of our knowledge there are no available LNA algorithms designed for heterogeneous networks. Since the local alignment of networks reveals different knowledge compared to global alignment, there is a need for the introduction of novel LNA algorithms for heterogeneous networks.

**Figure 2:**
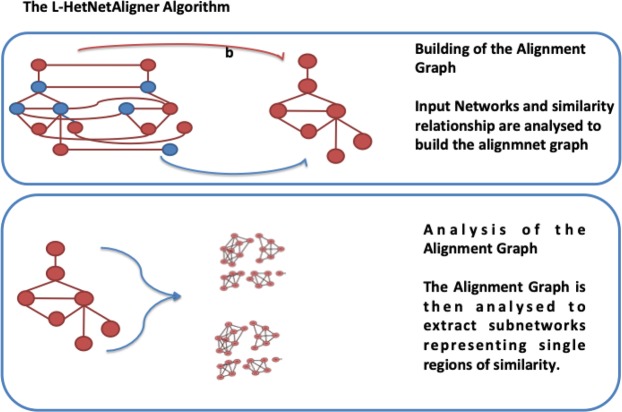
Main steps of the L-HetNetAligner algorithm. In the first step the algorithm integrates two input networks into a single weighted alignment graph. In the second step communities are extracted from the alignment graph. Each community represents a region of local alignment.

Here we propose L-HetNetAligner, a novel algorithm for local alignment of heterogeneous networks by proposing a two-step strategy as depicted in Fig. 2. Our algorithm takes as input two heterogeneous networks modelled as node-coloured graphs and a set of initial similarities among nodes of the networks, and it produces a set of graphs representing single local regions of the alignment. The algorithm merges two input graphs into a single one, named heterogeneous alignment graph that is a single-colour node edge-weighted graph. The nodes of the alignment graph feature pairs of nodes of the input ones. The initial list of node similarities is used to build these nodes. Then, the input graphs are analysed as described in the following to add edges of the heterogeneous alignment graph. Finally, the algorithm uses the Markov clustering (MCL) algorithm^16^ to cluster the graph. Each extracted module represents a single region of the alignment. The result of our algorithm is a list of mapping among a subset of nodes of two networks, i.e. a set of mapped regions among input graphs. We proposed a preliminary implementation of this method in^17^ on a high-performance platform. We here refined such implementation even in a sequential fashion, and we provide deeper experimentation on a larger dataset.

We test our method on synthetic networks to demonstrate that it can recover regions of similarity and to demonstrate that the use of colours improves the quality of the alignment. We also present the experimental result on a real biological network obtained from the HetioNet database^18^ demonstrating the usefulness of our approach.

## Our Contribution

We introduce our contribution using an example; then we will discuss its formalisation. L-HetNetAligner has two main steps: (i) construction of the heterogeneous alignment graph, (ii) mining of the alignment graph. Initially, it takes as input two heterogeneous networks and a set of similarities between node pairs. Then, L-HetNetAligner creates the nodes of the alignment graph. Each node of the alignment graph represents a pair of nodes of input networks. The selection of node pairs is guided by the input similarity relationships. Therefore each node is matched with the most similar node of the other network; and each node of the alignment graph represent a pair of similar among nodes from the input networks. Once that all nodes have added to the graph, L-HetNetAligner creates the edges of the alignment graph. Edges are weighted according to the colours of corresponding nodes and to topological considerations. The presence of an edge in the alignment graph is determined by the analysis of the input networks as detailed in the following. Once the alignment graph is built, we use the Markov clustering algorithm (MCL)^16^ to uncover relevant modules.

### Example

**Figure 3:**
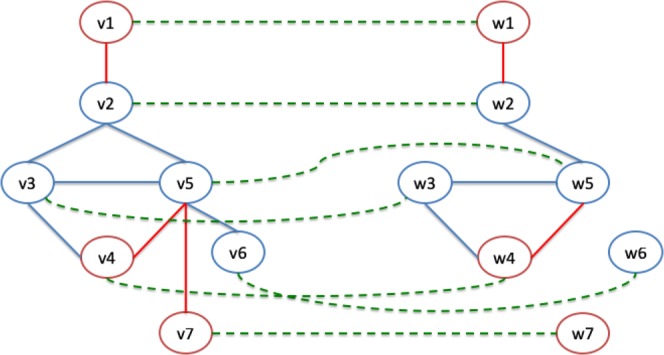
L-HetNetAligner Algorithm: Construction of the Alignment Graph from the Input Graphs. Alignment Example: The Algorithm receives as input two heterogeneous networks and a set of similarity relationships among nodes of networks (green dashed lines). First, the algorithm builds the nodes of the heterogeneous alignment graph. Then edges are added on the basis of the analysis of input networks. The heterogeneous alignment graph has nodes coloured while edges are weighted and with a single colour.

We explain how our algorithm builds an alignment graph through an example. Let consider two input graphs ${G}_{1}=(V,{E}_{1})$, and ${G}_{2}=(W,{E}_{2})$, as depicted in Fig. 3. The proposed algorithm builds the alignment graph by considering both the input graphs and a set of relations of similarity among nodes used as the seed. Node colours represent two different types of nodes. For simplicity, networks have the same number of nodes.

Figure 3  shows these relationships as dashed green lines connecting nodes of two graphs. Initially, for each pair of nodes that are in a relationship, the algorithm builds a new node as depicted in Fig. 3. As evidenced, the nodes of the alignment graph depict the matches of correspondent nodes. To make this simple, we take into account a couple of nodes with the same colour. Therefore the colour of the nodes of the alignment graph is trivially derived. Our algorithm is easily extensible to manage a pair of heterogeneous nodes.

Once that all the nodes have been added to the graph, the algorithm builds the edges among them. For each pair of nodes, it examines the two input graphs. Let us consider the pair of nodes $(v1-w1)$ and $(v2-w2)$ in Fig. 3. To determine the presence of an edge, we must consider the edges $(v1,v2)\in {G}_{1}$ and $(w1,w2)\in {G}_{2}$ and the colour of related nodes. If ${G}_{1}$, and ${G}_{2}$ contains these nodes, and v1 and v2 have a different colour; therefore, there is a **heterogeneous match**. Let us consider nodes $(v2-w2)$ and $(v5-w5)$. The initial graph contains both the edges connecting their internal nodes and all the nodes have the same colour. Therefore there is a **homogeneous match**, and the edge is inserted into the graph.

Let us consider $\Delta =2$ as node distance (i.e. the length of the shortest connecting path) threshold to discriminate between gaps and mismatches. Let us consider the pair of nodes $(v5-w5)$ and $(v6-w6)$. ${G}_{1}$ contains the edge (v5,v6) while nodes w5 and w6 are disconnected in ${G}_{2}$. Therefore there is a **homogeneous mismatch**. There is a **heterogeneous mismatch** among nodes $(v5-w5)$ and $(v7-w7)$. A **homogeneous gap** is established among $(v2-w2)$, and $(v3-w3)$ since $v2$ and $v3$ are adjacent in ${G}_{1}$ while $w2$ and $w3$ have a distance equal to 2. Similarly, there is a **heterogeneous gap** among $(v3-w3)$, and $(v4-w4)$. After the analysis of all pair of nodes, the final alignment graph is built as represented in Fig. 3. The analysis of this graph through algorithms for community detection or clustering is the second step of our algorithm.

### L-HetNetAligner Algorithm

A heterogeneous biological network is represented by a node coloured graph ${G}_{het}=({V}_{het},{E}_{het},C)$, where ${V}_{het}$ is a collection of coloured nodes, ${E}_{het}$ $\subseteq$ ${V}_{het}\times {V}_{het}$ is the collection of edges, and $C$ is a collection of colours that define a coverage of ${V}_{het}$ as represented in Fig. 1. We expand the definition proposed by^19^; thus, let two heterogeneous networks ${G}_{het1}=({V}_{het1},{E}_{het1},C)$ and ${G}_{het2}=({V}_{het2},{E}_{het2},C)$, a subset of node pairs $L\subseteq {V}_{het1}\times {V}_{het2}$, induces a local alignment ${L}_{ali}$ of ${G}_{het1}$ and ${G}_{het2}$ according to the scoring function $f$ that measures the similarity among nodes of two input networks $F:{V}_{het1}\times {V}_{het2}\to [0,1]$, considering three conditions: match, mismatch and gap.

### Step 1: Creation of the Heterogeneous Alignment Graph

**Figure 4:**
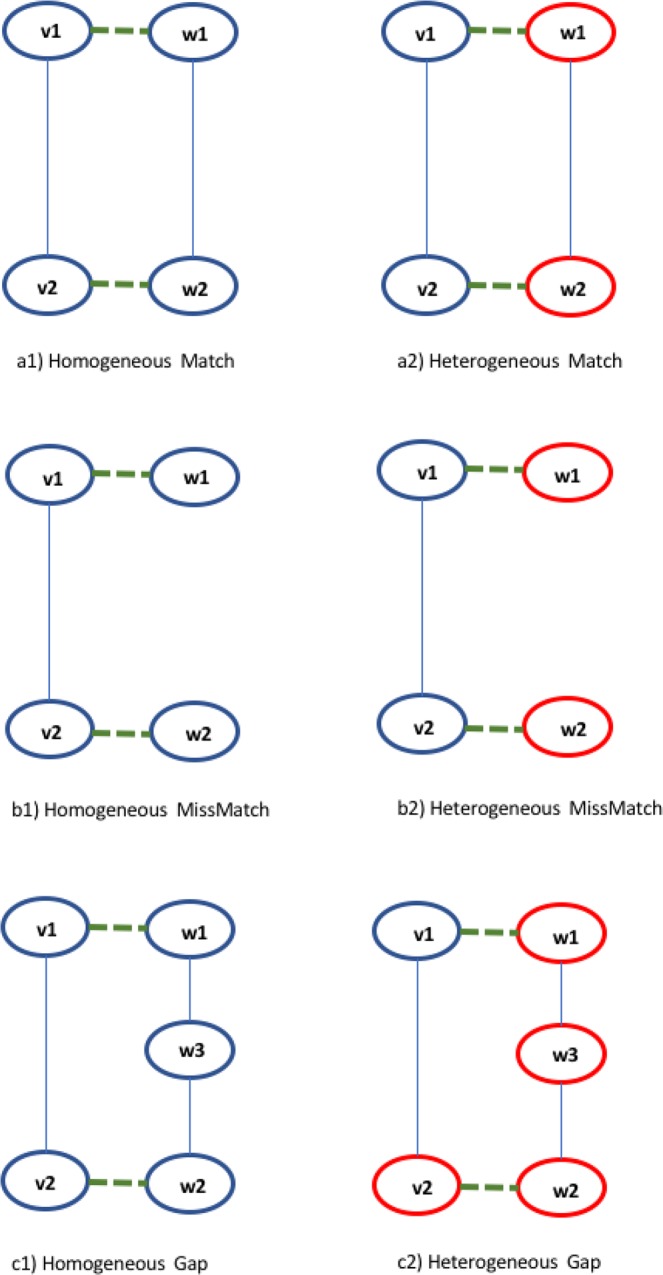
Example of match, mismatch and gap and two possible sub-cases for each one, homogeneous and heterogeneous.

The alignment graph $G=({V}_{al},{E}_{al})$ is a node-coloured graph constructed by two initial input graphs ${G}_{1}=({V}_{1},{E}_{1})$, and ${G}_{2}=({V}_{2},{E}_{2})$. Each node ${v}_{al}\in {V}_{al}$ represents a match of nodes of the input graphs, so ${V}_{al}\subseteq {V}_{1}\times {V}_{2}$. We here focus on the combination of two nodes of the same colour. Nodes of the alignment graph are usually added by considering only pairs of similar nodes while edges are inserted and weighted considering three possible cases as depicted in Fig. 4.

**Match** Given two nodes of the alignment graph ${v}_{al,1}=({v}_{1},{w}_{1})$ and ${v}_{al,2}=({v}_{2},{w}_{2})$, an **homogeneous match** is proved when the input nodes are adjacent and all the nodes have the same colour (Fig. 4(a1)), an **heterogeneous match** is proved when the input nodes are adjacent and the input nodes have a different colour (Fig. 4(a2)).

**Mismatch** Given two nodes of the alignment graph ${v}_{al,1}=({v}_{1},{w}_{1})$ and ${v}_{al,2}=({v}_{2},{w}_{2})$, an **homogeneous mismatch** is proved when the input nodes are adjacent only in a single network and all the nodes have the same colour (Fig. 4(b1)), an **heterogeneous mismatch** is proved when the input nodes are adjacent only in a single network and the input nodes have a different colour (Fig. 4(b2)).

**Gap** Given two nodes of the alignment graph ${v}_{al,1}=({v}_{1},{w}_{1})$ and ${v}_{al,2}=({v}_{2},{w}_{2})$, an **homogeneous gap** is proved when the input nodes are adjacent only in a single network and they are at distance lower than $\Delta$ (gap threshold) in the other network and all the nodes have the same colour (Fig. 4(c1)), an **heterogeneous gap** is proved when the input nodes are adjacent only in a single network and they are at distance lower than $\Delta$ in the other network and the input nodes have a different colour (Fig. 4(c2)).

#### Weighting the Edges

After that the edges of the alignment graph are added, a weight is assigned to each edge by applying an ad-hoc scoring function $F$ and the gap threshold $\Delta$. This function should favor matches and should discourage mismatch and gaps. The kind of the scoring function has a large significance on the resulting alignment graph and on the alignment itself. We here present some experiments using some parameters. The software we implemented enables the user to choose other values to optimise the quality of results as we discuss later.

### Step 2: Analysis of the Heterogenous Alignment Graph using MCL

As introduced before the clustering of the alignment graph is done by applying the MCL algorithm. The markov cluster algorithm works by simulating a stochastic (Markov) flow in a weighted graph, where each node is a data point, while the adjacency matrix stores the edge weights. When the algorithm converges, it produces the new edge weights that define the new connected components of the graph (i.e. the clusters). A cluster on a network is defined as a set of nodes that are more closely connected among them than to the other nodes of the network. Thus, a random walk starting inside a cluster tends to remain inside it rather than to go outside. MCL produces a non-overlapping partitioning of the network by simulating a stochastic flow as described in^16^.

It consists of two steps: *expand* and *inflate*. In the *expand step*, MCL reproduces stochastic flow from a node to likely new nodes, especially enhancing the flow to those nodes that are achievable by multiple and short paths. In the *inflation step*, MCL increases the flows within the clusters and decreases flows among different clusters. Therefore, the initial flows, quite uniform, becomes non-uniform, causing the evolution of a cluster structure, i.e. local regions with a high level of flow. The inflation process is directed by the **inflation parameter**. This parameter is inversely proportional to the size of clusters: the higher inflation parameter rides, the smaller the average dimension of clusters. Finally, MCL is able to find clusters on graphs, robust to noise and graph alterations.

### Workflow of the Algorithm

We now recapitulate the steps of the algorithm:

1. **Building of the Alignment Graph**: The algorithm receives as input two node-coloured graphs, and a similarity function among nodes and it constructs a weighted alignment graph.
2. **Analysis of the Alignment Graph**: The alignment graph is then mined to discover communities applying an existing clustering approach: the Markov clustering algorithm^16^ that produces as output a non-overlapping partition of nodes. It works by simulating a stochastic (Markov) flow in a graph, where each node is a data point. When the algorithm converges, it produces the new edge weights that define the new connected components of the graph (i.e. the clusters). In a network, a cluster is a group of nodes that are highly connected with respect to other nodes of the network.

**Figure 5:**
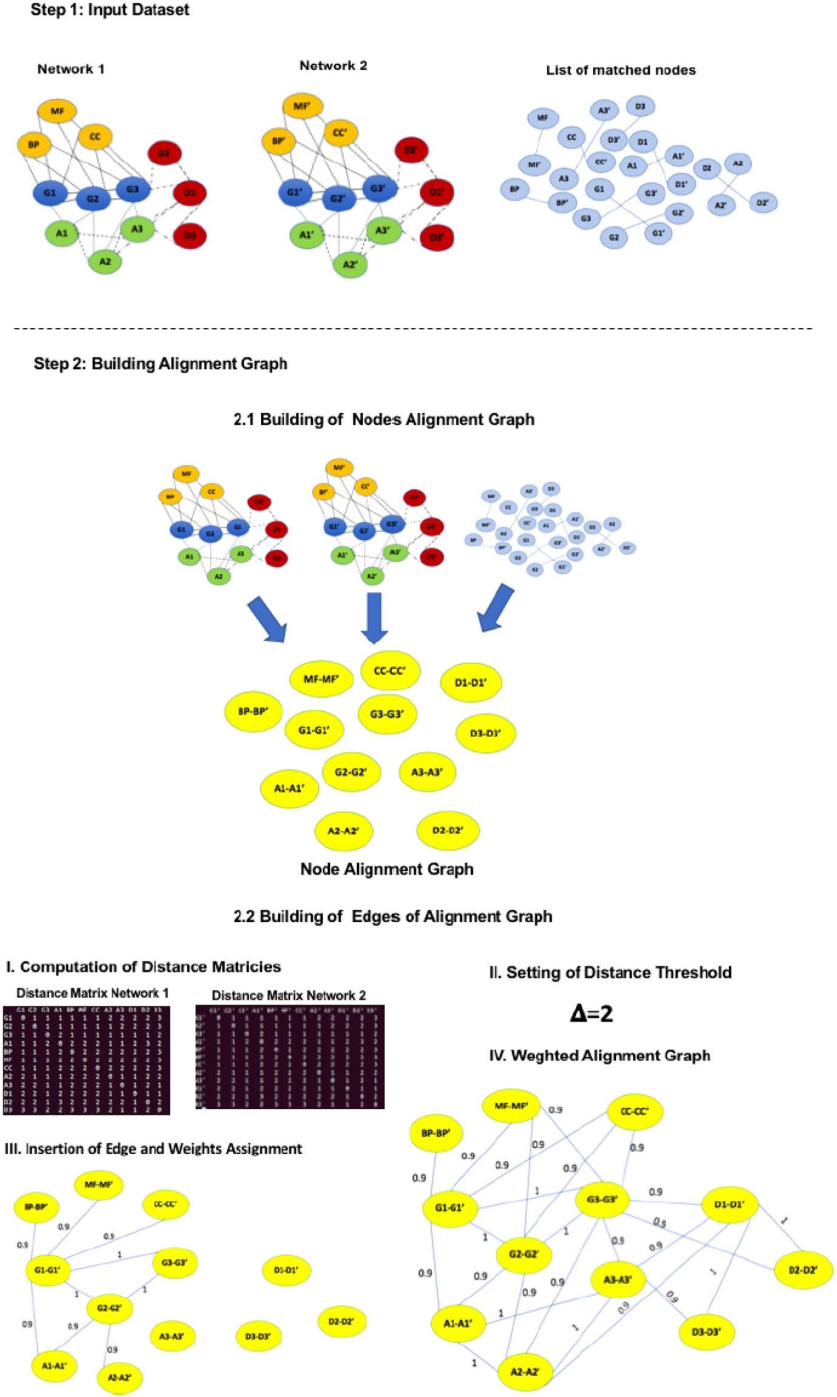
Algorithm workflow. In Step 1, the algorithm takes as input two heterogeneous networks and a subset of node pairs matched according to a similarity function. In Step 2 the algorithm builds the weighted alignment graph: in step 2.1 the algorithm defines the nodes of the alignment graph represented by the pair matched nodes; in step 2.2 the algorithm computes a distance matrix for each input network and sets a distance threshold $\Delta$. According to these, the algorithm inserts and weights the edges of the alignment graph. Once that the weighted alignment graph is built we mine it using the Markov clustering algorithm (MCL). The local alignment is the union of all the modules extracted by MCL from the alignment graph.

 Figure 5 shows the workflow of the algorithm, while Algorithm 1 shows the pseudocode of L-HetNetAligner.

**Algorithm 1:**
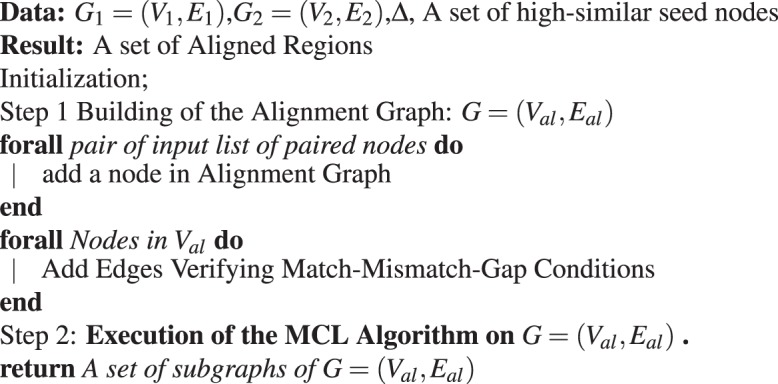
Heterogeneous Probability Model (HPM).

Thus, we evaluate L-HetNetAligner considering the alignment of a network with respect to itself, and considering the alignment of a network with respect to an altered version of the network obtained by adding different levels of noise (5%, 10%, 15%, 20% and 25%). We also test the impact of colours on the alignment, and we consider the presence of colours considering a network with one, two, three, and four colours. We aim to demonstrate the ability of our algorithm to build high-quality alignments and to demonstrate that the quality of the alignment increases when considering network colours.

The evaluation of the quality of the alignments is computed by counting the fraction of nodes and edges that are *correctly* mapped, i.e. the fraction of nodes and edges correctly aligned to the true node (and edge) mapping. For global network alignment, the node correctness (NC) measure^20^ evaluates the ability to recover the true node mapping. As noted in Meng *et al*.^21^ NC has not been defined for local alignment, and they propose three novel measures: Precision, Recall, and F-score of node correctness (P-NC, R-NC, and F-NC, respectively). The evaluation of above measures is possible only when the true node mapping is well-known. Let suppose the alignment $f$ produces a set of node pairs composed by ${N}_{al}$ nodes while the true node mapping is composed of ${M}_{tr}$ nodes.

*P-NC* is calculated as as $\frac{{M}_{tr}\ \cap \ {N}_{al}}{{M}_{tr}}$. *R-NC* is defined as $\frac{{M}_{tr}\ \cap \ {N}_{al}}{{N}_{al}}$. *F-NC*, is a combination of *P-NC* and *R-NC*. In parallel we also compute how well edges are correctly mapped by an alignment considering the true edge mapping. Among the other existing measures, we compute the NCV-G${S}^{3}$ measure^21^. NCV-G${S}^{3}$ is the geometric mean of the two individual measures: node coverage (NCV) and Generalized ${S}^{3}$ (G${S}^{3}$).

Let ${G}_{1}=\{{V}_{1},{E}_{1}\}$ and ${G}_{2}=\{{V}_{2},{E}_{2}\}$ be two graphs, where ${V}_{1,2}$ are sets of nodes and ${E}_{1,2}$ are sets of edges. Let ${G}_{1}^{{\prime} }=\{{V}_{1}^{{\prime} },{E}_{1}^{{\prime} }\}$ and ${G}_{2}^{{\prime} }=\{{V}_{2}^{{\prime} },{E}_{2}^{{\prime} }\}$ be subgraphs of ${G}_{1}$ and ${G}_{2}$ that are induced by the mapping. NCV is the percentage of nodes from ${G}_{1}$ and ${G}_{2}$ that are also in ${G}_{1}^{{\prime} }$ and ${G}_{2}^{{\prime} }$ subgraphs: $NCV=\frac{{V}_{1}^{{\prime} }+{V}_{2}^{{\prime} }}{{V}_{1}+{V}_{2}}$. G${S}^{3}$ is the percentage of correctly mapped edges ${N}_{c}$ with respect to the total of both correctly mapped ${N}_{c}$ and non-correctly mapped edges ${N}_{n}$ with respect to the true edge mapping: $G{S}^{3}=\frac{{N}_{c}}{{N}_{c}+{N}_{n}}$. We compute the P-NC, R-NC, F-NC, NCV, G${S}^{3}$ and NCV-G${S}^{3}$ for local alignments of synthetic networks and Hetionet network by applying the software for NA evaluation proposed in^21^ (see Tables in Supplementary File 1). Since F-NC and NCV-GS3 derived from the mixing of two measure, we performed the analysis by considering only these two measures.

Consequently, we have constructed the noisy counterparts (1) for each of the heterogeneous synthetic network versions with one, two, three, and four colours and (2) for the Hetionet network with one two, three, and four colours. Then, we apply L-HetNetAligner to build the alignment of each synthetic network with its counterparts.

Regarding the noisy networks, we performed both adding/removing nodes and edges. In the paper we present only results related to edge removal. Other results obtained considering adding/removing nodes and adding edges are presented in Supplementary File 1.

We executed the experiments on an Intel Xeon(R) Processor (3.4 GHz, 4 core, and 8 threads) with 16 Gbytes of memory running an Ubuntu OS ver 18.04. We implement our algorithm using the Python programming language^22^.

We also performed other experiments by generating different synthetic networks having a different structure. In particular we generated five scale-free networks with 5000, 25000, 50000, 75000, 95000 nodes, five geometric networks with 5000, 25000, 50000, 75000, 95000 nodes, five Erdos-Renyi networks with 5000, 25000, 50000, 75000, 95000 nodes, five small-world networks with 5000, 25000, 50000, 75000, 95000 nodes. We reported the results in Supplementary File 1 - Section Experiments on different Network Models.

The code is available for academic purposes at https://sites.google.com/view/heterogeneusnetworkalignment. The network analysis is performed using the igraph libraries^23^.

### Dataset: Synthetic Networks

We generated twelve synthetic networks with scale-free networks (SF)^24^ graph generator. All the networks have 950 nodes while the edges are distributed as follows: Network 1 has 3410 edges, Network 2 has 3420, Network 3 has 3340, Network 4 has 3200, Network 5 has 3530, Network 6 has 3330, Network 7 has 3340, Network 8 has 3380, Network 9 has 4490, Network 10 has 4060, Network 11 has 4380 and Network 12 has 4160. Then, we assign randomly a colour among *k* available ones to each node. We vary *k* from one to four with the aim to obtain 4 heterogeneous variants of all synthetic networks as follows:

- 1 coloured version;
- 2 coloured version (in which 460 nodes present one colour and 490 nodes have another colour);
- 3 coloured version where we randomly assign one colour to 370 nodes, a second colour to 300 nodes and a third colour to 280 nodes ;
- 4 coloured version where we randomly assign one colour to 170 nodes, a second colour to 250 nodes, a third colour to 330 nodes and a fourth to 200 nodes.

### Dataset: Hetionet Network

Hetionet^18^ is a heterogeneous network integrating data of medical relevance extracted from public resources. Hetionet consists of 47031 nodes of 11 types, such as genes, compounds, diseases, anatomies, pathways, biological processes, molecular functions, cellular components, pharmacologic classes, side effects, and symptoms and 2250197 relationships of 24 types (see^18^ for a complete description). Starting from Hetionet dataset, we create a sub-network composed of 37142 nodes that represent genes, diseases, GO annotations (biological processes, molecular functions and cellular components), and anatomy data. To create the sub-network, we selected the most significant node type (genes, diseases, GO annotations, and anatomy data) in term of numbers and metaedges (the type of relations among nodes). We use the node type to assign a colour to each node of the Hetionet network. We build four coloured version of Hetionet in order to cover each type of nodes as follows:

- one coloured version where all nodes have the same colour;
- two coloured version where we assign one colour to nodes related to GO annotations and the one colour to nodes that are not related to GO annotations; we obtain 15656 GO annotation related and 21486 non-GO annotation related nodes;
- three coloured version where we assign a first colour to nodes related to diseases information, a second color to nodes related to GO annotations, and a third one to the other nodes. We obtain 136 disease-related, 15656 GO annotation related, 21350 non-disease related and non-GO annotation related nodes;
- four coloured version where we assign a different colour to nodes related to genes, to nodes related to anatomical data, to nodes related to disease information, and to nodes related to GO annotations. We obtain 20945 genes related to gens, 405 related to anatomy, 136 disease-related, and 15656 GO annotation related nodes.

Therefore, we obtain four different coloured versions of the Hetionet where each typology of nodes is coloured with a different colour. When adding colours we want to test the ability of our algorithm to obtain better results with respect to the absence of colours.

### L-HetNetAligner Parameters

We set $\Delta$ = 2, and following weights:

- Homogeneous Match: 1
- Heterogeneous Match: 0.9
- Homogeneous Mismatch: 0.5
- Heterogeneous Mismatch: 0.4
- Homogeneous Gap: 0.2
- Heterogeneous Gap: 0.1 .

Then, each edge is weighted according to six cases of homogeneous/heterogeneous match, homogeneous/heterogeneous mismatch and homogeneous/heterogeneous gap. The selection of these parameters has been made after a set of experiments to guarantee best performances. We followed a trial and error approach (data not shown) and for each configuration of the parameters we measured the quality parameters. Then, we choose this configuration since it presented the best results in terms of the measures we used (Supplementary File 1 contains a table showing some data related to the variation of the parameters). It should be noted that user may tune these parameters to improve the quality of the alignments. The choice of these values is a crucial step in our algorithm. For this reason, we set these values as modifiable parameters.

We have computed the NCV-G${S}^{3}$ for local alignments of synthetic networks and Hetionet network by using the software for NA evaluation proposed in^21^. We expect that for a given noise level, the use of colours should improve alignment quality over one node colour. We also expect that the use of more colours will improve the quality of the alignment. Finally, we predict that the use of more colours should make the alignment more robust to noise.

### Topological evaluation

#### Syntethic networks: topological evaluation

**Figure 6:**
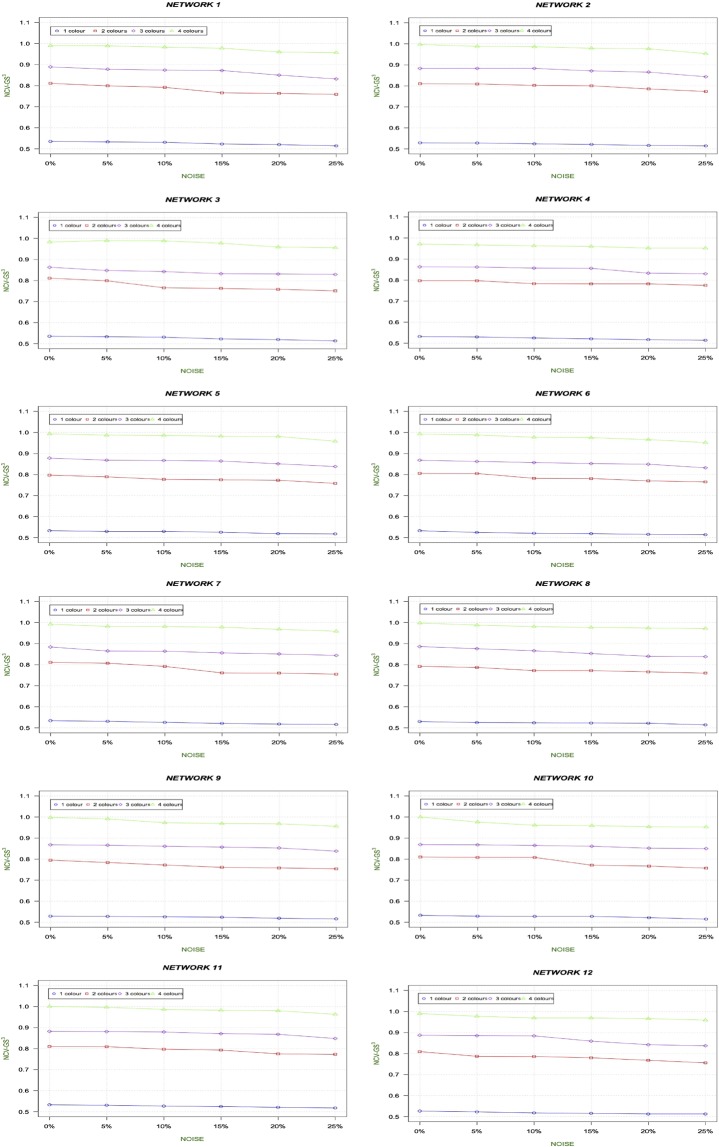
The Figure shows the trend of the NCV-G${S}^{3}$ related to the alignment of the original synthetic network with its noisy version at 0%, 5%, 10%, 15%, 20% and 25% of added noise for all synthetic networks. Results show that for each network, the quality of the alignment increases when considering more colours.

We compute NCV-G${S}^{3}$ measure for each alignment. The Fig. 6 shows the trend of the NCV-G${S}^{3}$ related to the alignment of the original synthetic network with its noisy version (also referred to as altered networks in Tables) at 0%, 5%, 10%, 15%, 20% and 25% of added noise for all synthetic networks. In terms of quality, we expect that for a given noise level, the more colours are used, the better the alignment quality should be. Moreover, the use of colours should also improve the robustness to noise compared to the use of fewer colours.

**Figure 7:**
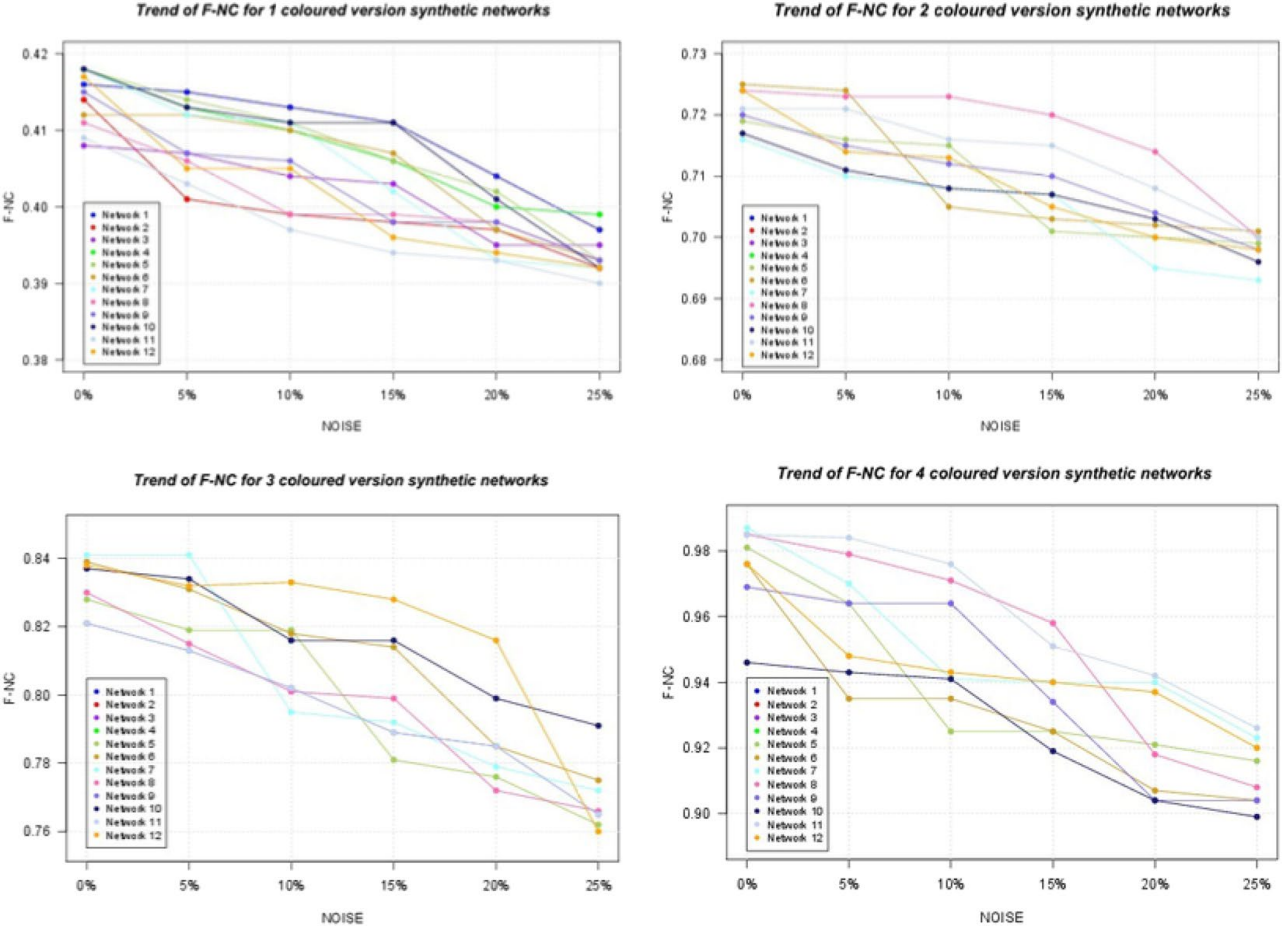
Trend of F-NC for Synthetic Networks. The Figure shows the trend of F-NC for synthetic networks. We note that an increase of F-NC when more colours are used.

The analysis of results in Fig. 6 shows that for a given level of noise the use of colours improves the quality of the alignment. Besides, the robustness to the impact of noise is better. This trend is evident considering both NCV-G${S}^{3}$ as well as node F-NC as reported in Fig. 7, while whole values for P-NC, R-NC and F-NC are reported in the Supplementary File 1.

#### HetioNet network: topological evaluation

**Figure 8:**
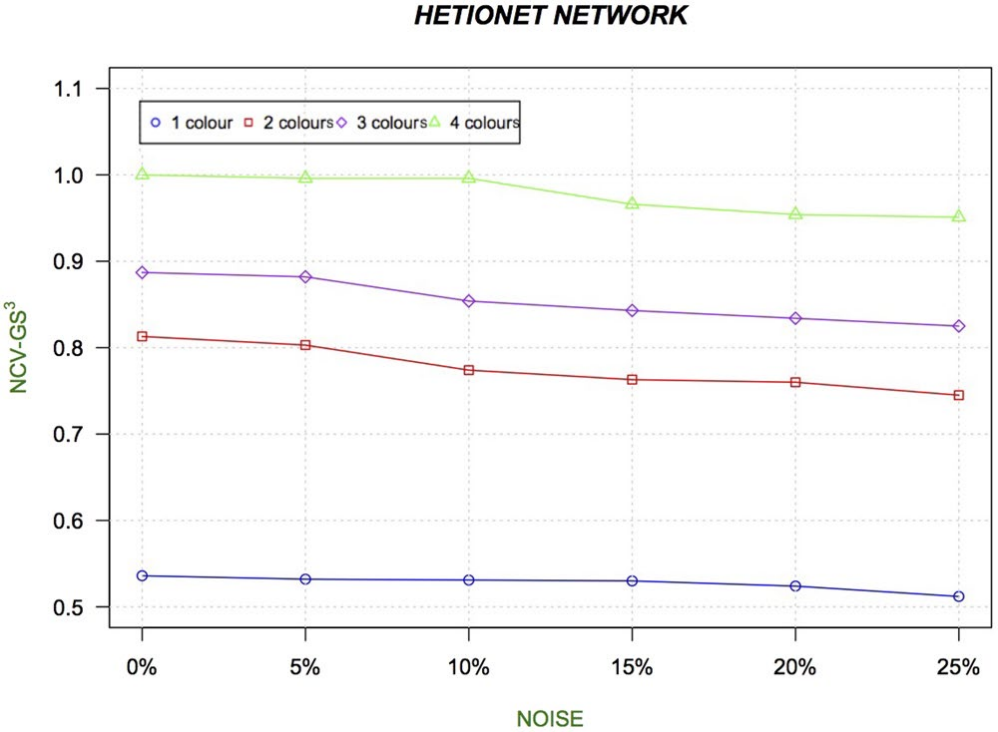
The Figure presents the trend of the NCV-G${S}^{3}$ related to alignment of the original Hetionet network with its counterparts at 0%, 5%, 10%, 15%, 20% and 25% of added noise. Results show that the quality of the alignment increases when considering colours.

**Figure 9:**
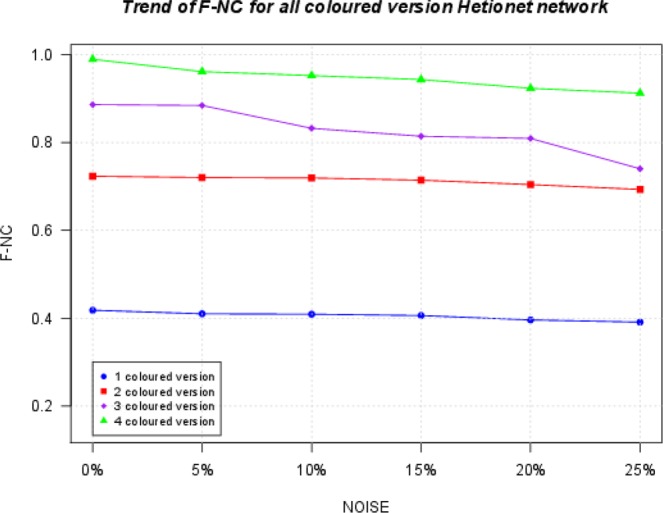
Trend of F-NC for Hetionet Networks. The figure shows that the F-NC increases when more colours are used.

We compute NCV-G${S}^{3}$ and F-NC measures for Hetionet network. Figure 8 and Fig. 9 show the trend of the NCV-G${S}^{3}$ and F-NC related to alignment of the original Hetionet network with its noisy version at 0%, 5%, 10%, 15%, 20% and 25% of added noise. Results show that the quality of the alignment increases when considering colours. Furthermore, increasing noise level from 5 % to 25 % into the original networks causes NCV-G${S}^{3}$ and F-NC to decrease.

We should note (Figs. 6 and 8) that the NCV-G${S}^{3}$ values increase when increasing the number of colours, showing the best results in 4 coloured versions. Furthermore, we compute F-NC measure and we show that F-NC values increase when considering colours as depicted in Fig. 9 (see Tables related to these measures for Hetionet network in Supplementary File 1). In terms of accuracy of the alignments, results for both synthetic networks and Hetionet Network, for a given noise level, show that the heterogeneous alignment improves the alignment quality over homogeneous alignment (i.e. one node colour) (see Figs. 7, 9, 8, and 6). Results also show that the number of colours used causes the increasing of the quality of the alignments and the robustness to the noise.

### Functional Quality Evaluation

We also evaluate the quality of results by assessing the biological relevance of extracted modules from Hetionet network. In general, groups of related entities should have a similar biological role or share some functions^14^. To test the relatedness of a group of biological entities, i.e. genes or proteins, ontologies and measures of similarity based on ontologies have been proposed. We use here Semantic Similarity (SS) measures^25^ to address these problems. SS measures are used to quantify the functional similarity of pairs or groups of biological entities, comparing the annotations extracted from biological ontologies such as Gene Ontology^26^. We start from the consideration that biologically related entities are likely to have high semantic similarity, similarly as proposed in^14^.

**Figure 10:**
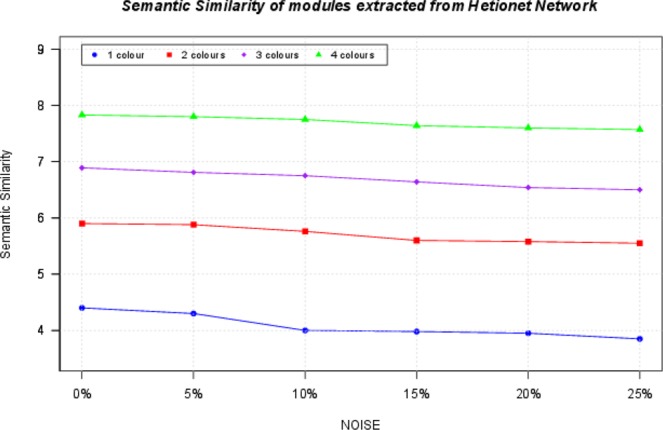
The Figure shows the average semantic similarity of modules. SS measures are used to quantify the functional similarity among biological entities using the annotations contained in biological entities. For each module, we extracted functional annotations contained in Gene Ontology. Then we used the Resnick measure to evaluate the semantic similarity of each module by considering all pairs of entities inside. Finally, we averaged this quantity for all the modules of an alignment. The figure shows that considering colours of the networks produce better results in terms of functional similarity.

Given a solution ${S}_{k}$ (i.e. a module extracted from MCL on the heterogeneous alignment graph), we compute the SS among all the pairs of its entities. We use the Resnik’s SS measure^27^ with the Best-Match Average (BMA) approach. Figure 10 reports an overview of semantic similarity values of modules obtained by aligning Hetionet network with its noisy counterparts for each coloured version. As seen, the 4 coloured version presents the highest semantic similarity than other coloured versions. This demonstrates that modules extracted by aligning the Hetionet network with more node colours are better in terms of semantic similarity than those extracted from aligning the Hetionet networks with a single node colour.

Moreover, we compare each calculated alignment versus random one, with the aim to demonstrate its statistical significance. Formally, given a solution ${S}_{i}$, we can test the null hypothesis ${H}_{0}^{1}$: *the inter-species semantic similarity*$S{S}_{i}({S}_{i})$*is drawn from the background distribution*, where the background distribution can be assessed from the $S{S}_{i}$ of random solutions. Usually, the hypothesis is rejected when the p-value results lower than $0.001$. All the solutions provided by the algorithm have a value of semantic similarity higher than by chance.

### Link Prediction Evaluation

To evaluate the ability of our algorithm to predict missing link we remove some edges from a network in a random way. Then we align this network to the original one and we measure how many missing links are predicted. Because some edges are missing compared to the original set of node pairs, the algorithm should be able to find the missing links as gaps or mismatch. Finally, we count how many homogeneous and heterogeneous gaps/mismatches are found in the alignment graph representing the inferred links that lack in the original input networks. For each predicted link we tested the correctness by verifying the presence of the edge into the input network. Table 1 reports the number of correctly predicted links obtained by aligning original synthetic network with those obtained when removing randomly pair-matched nodes for all the networks.

**Table 1:** The amount of corrected predicted links computed by aligning the original synthetic network with its noisy counterparts for all the networks. For each synthetic network we removed randomly an increasing number of edges (5%,10%,15%,20%,25%) and then we ran the L-HetNetAligner.

**Network**	**Colours**	**5% of noise**	**20% of noise**	**15% of noise**	**20% of noise**	**25% of noise**
N1	1	20	50	70	80	110
	2	80	130	200	210	280
	3	130	230	320	330	370
	4	190	360	490	550	600
N2	1	21	52	72	81	130
	2	83	131	201	213	282
	3	132	233	322	332	371
	4	193	364	492	551	604
N3	1	19	51	73	81	130
	2	79	130	204	214	282
	3	128	233	323	333	370
	4	189	359	492	552	603
N4	1	17	48	69	78	109
	2	75	128	198	201	278
	3	122	227	318	329	366
	4	185	258	488	548	596
N5	1	25	52	71	81	115
	2	86	133	202	212	283
	3	137	233	322	334	372
	4	194	362	493	552	601
N6	1	18	48	68	79	108
	2	76	125	197	201	276
	3	124	222	316	327	369
	4	186	354	488	549	596
N7	1	19	48	67	78	107
	2	77	124	196	205	277
	3	123	223	315	329	366
	4	185	356	487	548	597
N8	1	18	47	74	79	109
	2	77	121	205	209	279
	3	123	226	323	330	368
	4	184	357	494	549	599
N9	1	27	53	76	82	117
	2	88	133	203	214	284
	3	139	237	321	333	375
	4	198	365	494	552	603
N10	1	27	55	76	83	117
	2	87	137	204	214	285
	3	135	239	323	333	377
	4	197	368	495	555	606
N11	1	28	56	78	82	115
	2	86	135	204	211	288
	3	133	238	322	332	374
	4	195	362	493	554	605
N12	1	25	58	76	81	116
	2	87	134	203	210	289
	3	130	239	321	335	373
	4	192	336	494	553	607

We should note L-HetNetAligner can predict a high number of link for each synthetic network. Thus, our algorithm can extract knowledge about the aligned networks.

### Time Consumption and Memory Occupancy

L-HetNetAligner completed the process of alignment of synthetic networks in almost 15 minutes, and the process occupies 4 GB of Memory. L-HetNetAligner completed the process of alignment of Hetionet network in almost 50 minutes, and the process occupies 4 GB of Memory. Table 2 presents the time of execution for obtaining the alignment graph and the final local alignment with MCL for all heterogeneous synthetic networks. The Execution Times is equal for all heterogeneous versions.

**Table 2:** Execution Time of L-HetNetAligner to construct the alignment graph and Execution Time of MCL to extract relevant modules.

**Network**	**L-HetNetAligner**	**MCL**
N1	15 minutes	1 minute
N2	15 minutes	1 minute
N3	15 minutes	1 minute
N4	15 minutes	1 minute
N5	16 minutes	1 minute
N6	17 minutes	1 minute
N7	17 minutes	1 minute
N8	17 minutes	1 minute
N9	20 minutes	1 minute
N10	20 minutes	1 minute
N11	20 minutes	1 minute
N12	20 minutes	1 minute
Hetionet	50 minutes	1 minute

### Comparison with respect to Homogeneous Aligners

In this section we present the comparison of L-HetNetAligner with two homogeneous local alignment algorithms, AlignMCL and AlignNemo. The aim is to demonstrate that L-HetNetAligner reports the best performance to analyse heterogeneous networks with respect to classical homogeneous aligners. For this reason, we forced AlignMCL and AlignNemo to build the alignment of heterogeneous networks. The dataset that we used for the comparison consists of 12 synthetic networks used for L-HetNetAligner evaluation with two colours version. For each synthetic network, we built the noisy version obtained by removing 5%, 10%, 15%, 20% and 25% of edges. Then we built the alignment of a network with respect to itself, and considering the alignment of a network with respect to an altered version by applying L-HetNetAligner, AlignMCL and AlignNemo. We construct the local alignment by applying the default parameters of AlignMCL: Pruning Threshold equal to $0.5$ and Inflation Parameter equal to $2.8$. AlignMCL produces as output a local alignment as a set relevant modules in 22 minutes.

Then, we construct the local alignment with AlignNemo by setting the following parameters: Pruning Threshold equal to $0.5$ and k-sub-graph equal to $4$. The output consists of local alignments as relevant modules. AlignNemo builds the alignment in 35 minutes.

For each alignment built with L-HetNetAligner, AlignMCL and AlignNemo we computed the NCV-G${S}^{3}$ and F-NC measures. Then, we compare NCV-G${S}^{3}$ and F-NC measures obtained from local alignment built with AlignMCL and AlignNemo with NCV-G${S}^{3}$ and F-NC measures obtained from local alignment of L-HetNetAligner in two coloured version.

 Table 3 reports the NCV-G${S}^{3}$ measure comparison among L-HetNetAligner, AlignMCL and AlignNemo. Table 4 reports the F-NC measure comparison among L-HetNetAligner, AlignMCL and AlignNemo. The NCV-G${S}^{3}$ and F-NC measures obtained from local alignment built with L-HetNetAligner and AlignMCL are quite similar and they outperform the values obtained with AlignNemo. However, the NCV-G${S}^{3}$ and F-NC measures for L-HetNetAligner are slightly higher respect AlignMCl. Results show clearly that the use of L-HetNetAligner outperforms classical homogeneous local algorithms.

**Table 3:** NCV-G${S}^{3}$ values computed by aligning the original synthetic network with its noisy versions (obtanied when removing edges) for all the networks by using L-HetNetAligner, AlignMCl and AlignNemo.

**Network**	**Altered Networks (Percentage of removed edges)**	**L-HetNetAligner**	**AlignMCl**	**AlignNemo**
N1	0	0.535	0.529	0.444
	5	0.533	0.528	0.44
	10	0.531	0.527	0.439
	15	0.523	0.527	0.435
	20	0.52	0.524	0.425
	25	0.514	0.521	0.421
N2	0	0.529	0.529	0.449
	5	0.528	0.528	0.446
	10	0.524	0.525	0.444
	15	0.521	0.524	0.432
	20	0.516	0.523	0.424
	25	0.514	0.522	0.421
N3	0	0.535	0.527	0.448
	5	0.533	0.523	0.447
	10	0.531	0.522	0.439
	15	0.523	0.52	0.439
	20	0.52	0.513	0.424
	25	0.514	0.511	0.422
N4	0	0.532	0.53	0.435
	5	0.53	0.528	0.435
	10	0.525	0.519	0.431
	15	0.521	0.517	0.426
	20	0.517	0.514	0.426
	25	0.514	0.514	0.422
N5	0	0.533	0.525	0.445
	5	0.53	0.523	0.44
	10	0.53	0.52	0.436
	15	0.526	0.518	0.434
	20	0.519	0.516	0.428
	25	0.518	0.516	0.422
N6	0	0.533	0.526	0.444
	5	0.525	0.524	0.435
	10	0.521	0.52	0.431
	15	0.519	0.519	0.427
	20	0.516	0.517	0.427
	25	0.514	0.516	0.422
N7	0	0.534	0.528	0.448
	5	0.531	0.518	0.438
	10	0.526	0.516	0.433
	15	0.521	0.516	0.432
	20	0.518	0.515	0.421
	25	0.516	0.514	0.421
N8	0	0.53	0.527	0.45
	5	0.525	0.526	0.448
	10	0.524	0.519	0.435
	15	0.523	0.518	0.429
	20	0.522	0.512	0.428
	25	0.514	0.511	0.426
N9	0	0.529	0.529	0.445
	5	0.528	0.529	0.443
	10	0.526	0.519	0.441
	15	0.524	0.519	0.428
	20	0.519	0.515	0.422
	25	0.516	0.512	0.42
N10	0	0.533	0.528	0.448
	5	0.529	0.526	0.443
	10	0.528	0.523	0.427
	15	0.528	0.518	0.427
	20	0.522	0.514	0.425
	25	0.515	0.511	0.424
N11	0	0.533	0.525	0.446
	5	0.531	0.524	0.444
	10	0.527	0.524	0.438
	15	0.525	0.52	0.436
	20	0.521	0.514	0.431
	25	0.518	0.511	0.423
N12	0	0.527	0.529	0.448
	5	0.523	0.52	0.445
	10	0.518	0.519	0.436
	15	0.516	0.519	0.426
	20	0.513	0.511	0.424
	25	0.513	0.51	0.422

**Table 4:** F-NC values computed by aligning the original synthetic network with its noisy versions (obtained when removing edges) for all the networks by using L-HetNetAligner, AlignMCl and AlignNemo.

**Network**	**Altered Networks (Percentage of removed edges)**	**L-HetNetAligner**	**AlignMCl**	**AlignNemo**
N1	0	0.416	0.414	0.31
	5	0.415	0.411	0.31
	10	0.413	0.411	0.309
	15	0.411	0.405	0.307
	20	0.404	0.404	0.307
	25	0.397	0.399	0.302
N2	0	0.414	0.415	0.318
	5	0.401	0.408	0.313
	10	0.399	0.403	0.311
	15	0.398	0.402	0.31
	20	0.397	0.402	0.308
	25	0.392	0.397	0.305
N3	0	0.408	0.406	0.318
	5	0.407	0.405	0.316
	10	0.404	0.403	0.315
	15	0.403	0.402	0.314
	20	0.395	0.398	0.311
	25	0.395	0.395	0.306
N4	0	0.418	0.414	0.319
	5	0.413	0.411	0.31
	10	0.41	0.402	0.308
	15	0.406	0.399	0.306
	20	0.4	0.398	0.305
	25	0.399	0.397	0.302
N5	0	0.418	0.413	0.317
	5	0.414	0.407	0.312
	10	0.411	0.404	0.31
	15	0.406	0.403	0.306
	20	0.402	0.403	0.305
	25	0.393	0.399	0.304
N6	0	0.412	0.411	0.314
	5	0.412	0.41	0.313
	10	0.41	0.403	0.313
	15	0.407	0.401	0.309
	20	0.397	0.401	0.308
	25	0.393	0.395	0.304
N7	0	0.418	0.415	0.314
	5	0.412	0.414	0.313
	10	0.411	0.413	0.311
	15	0.402	0.409	0.31
	20	0.393	0.409	0.307
	25	0.392	0.396	0.303
N8	0	0.411	0.411	0.32
	5	0.406	0.41	0.316
	10	0.399	0.408	0.316
	15	0.399	0.406	0.314
	20	0.398	0.406	0.311
	25	0.393	0.394	0.306
N9	0	0.415	0.412	0.319
	5	0.407	0.407	0.318
	10	0.406	0.404	0.308
	15	0.398	0.401	0.307
	20	0.398	0.396	0.304
	25	0.393	0.394	0.302
N10	0	0.418	0.4	0.318
	5	0.413	0.395	0.318
	10	0.411	0.393	0.315
	15	0.411	0.393	0.312
	20	0.401	0.393	0.305
	25	0.392	0.392	0.303
N11	0	0.409	0.413	0.315
	5	0.403	0.407	0.314
	10	0.397	0.407	0.308
	15	0.394	0.404	0.302
	20	0.393	0.398	0.301
	25	0.39	0.394	0.301
N12	0	0.417	0.414	0.319
	5	0.405	0.414	0.315
	10	0.405	0.411	0.315
	15	0.396	0.407	0.306
	20	0.394	0.404	0.306
	25	0.392	0.399	0.302

### Related Work

**Figure 11:**
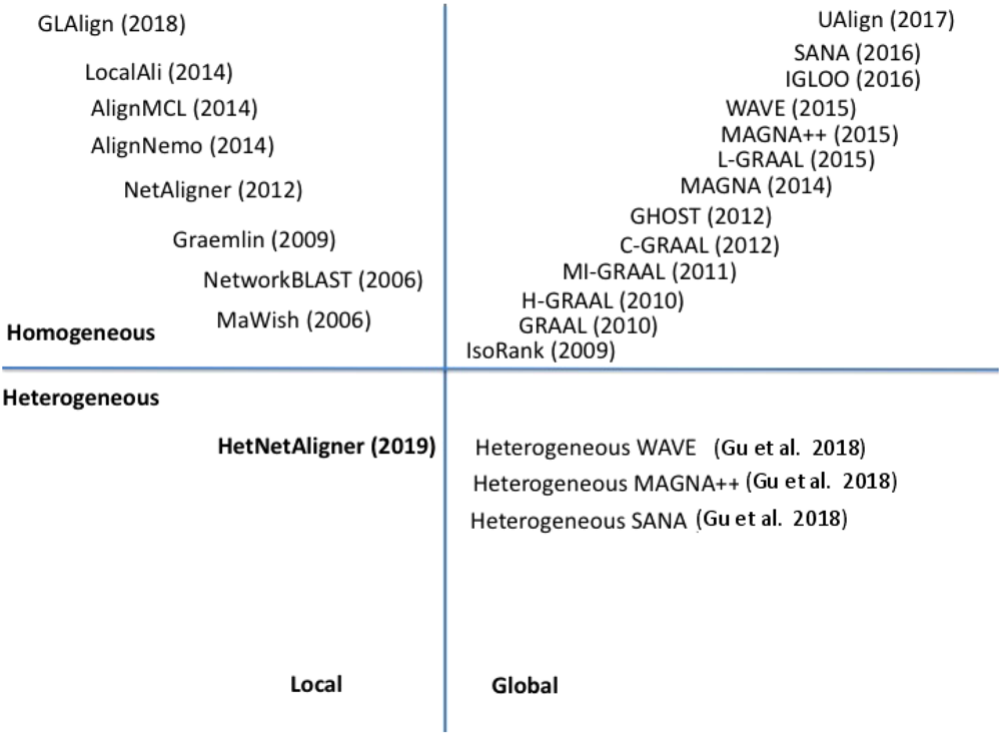
Overview of some Network Alignment Algorithms. Algorithms are classified according to the kind of the alignment (local or global) and the kind of input networks (homogeneous or heterogeneous). The figure also reports the year of implementation. As evidenced heterogeneous approaches are more novel and less frequent.

Network alignment algorithms may be categorised as local or global, and as homogeneous or heterogeneous. Local network alignment algorithms (LNAs) look for the similar small subnetworks by exploiting many-to-many node mapping of the compared networks. The global alignment algorithms (GNAs) search the best superimposition of the whole compared networks by exploiting one-to-one node mapping. Moreover, algorithms may be designed for homogeneous networks or heterogeneous ones. Next, we will present some network alignment algorithms, and we recall all the approaches in Fig. 11.

#### Network Alignment Algorithms

Local Network Alignment algorithms (LNAs) have the goal to discover multiple subnetworks or regions of similarity among input networks. Each region is usually mapped independently of other regions. These regions represent conserved patterns of interaction like conserved motif or pattern of activities.

NetworkBLAST^28^ aims to find small dense regions in protein-protein interaction networks. Such subgraphs represent protein complexes, i.e. set of proteins that perform a analogous function or impaired in the equal biological process. The MaWish algorithm^19^ formulates the network alignment problem as maximum weight induced subgraph, that incorporate a evolutionary design to evaluate topological similarity. Graemlin^29^ searches conserved regions on a pre-computed set of networks. NetAligner^30^ applies a method to determinate evolutionarily conserved interactions, based on the criterion that interacting proteins evolve at rates significantly closer than expected by chance. AlignNemo^31^ enables the discovery of sub-networks in which the proteins are topologically and functionally correlated. The algorithm can deal even with sparse interaction networks by analysing the topology of nodes adjacent to the proteins directly interacting with the current solution. AlignMCL^14^ is an extended version of AlignNemo. AlignMCL takes as input two single graph, ad it merges them in a *alignment graph*. Then, AlignMCL mines the alignment graph by applying the Markov cluster algorithm (MCL)^16^. AlignMCL extracts sub-networks that are functionally correlated without the imposition of any particular topology (see^31^ for complete details about the construction of the alignment graph). GLAlign (Global Local Aligner)^32^, is a new local network alignment methodology. GLAlign exploits a node mapping produced by a global aligner to guide the local alignment building. In detail, GLAlign mixes topology information from global alignment and biological information according to a linear combination schema, and it uses the combination information for the building of local alignment. LocalAli^33^ is a local aligner that exploits maximum-parsimony evolutionary model to construct a local alignment represented as conserved modules.

Global Network Alignment (GNA) algorithms aim to discover a one to one mapping among nodes of the initial networks. The literature contains several algorithms, and here we recall only the most popular approaches (^34^). Traditional GNAs employ a two-stage procedure. During the first stage, they apply a cost function to estimate pairwise similarities among nodes. Then, they use an alignment method to quickly determinate, among all probable alignments, the one with a high score in relation to the overall similarity on all aligned node. GNAs may be classified by their alignment strategy on (a) seed-extend and (b) search. Both aim to maximise the total node similarity (or node conservation) or the number of conserved edges (edge conservation)^12^. Examples of methods belonging to the first class are: IsoRank^35^, GRAAL^36^ and the GRAAL family (H-GRAAL^37^, MI-GRAAL^38^, C-GRAAL^39^, L-GRAAL^40^), and GHOST^41,41,42^. WAVE^43^ builds the alignment by applying a *seed-and-extend* alignment method that optimizes node and edge conservation. IGLOO^44^ is a novel strategy that combines global network alignment and local network alignment algorithms to build a functionally and topologically qualitative alignment. MAGNA^45^ is a graphlet based global network aligner based on a search strategy that applies a genetic methodology to improve the alignment building. MAGNA simulates a set of alignments and then it selects the best one. MAGNA++^46^ extends MAGNA maximizing both edge and node conservation measures.

Another prominent NA algorithm based on a search strategy is Simulated Annealing Network Aligner (SANA)^47^. SANA receives the initial networks and an alignment built with a different aligner o in random way and applies a simulated annealing to construct a global alignment. UAlign^48^ assembles global alignments produced by diverse network algorithms with the aim to overcome the restriction of global network alignments.

Previously introduced NA algorithms deal only with homogeneous networks. More recently, Gu *et al*.^15^ proposed a recent approach of alignment of heterogeneous networks by formalising a framework that extends three homogeneous NA methods, WAVE, MAGNA++, and SANA, to allow for heterogeneous NA. The main contribution of this method is the formulation of heterogeneous (or coloured) graphlets. These graphlets are then used to build the alignment as a measure of node-similarity. This approach builds a global alignment, while L-HetNetAligner produces a local one. Currently, the interest for algorithms dealing with heterogeneous network data is growing in the social network analysis area; see^49^ for an extensive survey.

#### Heterogeneous Networks in Biology and Medicine

Initially, the use of heterogeneous networks has been explored for data integration. Przytycka *et al*.,^50^, explored the integration of different types of molecules (genes, proteins and transcription factors) and their various kinds of interactions into a heterogeneous network. Mitra *et al*.,^51^ discussed a lot of these approaches in a review, and the recent study by Cowen *et al*.,^52^ summarises all these approaches. The STRING database^53^ uses heterogeneous networks to model functional associations among genes. Other approaches use heterogeneous networks to early detect and to monitor the progression of diseases^52,54–56^.

Special cases of heterogeneous networks are multilayer networks (that use different edge types between the same nodes) or dual networks. For instance, Wu *et al*.^57^ use a dual network model of protein interactions to explain genetic interactions. A dual network model uses a pair of networks; one network depicts physical interactions between proteins, and the other one represents genetic interactions. Other approaches try to represent the dynamic aspects of a network (i.e. changes of the network structure over time) using ad hoc defined temporal networks^58^. Another interesting approach is the use of multimodal networks^59^. A multimodal network is composed by a set of nodes connected by different sets of edges. More recently, some novel algorithms have been introduced to mine heterogeneous networks. For example, Li *et al*. propose a Pagerank based algorithm to reveal modules in heterogeneous biological networks^60^. Reimand *et al*. propose a new framework for biological heterogeneous network analysis and module discovery, and provide a public web server for use by domain scientists^61^.

#### Quality Evaluation of Network Alignmnents

The evaluation of the quality of a network alignment algorithms is usually made by supposing the knowledge of the true node and edge mapping. One of the most popular measures is node correctness (NC)^36^. Given two networks ${N}_{1}$, and ${N}_{2}$ and an alignment $f$ that maps nodes from ${N}_{1}$ to ${N}_{2}$. NC is defined as the set of nodes of one network mapped to nodes of the other networks compared to the true node mapping. NC is not used for local network alignments since some local network alignment algorithms may map a node from a network with many nodes of the other network^21^. Consequently, Meng *et al*., defined three novel measures *P-NC*, *R-NC*, and *F-NC* that may be used for both global and local alignments. Let suppose the alignment $f$ produce a set of node pairs composed by ${N}_{al}$ nodes while the true node mapping is composed by ${M}_{tr}$ nodes. *P-NC* is calculated as as $\frac{{M}_{tr}\ \cap \ {N}_{al}}{{M}_{tr}}$. *R-NC* is defined as $\frac{{M}_{tr}\ \cap \ {N}_{al}}{{N}_{al}}$. *F-NC*, is a combination of the two previous measures. In parallel we also compute the fraction of edges that are fine preserved in a alignment by taking into account the true edge mapping.

Similarly, to compute the fraction of edges are correctly mapped in an alignment, 3 popular measures have been proposed: edge correctness (EC)^36^, induced conserved structure (ICS)^41^, and symmetric substructure score (S3)^45^ that outperforms the previous ones. Similarly to node correctness, the S3 cannot be used directly to evaluate the quality of local network alignment algorithms. Therefore other measures have been defined^21^ such as generalised S3 (GS3) and high node coverage S3 (NCV-S3).

#### Applications

In addition to the local alignment of heterogeneous networks, other applications of L-HetNetAligner include the capability to infer missing edge, also known as link prediction^62^, and the detection of communities^63^ from the alignment graph.

The goal of **Link Prediction**^62^ is to discover missing links. In case of a missing link, link prediction ranks the best candidates of the node pairs for this missing link based on the attributes that contain information about the nodes, edges or the entire graph. Thus, link prediction aims to discover missing data in a network or to de-noise a network.

**Detection of conserved communities** concerns the identification of substructure with an arbitrary topology that are conserved in both input networks. The communities are groups of nodes which are more densely connected than with the rest of the networks. The identification of communities in graph enables knowledge extraction from the aligned network.

## Conclusion

L-HetNetAligner is a novel algorithm for local alignment of heterogeneous networks used for modelling biological systems, such as living cells, composed by a broad set of different objects mutually interacting. Nowadays, many different high throughput platforms have caused the availability of data about such objects. L-HetNetAligner takes as input two heterogeneous networks (node-coloured graphs) and a list of paired nodes (one for each network) used as seed and builds a local alignment of them.

Our algorithm, starting from an inital list of seed nodes, builds an auxiliary structure called heterogeneous alignment graph in which each node correspond to a pair of nodes of the input networks selected based on the input list and in which each edge is calculated and weighted by analysing the input networks. Then communities are extracted from this graph. Each community corresponds to a single region of local similarity. The community extraction has been performed using an existing algorithm for clustering of networks: MCL.

Since that there are not gold standards for evaluating the quality of local aligners, we designed a set of experiments following existing literature to demonstrate: (i) the need for the introduction of an ad hoc algorithm for heterogeneous networks, (ii) the good performances of L-HetNetAligner for both synthetic and real heterogeneous networks.

Our results confirmed initially that the use of an ad hoc algorithm for the alignment of heterogeneous networks outperform classical algorithms when they are forced to analyse heterogeneous networks as evidenced in Section Comparison to Homogeneous Aligners (see Table 3 and 4). These tables show that homogeneus aligners fail to produce alignment with less quality. We compared L-HetNetAligner to AlignNemo and Align-MCL on synthetic networks with two colours. We forced AlignMCL and AlignNemo to build the alignment of heterogeneous networks using 12 synthetic networks. Table 3 reports the performances of our algorithm in terms of precision on nodes while Table 4 reports the quality considering both nodes and edges.

Our algorithm showed good performances both on synthetic and real networks. We aligned synthetic generated networks with different models to test the performances of our algorithm on different network structure . This experiment aims to prove the robustness of our approach to the change of network structure. As indicated in Section Syntethic Networks: Topological Evaluation, our algorithms showed good performances in terms of topological quality of obtained alignments.

The use of colours also improves the robustness to noise compared to the use of fewer colours. Fig. 6 clearly shows that for a given level of noise the use of colours improves the quality of the alignment. Besides, the robustness to the impact of noise is better. This trend is evident considering both NCV-GS3 as well as node F-NC as reported in Fig. 7 and in Supplementary File 1 that contains more data in Tables 1, 2, 3, 4, 5, 6 in Supplementary File 1). Moreover, as shown in Tables 49, 50, 51, 52, 53, 54, 55, 56 of the Supplementary File 1, these performances are maintained when changing network structure and dimensions. L-HetAligner is also robust when noise is added to the networks. We considered both adding and removing edges and nodes, and in each of these four cases, the algorithm realised good alignments (see Tables 13, 14, 15, 16, 17, 18, 25, 26, 27, 28, 29, 30, 37, 38, 39, 40, 41, 42 in Supplementary File 1).

We also tested L-HetNetAligner on a real network: the heterogeneous network extracted from HetioNet Database. We here performed both topological and functional evaluation of results. Functional evaluation was performed by assessing the biological relevance of aligned subnetworks. In general, groups of related entities should have a similar biological role or share some functions. To test the relatedness of a group of biological entities, we used Semantic Similarity measures . The aligned regions showed a relatedness significantly higher than by chance.

We also considered different versions of Hetionet network; therefore we were able to analyse networks with an increasing number of colours, from 2 to 4. Results evidenced that the use of more colours resulted in the production of results with higher functional quality (see Figs. 8, 9 and Tables 7, 8, 9, 10, 11, 12, 19, 20, 21, 22, 23, 24, 31, 32, 33, 34, 35, 36, 43, 44, 45, 46, 47, 48 in Supplementary File 1). Results also demonstrate that modules extracted by aligning the Hetionet network with more node colours are better in terms of semantic similarity than those extracted from aligning the Hetionet networks with a single node colour (see Fig. 10). Therefore, these results confirm both the need for the use of heterogeneous networks and the introduction of novel algorithms designed for this context. Our algorithm is also able to predict new knowledge in terms of missing links from a network to another one. For these aim, we consider first the synthetic networks described before and the set of initial node pairs used as seed. Then we removed an increasing fraction of edges randomly into the second network (from 5% to 25% of edges), and we aligned these networks. A missing link is revealed as a mismatch or a gap. Results showed a significant number of missing links that have been revealed (see Table 61 in Supplementary File 1). We repeated this experiment also for Hetionet dataset, and our algorithm was able to predict the missing links even in this case (see Table 62 in Supplementary File 1). As future work, we plan to investigate the following challenges: (i) the introduction of a framework that can suggest optimal parameters based on input networks (i.e. topology), and on the problem (i.e. search for the conserved region, prediction of missing links); (ii) the realisation of a customised version of L-HetNetAligner for best performances in missing link prediction; (iii) the application on social network datasets; (iv) the extraction of overlapping regions; (v) the use of high-performance infrastructure to reduce execution time.

## Supplementary information

Supplementary Information.

## Data Availability

The website https://sites.google.com/view/heterogeneusnetworkalignment/home  contains Supplementary File 1 materials and the source code.
