# Supplementary material for: L-HetNetAligner: A novel algorithm for Local Alignment of Heterogeneous Biological Networks
Source: Sci Rep. 2020 Mar 3;10:3901. doi: 10.1038/s41598-020-60737-5 (PMC7054427; doi:10.1038/s41598-020-60737-5)
Supplement: Supplementary file 1 — Supplementary Information. [file 41598_2020_60737_MOESM1_ESM.pdf]

L-HetNetAligner: A novel algorithm for Local Alignment of Heterogeneous Biological Networks.

Marianna Milano[1], Tijana Milenković[2], Mario Cannataro[1,3], and Pietro Hiram Guzzi[1,3,*]

[1]University of Catanzaro, Department of Surgical and Medical Sciences, Catanzaro, 88040, Italy
[2]University of Notre Dame, Department of Computer Science and Engineering, Notre Dame, Indiana
[3]Data Analytics Research Center, University of Catanzaro
[*]Corresponding Author: hguzzi@unicz.it

ABSTRACT

Supplementary File 1.

Appendix 1

List of Figures

List of Tables

1 Table shows the NCV-GS^3 scores obtained by aligning the first synthetic network with its noisy versions (Altered Networks). All synthetic networks are considered. The table shows significant improvements in the values of NCV-GS^3 and hence in the quality of the alignments when considering networks with many colours with respect to the network with a single colour. The improvement is also stable for all the networks. 6
2 Table shows the GS^3 scores obtained by aligning the first synthetic network with its noisy versions (Altered Networks). All the results are equal to 1, therefore the table shows that the introduction of colors does not cause a worsening of the results. 7
3 Table shows the NCV scores obtained by aligning the first synthetic network with its noisy versions (Altered Networks). All synthetic networks are considered. The table shows significant improvements in the values of NCV and hence in the quality of the alignments when considering networks with many colours with respect to the network with a single colour. The improvement is also stable for all the networks. 8
4 Table shows the P-NC scores obtained by aligning the first synthetic network with its noisy versions (Altered Networks). Table shows that the alignment quality is constant when adding colors to the networks. 9
5 Table shows the R-NC scores obtained by aligning the first synthetic network with its noisy versions (Altered Networks). All synthetic networks are considered. The table shows significant improvements in the values of R-NC and hence in the quality of the alignments when considering networks with many colours with respect to the network with a single colour. The improvement is also stable for all the networks. 10
6 Table shows the F-NC scores obtained by aligning the first synthetic network with its noisy versions (Altered Networks). All synthetic networks are considered. The table shows significant improvements in the values of F-NC and hence in the quality of the alignments when considering networks with many colours with respect to the network with a single colour. The improvement is also stable for all the networks. 11
7 NCV-GS^3 scores obtained by aligning the original Hetionet network with its noisy versions. Table shows a remarkable trend of improvement when considering networks with many colours. Results are similar for all the networks. 12
8 Table shows the GS^3 scores obtained by aligning the original Hetionet network with its noisy versions. Table shows that the alignment quality is constant when considering colours. 12
9 Table shows the NCV scores obtained by aligning the original Hetionet network with its noisy versions. Table shows a remarkable trend of improvement when considering networks with many colours for all the networks. . . . 12
10 Table shows the P-NC scores obtained by aligning the original Hetionet network with its noisy versions. Table shows that the alignment quality is constant when considering colours. 12

11 Table shows the R-NC scores obtained by aligning the original Hetionet network with its noisy versions. Table shows a remarkable trend of improvement when considering networks with many colours. 12
12 Table shows the F-NC scores obtained by aligning the original Hetionet network with its noisy versions. Table shows a remarkable trend of improvement when considering networks with many colours for all the networks. . 12
13 Table shows the NCV-GS^3 scores obtained by aligning the original synthetic network with its noisy versions (adding edges) for all the networks. Table shows a significant improvement of the quality of the alignments when considering networks with many colours. The improvement is also stable for all the networks. 13
14 Table shows GS^3 scores obtained by aligning the original synthetic network with its noisy versions (adding edges) for all the networks. Table shows that the alignment quality does not get worse when considering colors. . 14
15 Table shows the NCV scores obtained by aligning the original synthetic network with its noisy versions for all the networks. The altered networks are generated by randomly adding edges. Table shows a significant improvement of the quality of the alignments when considering networks with many colours. The improvement is also stable for all the networks. 16
16 P-NC scores obtained by aligning the original synthetic network with its noisy versions (adding edges) for all the networks. Table shows that the alignment quality is constant by considering networks with many colours. . . 17
17 Table shows the R-NCscores obtained by aligning the original synthetic network with its noisy versions for all the networks. The altered networks are generated by randomly adding edges. Table shows a significant improvement of the quality of the alignments when considering networks with many colours. The improvement is also stable for all the networks. 18
18 Table shows the F-NC scores obtained by aligning the original synthetic network with its noisy versions for all the networks. The altered networks are generated by randomly adding edges. Table shows a significant improvement of the quality of the alignments when considering networks with many colours. The improvement is also stable for all the networks. 19
19 Table summarises NCV-GS^3 scores obtained by aligning the original Hetionet network with its noisy versions obtained by adding edges to the first one. Table shows a remarkable trend of improvement when considering networks with many colours. 20
20 Table summarises GS^3 scores obtained by aligning the original Hetionet network with its noisy versions. Altered networks are obtained by adding edges randomly. Table shows that the alignment quality is constant when considering networks with many colours. 20
21 Table summarises the NCV scores obtained by aligning the original Hetionet network with its noisy versions. Altered networks presents more random edges. Table shows a remarkable trend of improvement when considering networks with many colours for all the networks. 20
22 Table summarises P-NC scores obtained by aligning the original Hetionet network with its noisy versions. Table shows that the alignment quality is constant when considering colours. 20
23 Table summarises the R-NC scores obtained by aligning the original Hetionet network with its noisy versions when adding edges. Table shows a remarkable trend of improvement when considering networks with many colours. 20
24 Table summarises the F-NC scores obtained by aligning the original Hetionet network with its noisy versions when adding edges. Table shows a remarkable trend of improvement when considering networks with many colours. 21
25 The table shows the NCV-GS^3 scores obtained by aligning the original synthetic network with its noisy versions when adding nodes for all the networks. Table shows a significant improvement of the quality of the alignments when considering networks with many colours. The improvement is also stable for all the networks. 22
26 Table shows the GS^3 scores obtained by aligning the original synthetic network with its noisy versions when adding nodes for all the networks. Table shows that the alignment quality is constant. 23
27 The table shows the NCV scores obtained by aligning the original synthetic network with its noisy versions when adding nodes for all the networks. Table shows a significant improvement of the quality of the alignments when considering networks with many colours. The improvement is also stable for all the networks. 24
28 P-NC scores obtained by aligning the original synthetic network with its noisy versions for all the networks. Networks are obtained by adding nodes. Table shows that the alignment quality is constant. 25
29 The table shows the R-NC scores obtained by aligning the original synthetic network with its noisy versions when adding nodes for all the networks. Table shows a significant improvement of the quality of the alignments when considering networks with many colours. The improvement is also stable for all the networks. 26

30 The table shows the F-NC scores obtained by aligning the original synthetic network with its noisy versions when adding nodes for all the networks. Table shows a significant improvement of the quality of the alignments when considering networks with many colours. The improvement is also stable for all the networks. 27
31 Table summarises the NCV-GS^3 scores obtained by aligning the original Hetionet network with its noisy versions. Altered networks are obtained by adding nodes randomly. Table shows a remarkable trend of improvements when considering networks with many colours for all the networks. 28
32 Table summarises the GS^3 scores obtained by aligning the original Hetionet network with its noisy versions when adding nodes. Table shows that the alignment quality is constant. 28
33 The table shows the NCV scores obtained by aligning the original synthetic network with its noisy versions when adding nodes for all the networks. Table shows a significant improvement of the quality of the alignments when considering networks with many colours. The improvement is also stable for all the networks. 29
34 Table shows the P-NC scores obtained by aligning the original Hetionet network with its noisy versions. Altered networks presents more nodes. Table shows that the alignment quality is constant. 29
35 The table shows the R-N scores obtained by aligning the original synthetic network with its noisy versions when adding nodes for all the networks. Table shows a significant improvement of the quality of the alignments when considering networks with many colours. The improvement is also stable for all the networks. 30
36 The table shows the F-NC scores obtained by aligning the original synthetic network with its noisy versions when adding nodes for all the networks. Table shows a significant improvement of the quality of the alignments when considering networks with many colours. The improvement is also stable for all the networks. 30
37 Table summarises the NCV-GS^3 scores obtained by aligning the original synthetic network with its noisy versions obtained by removing nodes. Table shows a significant improvement of the quality of the alignments when considering networks with many colours. The improvement is also stable for all the considered networks. . 32
38 Table shows GS^3 scores obtained by aligning the original synthetic network with its noisy versions for all the networks. Table shows that the alignment quality is constant. 33
39 Table summarises the NCV scores obtained by aligning the original synthetic network with its noisy versions obtained by removing nodes. Table shows a significant improvement of the quality of the alignments when considering networks with many colours. The improvement is also stable for all the considered networks. 35
40 P-NC scores obtained by aligning the original synthetic network with its noisy versions for all the networks. Table shows that the alignment quality is constant. 36
41 Table summarises the R-NC scores obtained by aligning the original synthetic network with its noisy versions obtained by removing nodes. Table shows a significant improvement of the quality of the alignments when considering networks with many colours. The improvement is also stable for all the considered networks. 37
42 Table summarises the F-NC scores obtained by aligning the original synthetic network with its noisy versions obtained by removing nodes. Table shows a significant improvement of the quality of the alignments when considering networks with many colours. The improvement is also stable for all the considered networks.. 38
43 NCV-GS^3 scores obtained by aligning the original Hetionet network with its noisy versions (removing nodes). Table shows a remarkable trend of improvements when considering networks with many colours for all the networks. 39
44 GS^3 scores obtained by aligning the original Hetionet network with its noisy versions (removing nodes). Table shows that the alignment quality is constant when considering networks with many colours. 39
45 Table shows the NCV scores obtained by aligning the original Hetionet network with its noisy versions when removing nodes. Table shows a remarkable trend of improvement when considering networks with many colours for all the networks. 39
46 Table shows the P-NC scores obtained by aligning the original Hetionet network with its noisy versions (when removing nodes). Table shows that the alignment quality is constant by considering both un-coloured and more colours networks. 39
47 Table shows the R-NC scores obtained by aligning the original Hetionet network with its noisy versions when removing nodes. Table shows a remarkable trend of improvements when considering networks with many colours for all the networks. 39
48 Table shows the F-NC scores obtained by aligning the original Hetionet network with its noisy versions when removing nodes. Table shows a remarkable trend of improvements when considering networks with many colours for all the networks. 40
49 Table shows the NCV-GS^3 scores obtained by aligning the original synthetic network with its noisy versions when removing edges for all the network models having a single colour. 40

50 Table shows the NCV-GS^3 scores obtained by aligning the original synthetic network with its noisy versions. Table presents two colored networks for all the network models. 41
51 Table shows the NCV-GS^3 scores obtained by aligning the original synthetic network with three colors with its noisy versions when removing edges for all the network models. 41
52 Table summarises the NCV-GS^3 scores obtained by aligning the original synthetic network with four colours with its noisy versions when removing edges for all the network models. 42
53 Table shows the F-NC scores obtained by aligning the original synthetic network with one color with four colours with its noisy versions when removing edges for all the network models. 42
54 Table shows the F-NC scores obtained by aligning the original synthetic network with two colors and its noisy versions for all the network models. 43
55 Table shows F-NC scores obtained by aligning the original synthetic network with three colours and its noisy versions when removing edges for all the network models. 44
56 Table shows the F-NC scores obtained by aligning the original synthetic network with four colours and its noisy versions when removing edges for all the network models. 45
57 NCV-GS^3 scores obtained by aligning the original network with its noisy versions (edges removing). Table shows a remarkable trend of improvements when considering networks with many colours for all the networks. . 46
58 F-NC scores obtained by aligning the original network with its noisy versions (edges removing). Table shows a remarkable trend of improvements when considering networks with many colours for all the networks. 47
59 NCV-GS^3 and F-NC scores obtained by aligning the synthetic network with its noisy versions and the four subnetworks with their noisy versions. 48
60 NCV-GS^3 and F-NC scores obtained by aligning the Hetionet network with its noisy versions and the four subnetworks with their noisy versions 49
61 Corrected predicted links obtained by aligning the original synthetic network and four subnetworks with its noisy versions for all the networks. 50
62 Corrected predicted links obtained by aligning the original Hetionet network and four subnetworks with its noisy versions for all the networks. 50
63 NCV-GS^3 scores obtained by aligning the original synthetic network with its noisy versions for all the networks. In the alignment graph building all pair of nodes were selected. 51
64 F-NC scores obtained by aligning the original synthetic network with its noisy versions for all the networks. In the alignment graph building all pair of nodes were selected. 52
65 F-NC scores obtained by aligning the original synthetic network with its noisy versions for all the networks. In the alignment graph building all pair of nodes were selected. 53
66 F-NC scores obtained by aligning the original synthetic network with its noisy versions for all the networks. In the alignment graph building all pair of nodes were selected. 54

Quality of the alignments for synthetic networks

Tables 1, 2, 3, 4, 5, and 6 report the NCV-GS^3, GS^3, NCV, P-NC, R-NC, F-NC measures computed on each module for the four versions of synthetic heterogeneous networks.

Quality of the alignments for the Hetionet Network

Table 7, Table 8, Table 9, Table 10, Table 11, Table 12 report the NCV-GS^3, GS^3, NCV, P-NC, R-NC, F-NC measures computed on each module for the four versions of the Hetionet network.

Quality of the Alignments for synthetic heterogeneous networks with noise.

Table 13, Table 14, Table 15, Table 16, Table 17, Table 18 report the NCV-GS^3, GS^3, NCV, P-NC, R-NC, F-NC measures computed on each module for the four versions of synthetic heterogeneous networks obtained by aligning each network with its noisy versions built by adding a percentage of edges.

Table 19, Table 20, Table 21, Table 22, Table 23, Table 24 report the NCV-GS^3, GS^3, NCV, P-NC, R-NC, F-NC measures computed on each module for the four versions of the Hetionet network obtained by aligning each network with its noisy version built by adding a percentage of edges.

Table 1. Table shows the NCV-GS^3 scores obtained by aligning the first synthetic network with its noisy versions (Altered Networks). All synthetic networks are considered. The table shows significant improvements in the values of NCV-GS^3 and hence in the quality of the alignments when considering networks with many colours with respect to the network with a single colour. The improvement is also stable for all the networks.

Network	Altered Networks	NCV-GS^3 on 1 coloured version	NCV-GS^3 on 2 coloured version	NCV-GS^3 on 3 coloured version	NCV-GS^3 on 4 coloured version
N1	0	0.535	0.811	0.889	0.99
	5	0.533	0.799	0.878	0.989
	10	0.531	0.792	0.874	0.983
	15	0.523	0.766	0.872	0.978
	20	0.52	0.763	0.85	0.96
	25	0.514	0.759	0.832	0.957
N2	0	0.529	0.81	0.883	0.997
	5	0.528	0.809	0.883	0.988
	10	0.524	0.802	0.883	0.986
	15	0.521	0.8	0.871	0.979
	20	0.516	0.785	0.865	0.976
	25	0.514	0.773	0.843	0.953
N3	0	0.535	0.811	0.863	0.983
	5	0.533	0.799	0.848	0.99
	10	0.531	0.766	0.843	0.989
	15	0.523	0.763	0.833	0.978
	20	0.52	0.759	0.832	0.96
	25	0.514	0.752	0.83	0.957
N4	0	0.532	0.797	0.863	0.971
	5	0.53	0.797	0.862	0.967
	10	0.525	0.783	0.857	0.963
	15	0.521	0.782	0.856	0.96
	20	0.517	0.782	0.833	0.952
	25	0.514	0.775	0.83	0.952
N5	0	0.533	0.797	0.878	0.993
	5	0.53	0.789	0.868	0.987
	10	0.53	0.777	0.867	0.986
	15	0.526	0.775	0.864	0.982
	20	0.519	0.773	0.851	0.981
	25	0.518	0.758	0.838	0.958
N6	0	0.533	0.806	0.868	0.992
	5	0.525	0.805	0.862	0.988
	10	0.521	0.782	0.857	0.977
	15	0.519	0.781	0.852	0.975
	20	0.516	0.77	0.849	0.966
	25	0.514	0.765	0.832	0.952
N7	0	0.534	0.811	0.884	0.992
	5	0.531	0.807	0.865	0.982
	10	0.526	0.792	0.864	0.981
	15	0.521	0.761	0.856	0.978
	20	0.518	0.76	0.851	0.968
	25	0.516	0.755	0.844	0.959
N8	0	0.53	0.792	0.886	0.998
	5	0.525	0.787	0.876	0.988
	10	0.524	0.772	0.866	0.981
	15	0.523	0.772	0.853	0.977
	20	0.522	0.766	0.84	0.974
	25	0.514	0.76	0.838	0.972
N9	0	0.529	0.795	0.868	0.998
	5	0.528	0.784	0.866	0.991
	10	0.526	0.772	0.861	0.973
	15	0.524	0.761	0.857	0.969
	20	0.519	0.758	0.853	0.968
	25	0.516	0.754	0.838	0.956
N10	0	0.533	0.81	0.869	1
	5	0.529	0.808	0.868	0.976
	10	0.528	0.808	0.865	0.961
	15	0.528	0.771	0.861	0.959
	20	0.522	0.767	0.852	0.954
	25	0.515	0.757	0.85	0.953
N11	0	0.533	0.81	0.882	1
	5	0.531	0.809	0.881	0.996
	10	0.527	0.797	0.879	0.986
	15	0.525	0.793	0.871	0.982
	20	0.521	0.775	0.868	0.98
	25	0.518	0.773	0.848	0.963
N12	0	0.527	0.809	0.887	0.99
	5	0.523	0.787	0.885	0.977
	10	0.518	0.786	0.884	0.969
	15	0.516	0.78	0.859	0.969
	20	0.513	0.768	0.842	0.966
	25	0.513	0.756	0.837	0.959

Table 2. Table shows the GS^3 scores obtained by aligning the first synthetic network with its noisy versions (Altered Networks). All the results are equal to 1, therefore the table shows that the introduction of colors does not cause a worsening of the results.

Network	Altered Networks	GS^3 on 1 coloured version	GS^3 on 2 coloured version	GS^3 on 3 coloured version	GS^3 on 4 coloured version
N1	0	1	1	1	1
	5	1	1	1	1
	10	1	1	1	1
	15	1	1	1	1
	20	1	1	1	1
	25	1	1	1	1
N2	0	1	1	1	1
	5	1	1	1	1
	10	1	1	1	1
	15	1	1	1	1
	20	1	1	1	1
	25	1	1	1	1
N3	0	1	1	1	1
	5	1	1	1	1
	10	1	1	1	1
	15	1	1	1	1
	20	1	1	1	1
	25	1	1	1	1
N4	0	1	1	1	1
	5	1	1	1	1
	10	1	1	1	1
	15	1	1	1	1
	20	1	1	1	1
	25	1	1	1	1
N5	0	1	1	1	1
	5	1	1	1	1
	10	1	1	1	1
	15	1	1	1	1
	20	1	1	1	1
	25	1	1	1	1
N6	0	1	1	1	1
	5	1	1	1	1
	10	1	1	1	1
	15	1	1	1	1
	20	1	1	1	1
	25	1	1	1	1
N7	0	1	1	1	1
	5	1	1	1	1
	10	1	1	1	1
	15	1	1	1	1
	20	1	1	1	1
	25	1	1	1	1
N8	0	1	1	1	1
	5	1	1	1	1
	10	1	1	1	1
	15	1	1	1	1
	20	1	1	1	1
	25	1	1	1	1
N9	0	1	1	1	1
	5	1	1	1	1
	10	1	1	1	1
	15	1	1	1	1
	20	1	1	1	1
	25	1	1	1	1
N10	0	1	1	1	1
	5	1	1	1	1
	10	1	1	1	1
	15	1	1	1	1
	20	1	1	1	1
	25	1	1	1	1
N11	0	1	1	1	1
	5	1	1	1	1
	10	1	1	1	1
	15	1	1	1	1
	20	1	1	1	1
	25	1	1	1	1
N12	0	1	1	1	1
	5	1	1	1	1
	10	1	1	1	1
	15	1	1	1	1
	20	1	1	1	1
	25	1	1	1	1

Table 3. Table shows the NCV scores obtained by aligning the first synthetic network with its noisy versions (Altered Networks). All synthetic networks are considered. The table shows significant improvements in the values of NCV and hence in the quality of the alignments when considering networks with many colours with respect to the network with a single colour. The improvement is also stable for all the networks.

Network	Altered Networks	NCV on 1 coloured version	NCV on 2 coloured version	NCV on 3 coloured version	NCV on 4 coloured version
N1	0	0.286	0.658	0.790	0.980
	5	0.284	0.638	0.771	0.978
	10	0.282	0.627	0.764	0.966
	15	0.274	0.587	0.760	0.956
	20	0.270	0.582	0.723	0.922
	25	0.264	0.576	0.692	0.916
N2	0	0.280	0.656	0.780	0.994
	5	0.279	0.654	0.780	0.976
	10	0.275	0.643	0.780	0.972
	15	0.271	0.640	0.759	0.958
	20	0.266	0.616	0.748	0.953
	25	0.264	0.598	0.711	0.908
N3	0	0.286	0.658	0.745	0.966
	5	0.284	0.638	0.719	0.980
	10	0.282	0.587	0.711	0.978
	15	0.274	0.582	0.694	0.956
	20	0.270	0.576	0.692	0.922
	25	0.264	0.566	0.689	0.916
N4	0	0.283	0.635	0.745	0.943
	5	0.281	0.635	0.743	0.935
	10	0.276	0.613	0.734	0.927
	15	0.271	0.612	0.733	0.922
	20	0.267	0.612	0.694	0.906
	25	0.264	0.601	0.689	0.906
N5	0	0.284	0.635	0.771	0.986
	5	0.281	0.623	0.753	0.974
	10	0.281	0.604	0.752	0.972
	15	0.277	0.601	0.746	0.964
	20	0.269	0.598	0.724	0.962
	25	0.268	0.575	0.702	0.918
N6	0	0.284	0.650	0.753	0.984
	5	0.276	0.648	0.743	0.976
	10	0.271	0.612	0.734	0.955
	15	0.269	0.610	0.726	0.951
	20	0.266	0.593	0.721	0.933
	25	0.264	0.585	0.692	0.906
N7	0	0.285	0.658	0.781	0.984
	5	0.282	0.651	0.748	0.964
	10	0.277	0.627	0.746	0.962
	15	0.271	0.579	0.733	0.956
	20	0.268	0.578	0.724	0.937
	25	0.266	0.570	0.712	0.920
N8	0	0.281	0.627	0.785	0.996
	5	0.276	0.619	0.767	0.976
	10	0.275	0.596	0.750	0.962
	15	0.274	0.596	0.728	0.955
	20	0.272	0.587	0.706	0.949
	25	0.264	0.578	0.702	0.945
N9	0	0.280	0.632	0.753	0.996
	5	0.279	0.615	0.750	0.982
	10	0.277	0.596	0.741	0.947
	15	0.275	0.579	0.734	0.939
	20	0.269	0.575	0.728	0.937
	25	0.266	0.569	0.702	0.914
N10	0	0.284	0.656	0.755	1
	5	0.280	0.653	0.753	0.953
	10	0.279	0.653	0.748	0.924
	15	0.279	0.594	0.741	0.920
	20	0.272	0.588	0.726	0.910
	25	0.265	0.573	0.723	0.908
N11	0	0.284	0.656	0.778	1
	5	0.282	0.654	0.776	0.992
	10	0.278	0.635	0.773	0.972
	15	0.276	0.629	0.759	0.964
	20	0.271	0.601	0.753	0.960
	25	0.268	0.598	0.719	0.927
N12	0	0.278	0.654	0.787	0.980
	5	0.274	0.619	0.783	0.955
	10	0.268	0.618	0.781	0.939
	15	0.266	0.608	0.738	0.939
	20	0.263	0.590	0.709	0.933
	25	0.263	0.572	0.701	0.920

Table 4. Table shows the P-NC scores obtained by aligning the first synthetic network with its noisy versions (Altered Networks). Table shows that the alignment quality is constant when adding colors to the networks.

Network	Altered Networks	P-NC on 1 coloured version	P-NC on 2 coloured version	P-NC on 3 coloured version	P-NC on 4 coloured version
N1	0	1	1	1	1
	5	1	1	1	1
	10	1	1	1	1
	15	1	1	1	1
	20	1	1	1	1
	25	1	1	1	1
N2	0	1	1	1	1
	5	1	1	1	1
	10	1	1	1	1
	15	1	1	1	1
	20	1	1	1	1
	25	1	1	1	1
N3	0	1	1	1	1
	5	1	1	1	1
	10	1	1	1	1
	15	1	1	1	1
	20	1	1	1	1
	25	1	1	1	1
N4	0	1	1	1	1
	5	1	1	1	1
	10	1	1	1	1
	15	1	1	1	1
	20	1	1	1	1
	25	1	1	1	1
N5	0	1	1	1	1
	5	1	1	1	1
	10	1	1	1	1
	15	1	1	1	1
	20	1	1	1	1
	25	1	1	1	1
N6	0	1	1	1	1
	5	1	1	1	1
	10	1	1	1	1
	15	1	1	1	1
	20	1	1	1	1
	25	1	1	1	1
N7	0	1	1	1	1
	5	1	1	1	1
	10	1	1	1	1
	15	1	1	1	1
	20	1	1	1	1
	25	1	1	1	1
N8	0	1	1	1	1
	5	1	1	1	1
	10	1	1	1	1
	15	1	1	1	1
	20	1	1	1	1
	25	1	1	1	1
N9	0	1	1	1	1
	5	1	1	1	1
	10	1	1	1	1
	15	1	1	1	1
	20	1	1	1	1
	25	1	1	1	1
N10	0	1	1	1	1
	5	1	1	1	1
	10	1	1	1	1
	15	1	1	1	1
	20	1	1	1	1
	25	1	1	1	1
N11	0	1	1	1	1
	5	1	1	1	1
	10	1	1	1	1
	15	1	1	1	1
	20	1	1	1	1
	25	1	1	1	1
N12	0	1	1	1	1
	5	1	1	1	1
	10	1	1	1	1
	15	1	1	1	1
	20	1	1	1	1
	25	1	1	1	1

Table 5. Table shows the R-NC scores obtained by aligning the first synthetic network with its noisy versions (Altered Networks). All synthetic networks are considered. The table shows significant improvements in the values of R-NC and hence in the quality of the alignments when considering networks with many colours with respect to the network with a single colour. The improvement is also stable for all the networks.

Network	Altered Networks	R-NC on 1 coloured version	R-NC on 2 coloured version	R-NC on 3 coloured version	R-NC on 4 coloured version
N1	0	0.173	0.498	0.707	0.960
	5	0.172	0.496	0.697	0.929
	10	0.171	0.489	0.637	0.916
	15	0.169	0.487	0.613	0.908
	20	0.163	0.483	0.581	0.889
	25	0.158	0.483	0.578	0.817
N2	0	0.171	0.511	0.707	0.980
	5	0.161	0.511	0.697	0.964
	10	0.159	0.510	0.621	0.953
	15	0.158	0.504	0.615	0.870
	20	0.158	0.500	0.582	0.845
	25	0.154	0.489	0.579	0.830
N3	0	0.166	0.498	0.711	0.922
	5	0.166	0.489	0.699	0.897
	10	0.163	0.496	0.621	0.882
	15	0.162	0.487	0.615	0.859
	20	0.156	0.483	0.582	0.859
	25	0.156	0.483	0.579	0.845
N4	0	0.175	0.514	0.694	0.945
	5	0.171	0.510	0.687	0.939
	10	0.168	0.510	0.681	0.895
	15	0.165	0.493	0.661	0.884
	20	0.160	0.484	0.646	0.874
	25	0.159	0.483	0.594	0.863
N5	0	0.175	0.517	0.686	0.962
	5	0.171	0.513	0.671	0.929
	10	0.169	0.511	0.671	0.856
	15	0.165	0.491	0.610	0.856
	20	0.162	0.490	0.602	0.848
	25	0.154	0.489	0.581	0.839
N6	0	0.170	0.526	0.704	0.953
	5	0.170	0.524	0.691	0.874
	10	0.168	0.497	0.669	0.874
	15	0.166	0.494	0.663	0.856
	20	0.158	0.493	0.616	0.823
	25	0.154	0.491	0.601	0.817
N7	0	0.175	0.513	0.707	0.974
	5	0.170	0.504	0.707	0.941
	10	0.169	0.501	0.632	0.885
	15	0.162	0.500	0.627	0.884
	20	0.154	0.483	0.607	0.884
	25	0.154	0.480	0.596	0.852
N8	0	0.169	0.524	0.689	0.970
	5	0.165	0.523	0.664	0.958
	10	0.159	0.523	0.642	0.943
	15	0.159	0.518	0.638	0.918
	20	0.158	0.510	0.596	0.843
	25	0.154	0.490	0.587	0.824
N9	0	0.172	0.518	0.674	0.939
	5	0.166	0.511	0.661	0.929
	10	0.165	0.507	0.643	0.929
	15	0.158	0.504	0.623	0.872
	20	0.158	0.496	0.616	0.817
	25	0.154	0.487	0.585	0.817
N10	0	0.175	0.514	0.701	0.895
	5	0.171	0.506	0.696	0.889
	10	0.169	0.501	0.666	0.885
	15	0.169	0.500	0.666	0.845
	20	0.161	0.494	0.638	0.817
	25	0.154	0.484	0.626	0.808
N11	0	0.167	0.520	0.643	0.970
	5	0.162	0.520	0.661	0.968
	10	0.158	0.513	0.616	0.953
	15	0.155	0.511	0.674	0.904
	20	0.154	0.501	0.623	0.887
	25	0.152	0.490	0.585	0.857
N12	0	0.174	0.524	0.702	0.953
	5	0.164	0.510	0.694	0.899
	10	0.164	0.508	0.692	0.889
	15	0.157	0.497	0.686	0.884
	20	0.155	0.490	0.666	0.878
	25	0.154	0.487	0.578	0.846

Table 6. Table shows the F-NC scores obtained by aligning the first synthetic network with its noisy versions (Altered Networks). All synthetic networks are considered. The table shows significant improvements in the values of F-NC and hence in the quality of the alignments when considering networks with many colours with respect to the network with a single colour. The improvement is also stable for all the networks.

Network	Altered Networks	F-NC on 1 coloured version	F-NC on 2 coloured version	F-NC on 3 coloured version	F-NC on 4 coloured version
N1	0	0.416	0.706	0.841	0.98
	5	0.415	0.704	0.835	0.964
	10	0.413	0.699	0.798	0.957
	15	0.411	0.698	0.783	0.953
	20	0.404	0.695	0.762	0.943
	25	0.397	0.695	0.76	0.904
N2	0	0.414	0.715	0.841	0.99
	5	0.401	0.715	0.835	0.982
	10	0.399	0.714	0.788	0.976
	15	0.398	0.71	0.784	0.933
	20	0.397	0.707	0.763	0.919
	25	0.392	0.699	0.761	0.911
N3	0	0.408	0.706	0.843	0.96
	5	0.407	0.699	0.836	0.947
	10	0.404	0.704	0.788	0.939
	15	0.403	0.698	0.784	0.927
	20	0.395	0.695	0.763	0.927
	25	0.395	0.695	0.761	0.919
N4	0	0.418	0.717	0.833	0.972
	5	0.413	0.714	0.829	0.969
	10	0.41	0.714	0.825	0.946
	15	0.406	0.702	0.813	0.94
	20	0.4	0.696	0.804	0.935
	25	0.399	0.695	0.771	0.929
N5	0	0.418	0.719	0.828	0.981
	5	0.414	0.716	0.819	0.964
	10	0.411	0.715	0.819	0.925
	15	0.406	0.701	0.781	0.925
	20	0.402	0.7	0.776	0.921
	25	0.393	0.699	0.762	0.916
N6	0	0.412	0.725	0.839	0.976
	5	0.412	0.724	0.831	0.935
	10	0.41	0.705	0.818	0.935
	15	0.407	0.703	0.814	0.925
	20	0.397	0.702	0.785	0.907
	25	0.393	0.701	0.775	0.904
N7	0	0.418	0.716	0.841	0.987
	5	0.412	0.71	0.841	0.97
	10	0.411	0.708	0.795	0.941
	15	0.402	0.707	0.792	0.94
	20	0.393	0.695	0.779	0.94
	25	0.392	0.693	0.772	0.923
N8	0	0.411	0.724	0.83	0.985
	5	0.406	0.723	0.815	0.979
	10	0.399	0.723	0.801	0.971
	15	0.399	0.72	0.799	0.958
	20	0.398	0.714	0.772	0.918
	25	0.393	0.7	0.766	0.908
N9	0	0.415	0.72	0.821	0.969
	5	0.407	0.715	0.813	0.964
	10	0.406	0.712	0.802	0.964
	15	0.398	0.71	0.789	0.934
	20	0.398	0.704	0.785	0.904
	25	0.393	0.698	0.765	0.904
N10	0	0.418	0.717	0.837	0.946
	5	0.413	0.711	0.834	0.943
	10	0.411	0.708	0.816	0.941
	15	0.411	0.707	0.816	0.919
	20	0.401	0.703	0.799	0.904
	25	0.392	0.696	0.791	0.899
N11	0	0.409	0.721	0.802	0.985
	5	0.403	0.721	0.813	0.984
	10	0.397	0.716	0.785	0.976
	15	0.394	0.715	0.821	0.951
	20	0.393	0.708	0.789	0.942
	25	0.39	0.7	0.765	0.926
N12	0	0.417	0.724	0.838	0.976
	5	0.405	0.714	0.833	0.948
	10	0.405	0.713	0.832	0.943
	15	0.396	0.705	0.828	0.94
	20	0.394	0.7	0.816	0.937
	25	0.392	0.698	0.76	0.92

Table 7. NCV-GS^3 scores obtained by aligning the original Hetionet network with its noisy versions. Table shows a remarkable trend of improvement when considering networks with many colours. Results are similar for all the networks.

Network	Altered Networks	NCV-GS^3 on 1 coloured version	NCV-GS^3 on 2 coloured version	NCV-GS^3 on 3 coloured version	NCV-GS^3 on 4 coloured version
Hetionet	0% of noise	0.536	0.813	0.887	1
	5% of noise	0.532	0.803	0.882	0.996
	10% of noise	0.531	0.774	0.854	0.996
	15% of noise	0.53	0.763	0.843	0.966
	20% of noise	0.524	0.76	0.834	0.954
	25% of noise	0.512	0.745	0.825	0.951

Table 8. Table shows the GS^3 scores obtained by aligning the original Hetionet network with its noisy versions. Table shows that the alignment quality is constant when considering colours.

Network	Altered Networks	GS^3 on 1 coloured version	GS^3 on 2 coloured version	GS^3 on 3 coloured version	GS^3 on 4 coloured version
Hetionet	0% of noise	1	1	1	1
	5% of noise	1	1	1	1
	10% of noise	1	1	1	1
	15% of noise	1	1	1	1
	20% of noise	1	1	1	1
	25% of noise	1	1	1	1

Table 9. Table shows the NCV scores obtained by aligning the original Hetionet network with its noisy versions. Table shows a remarkable trend of improvement when considering networks with many colours for all the networks.

Network	Altered Networks	NCV on 1 coloured version	NCV on 2 coloured version	NCV on 3 coloured version	NCV on 4 coloured version
Hetionet	0% of noise	0.287	0.661	0.787	1
	5% of noise	0.283	0.645	0.778	0.992
	10% of noise	0.282	0.599	0.729	0.992
	15% of noise	0.281	0.582	0.711	0.933
	20% of noise	0.275	0.578	0.696	0.910
	25% of noise	0.262	0.555	0.681	0.904

Table 10. Table shows the P-NC scores obtained by aligning the original Hetionet network with its noisy versions. Table shows that the alignment quality is constant when considering colours.

Network	Altered Networks	P-NC on 1 coloured version	P-NC on 2 coloured version	P-NC on 3 coloured version	P-NC on 4 coloured version
Hetionet	0% of noise	1	1	1	1
	5% of noise	1	1	1	1
	10% of noise	1	1	1	1
	15% of noise	1	1	1	1
	20% of noise	1	1	1	1
	25% of noise	1	1	1	1

Table 11. Table shows the R-NC scores obtained by aligning the original Hetionet network with its noisy versions. Table shows a remarkable trend of improvement when considering networks with many colours.

Network	Altered Networks	R-NC on 1 coloured version	R-NC on 2 coloured version	R-NC on 3 coloured version	R-NC on 4 coloured version
Hetionet	0% of noise	0.175	0.523	0.785	0.978
	5% of noise	0.168	0.518	0.781	0.924
	10% of noise	0.167	0.517	0.692	0.906
	15% of noise	0.165	0.510	0.663	0.889
	20% of noise	0.157	0.496	0.654	0.852
	25% of noise	0.153	0.480	0.548	0.832

Table 12. Table shows the F-NC scores obtained by aligning the original Hetionet network with its noisy versions. Table shows a remarkable trend of improvement when considering networks with many colours for all the networks.

Network	Altered Networks	F-NC on 1 coloured version	F-NC on 2 coloured version	F-NC on 3 coloured version	F-NC on 4 coloured version
Hetionet	0% of noise	0.418	0.723	0.886	0.989
	5% of noise	0.41	0.72	0.884	0.961
	10% of noise	0.409	0.719	0.832	0.952
	15% of noise	0.406	0.714	0.814	0.943
	20% of noise	0.396	0.704	0.809	0.923
	25% of noise	0.391	0.693	0.74	0.912

Table 13. Table shows the NCV-GS^3 scores obtained by aligning the original synthetic network with its noisy versions (adding edges) for all the networks. Table shows a significant improvement of the quality of the alignments when considering networks with many colours. The improvement is also stable for all the networks.

Network	Altered Networks	NCV-GS^3 on 1 coloured version	NCV-GS^3 on 2 coloured version	NCV-GS^3 on 3 coloured version	NCV-GS^3 on 4 coloured version
N1	0	0.426	0.521	0.677	0.893
	5	0.425	0.518	0.674	0.89
	10	0.423	0.516	0.656	0.889
	15	0.419	0.516	0.646	0.87
	20	0.419	0.508	0.644	0.869
	25	0.408	0.507	0.632	0.868
N2	0	0.445	0.527	0.673	0.901
	5	0.433	0.527	0.672	0.9
	10	0.428	0.526	0.663	0.875
	15	0.426	0.507	0.659	0.873
	20	0.422	0.504	0.635	0.872
	25	0.414	0.503	0.615	0.87
N3	0	0.445	0.53	0.678	0.889
	5	0.442	0.527	0.675	0.882
	10	0.438	0.522	0.669	0.88
	15	0.436	0.512	0.663	0.877
	20	0.429	0.509	0.615	0.862
	25	0.416	0.504	0.611	0.861
N4	0	0.447	0.525	0.679	0.9
	5	0.427	0.523	0.648	0.898
	10	0.421	0.52	0.648	0.898
	15	0.418	0.509	0.64	0.894
	20	0.414	0.505	0.625	0.886
	25	0.407	0.503	0.616	0.869
N5	0	0.444	0.517	0.675	0.895
	5	0.431	0.516	0.673	0.888
	10	0.426	0.514	0.672	0.884
	15	0.417	0.504	0.642	0.881
	20	0.415	0.503	0.624	0.875
	25	0.411	0.503	0.611	0.866
N6	0	0.435	0.529	0.673	0.891
	5	0.434	0.524	0.672	0.883
	10	0.434	0.517	0.661	0.879
	15	0.424	0.507	0.65	0.878
	20	0.422	0.505	0.634	0.873
	25	0.411	0.505	0.633	0.864
N7	0	0.435	0.527	0.665	0.896
	5	0.433	0.526	0.661	0.896
	10	0.429	0.525	0.654	0.89
	15	0.427	0.522	0.65	0.889
	20	0.419	0.52	0.625	0.88
	25	0.409	0.508	0.616	0.869
N8	0	0.449	0.527	0.678	0.899
	5	0.441	0.522	0.672	0.887
	10	0.44	0.522	0.663	0.885
	15	0.436	0.51	0.646	0.876
	20	0.429	0.506	0.643	0.87
	25	0.417	0.501	0.637	0.867
N9	0	0.448	0.529	0.661	0.901
	5	0.446	0.52	0.65	0.887
	10	0.421	0.513	0.644	0.885
	15	0.419	0.512	0.637	0.885
	20	0.411	0.506	0.63	0.881
	25	0.407	0.505	0.613	0.872
N10	0	0.446	0.53	0.658	0.894
	5	0.446	0.53	0.657	0.893
	10	0.439	0.526	0.656	0.891
	15	0.431	0.516	0.648	0.888
	20	0.414	0.508	0.641	0.881
	25	0.411	0.506	0.624	0.867
N11	0	0.439	0.521	0.639	0.899
	5	0.435	0.517	0.634	0.887
	10	0.422	0.515	0.624	0.878
	15	0.406	0.515	0.621	0.874
	20	0.406	0.504	0.615	0.869
	25	0.405	0.502	0.614	0.866
N12	0	0.449	0.521	0.663	0.898
	5	0.439	0.521	0.657	0.898
	10	0.437	0.512	0.656	0.886
	15	0.417	0.508	0.629	0.879
	20	0.417	0.507	0.616	0.873
	25	0.407	0.503	0.614	0.862

Table 14. Table shows GS^3 scores obtained by aligning the original synthetic network with its noisy versions (adding edges) for all the networks. Table shows that the alignment quality does not get worse when considering colors.

Network	Altered Networks	GS^3 on 1 coloured version	GS^3 on 2 coloured version	GS^3 on 3 coloured version	GS^3 on 4 coloured version
N1	0	1	1	1	1
	5	1	1	1	1
	10	1	1	1	1
	15	1	1	1	1
	20	1	1	1	1
	25	1	1	1	1
N2	0	1	1	1	1
	5	1	1	1	1
	10	1	1	1	1
	15	1	1	1	1
	20	1	1	1	1
	25	1	1	1	1
N3	0	1	1	1	1
	5	1	1	1	1
	10	1	1	1	1
	15	1	1	1	1
	20	1	1	1	1
	25	1	1	1	1
N4	0	1	1	1	1
	5	1	1	1	1
	10	1	1	1	1
	15	1	1	1	1
	20	1	1	1	1
	25	1	1	1	1
N5	0	1	1	1	1
	5	1	1	1	1
	10	1	1	1	1
	15	1	1	1	1
	20	1	1	1	1
	25	1	1	1	1
N6	0	1	1	1	1
	5	1	1	1	1
	10	1	1	1	1
	15	1	1	1	1
	20	1	1	1	1
	25	1	1	1	1
N7	0	1	1	1	1
	5	1	1	1	1
	10	1	1	1	1
	15	1	1	1	1
	20	1	1	1	1
	25	1	1	1	1
N8	0	1	1	1	1
	5	1	1	1	1
	10	1	1	1	1
	15	1	1	1	1
	20	1	1	1	1
	25	1	1	1	1
N9	0	1	1	1	1
	5	1	1	1	1
	10	1	1	1	1
	15	1	1	1	1
	20	1	1	1	1
	25	1	1	1	1
N10	0	1	1	1	1
	5	1	1	1	1
	10	1	1	1	1
	15	1	1	1	1
	20	1	1	1	1
	25	1	1	1	1
N11	0	1	1	1	1
	5	1	1	1	1
	10	1	1	1	1
	15	1	1	1	1
	20	1	1	1	1
	25	1	1	1	1
N12	0	1	1	1	1
	5	1	1	1	1
	10	1	1	1	1
	15	1	1	1	1
	20	1	1	1	1
	25	1	1	1	1

Quality of the Alignments for Hetionet network with noise.

Quality of the alignments for synthetic heterogeneous networks obtained by aligning each network with its noisy versions built by adding a percentage of nodes

Table 25, Table 26, Table 27, Table 28, Table 29, Table 30 report the NCV-GS^3, GS^3, NCV, P-NC, R-NC, F-NC measures computed on each module for the four versions of synthetic heterogeneous networks obtained by aligning each network with its noisy versions built by adding a percentage of nodes.

Table 31, Table 32, Table 33, Table 34, Table 35, Table 36 report the NCV-GS^3, GS^3, NCV, P-NC, R-NC, F-NC measures computed on each module for the four versions of the Hetionet network obtained by aligning each network with its noisy counterpart built by adding a percentage of nodes.

Quality of the alignments of Hetionet network obtained by aligning each network with its noisy versions built by adding a percentage of nodes

Table 15. Table shows the NCV scores obtained by aligning the original synthetic network with its noisy versions for all the networks. The altered networks are generated by randomly adding edges. Table shows a significant improvement of the quality of the alignments when considering networks with many colours. The improvement is also stable for all the networks.

Network	Altered Networks	NCV on 1 coloured version	NCV on 2 coloured version	NCV on 3 coloured version	NCV on 4 coloured version
N1	0	0.181	0.271	0.458	0.797
	5	0.181	0.268	0.454	0.792
	10	0.179	0.266	0.43	0.79
	15	0.176	0.266	0.417	0.757
	20	0.176	0.258	0.415	0.755
	25	0.166	0.257	0.399	0.753
N2	0	0.198	0.278	0.453	0.812
	5	0.187	0.278	0.452	0.81
	10	0.183	0.277	0.44	0.766
	15	0.181	0.257	0.434	0.762
	20	0.178	0.254	0.403	0.76
	25	0.171	0.253	0.378	0.757
N3	0	0.198	0.281	0.46	0.79
	5	0.195	0.278	0.456	0.778
	10	0.192	0.272	0.448	0.774
	15	0.19	0.262	0.44	0.769
	20	0.184	0.259	0.378	0.743
	25	0.173	0.254	0.373	0.741
N4	0	0.2	0.276	0.461	0.81
	5	0.182	0.274	0.42	0.806
	10	0.177	0.27	0.42	0.806
	15	0.175	0.259	0.41	0.799
	20	0.171	0.255	0.391	0.785
	25	0.166	0.253	0.379	0.755
N5	0	0.197	0.267	0.456	0.801
	5	0.186	0.266	0.453	0.789
	10	0.181	0.264	0.452	0.781
	15	0.174	0.254	0.412	0.776
	20	0.172	0.253	0.389	0.766
	25	0.169	0.253	0.373	0.75
N6	0	0.189	0.28	0.453	0.794
	5	0.188	0.275	0.452	0.78
	10	0.188	0.267	0.437	0.773
	15	0.18	0.257	0.423	0.771
	20	0.178	0.255	0.402	0.762
	25	0.169	0.255	0.401	0.746
N7	0	0.189	0.278	0.442	0.803
	5	0.187	0.277	0.437	0.803
	10	0.184	0.276	0.428	0.792
	15	0.182	0.272	0.423	0.79
	20	0.176	0.27	0.391	0.774
	25	0.167	0.258	0.379	0.755
N8	0	0.202	0.278	0.46	0.808
	5	0.194	0.272	0.452	0.787
	10	0.194	0.272	0.44	0.783
	15	0.19	0.26	0.417	0.767
	20	0.184	0.256	0.413	0.757
	25	0.174	0.251	0.406	0.752
N9	0	0.201	0.28	0.437	0.812
	5	0.199	0.27	0.423	0.787
	10	0.177	0.263	0.415	0.783
	15	0.176	0.262	0.406	0.783
	20	0.169	0.256	0.397	0.776
	25	0.166	0.255	0.376	0.76
N10	0	0.199	0.281	0.433	0.799
	5	0.199	0.281	0.432	0.797
	10	0.193	0.277	0.43	0.794
	15	0.186	0.266	0.42	0.789
	20	0.171	0.258	0.411	0.776
	25	0.169	0.256	0.389	0.752
N11	0	0.193	0.271	0.408	0.808
	5	0.189	0.267	0.402	0.787
	10	0.178	0.265	0.389	0.771
	15	0.165	0.265	0.386	0.764
	20	0.165	0.254	0.378	0.755
	25	0.164	0.252	0.377	0.75
N12	0	0.202	0.271	0.44	0.806
	5	0.193	0.271	0.432	0.806
	10	0.191	0.262	0.43	0.785
	15	0.174	0.258	0.396	0.773
	20	0.174	0.257	0.379	0.762
	25	0.166	0.253	0.377	0.743

Table 16. P-NC scores obtained by aligning the original synthetic network with its noisy versions (adding edges) for all the networks. Table shows that the alignment quality is constant by considering networks with many colours.

Network	Altered Networks	P-NC on 1 coloured version	P-NC on 2 coloured version	P-NC on 3 coloured version	P-NC on 4 coloured version
N1	0	1	1	1	1
	5	1	1	1	1
	10	1	1	1	1
	15	1	1	1	1
	20	1	1	1	1
	25	1	1	1	1
N2	0	1	1	1	1
	5	1	1	1	1
	10	1	1	1	1
	15	1	1	1	1
	20	1	1	1	1
	25	1	1	1	1
N3	0	1	1	1	1
	5	1	1	1	1
	10	1	1	1	1
	15	1	1	1	1
	20	1	1	1	1
	25	1	1	1	1
N4	0	1	1	1	1
	5	1	1	1	1
	10	1	1	1	1
	15	1	1	1	1
	20	1	1	1	1
	25	1	1	1	1
N5	0	1	1	1	1
	5	1	1	1	1
	10	1	1	1	1
	15	1	1	1	1
	20	1	1	1	1
	25	1	1	1	1
N6	0	1	1	1	1
	5	1	1	1	1
	10	1	1	1	1
	15	1	1	1	1
	20	1	1	1	1
	25	1	1	1	1
N7	0	1	1	1	1
	5	1	1	1	1
	10	1	1	1	1
	15	1	1	1	1
	20	1	1	1	1
	25	1	1	1	1
N8	0	1	1	1	1
	5	1	1	1	1
	10	1	1	1	1
	15	1	1	1	1
	20	1	1	1	1
	25	1	1	1	1
N9	0	1	1	1	1
	5	1	1	1	1
	10	1	1	1	1
	15	1	1	1	1
	20	1	1	1	1
	25	1	1	1	1
N10	0	1	1	1	1
	5	1	1	1	1
	10	1	1	1	1
	15	1	1	1	1
	20	1	1	1	1
	25	1	1	1	1
N11	0	1	1	1	1
	5	1	1	1	1
	10	1	1	1	1
	15	1	1	1	1
	20	1	1	1	1
	25	1	1	1	1
N12	0	1	1	1	1
	5	1	1	1	1
	10	1	1	1	1
	15	1	1	1	1
	20	1	1	1	1
	25	1	1	1	1

Table 17. Table shows the R-NCscores obtained by aligning the original synthetic network with its noisy versions for all the networks. The altered networks are generated by randomly adding edges. Table shows a significant improvement of the quality of the alignments when considering networks with many colours. The improvement is also stable for all the networks.

Network	Altered Networks	R-NC on 1 coloured version	R-NC on 2 coloured version	R-NC on 3 coloured version	R-NC on 4 coloured version
N1	0	0.138	0.476	0.623	0.651
	5	0.131	0.453	0.612	0.651
	10	0.116	0.446	0.607	0.651
	15	0.109	0.441	0.548	0.65
	20	0.1	0.44	0.546	0.646
	25	0.096	0.386	0.513	0.646
N2	0	0.137	0.464	0.634	0.654
	5	0.135	0.464	0.616	0.653
	10	0.122	0.462	0.616	0.646
	15	0.121	0.426	0.596	0.645
	20	0.114	0.394	0.567	0.645
	25	0.113	0.384	0.566	0.64
N3	0	0.158	0.424	0.607	0.654
	5	0.152	0.423	0.596	0.653
	10	0.147	0.367	0.594	0.651
	15	0.144	0.366	0.498	0.646
	20	0.127	0.365	0.497	0.645
	25	0.096	0.361	0.497	0.642
N4	0	0.141	0.423	0.627	0.656
	5	0.141	0.411	0.624	0.656
	10	0.114	0.41	0.618	0.654
	15	0.11	0.401	0.516	0.653
	20	0.105	0.376	0.514	0.65
	25	0.095	0.361	0.513	0.646
N5	0	0.149	0.454	0.634	0.651
	5	0.149	0.434	0.608	0.651
	10	0.141	0.434	0.605	0.65
	15	0.129	0.432	0.601	0.648
	20	0.115	0.425	0.529	0.648
	25	0.108	0.423	0.507	0.643
N6	0	0.152	0.466	0.619	0.656
	5	0.126	0.425	0.618	0.653
	10	0.116	0.412	0.599	0.653
	15	0.111	0.402	0.591	0.65
	20	0.1	0.397	0.51	0.646
	25	0.097	0.366	0.501	0.643
N7	0	0.143	0.429	0.63	0.656
	5	0.141	0.428	0.564	0.648
	10	0.13	0.41	0.562	0.646
	15	0.123	0.404	0.557	0.643
	20	0.106	0.399	0.534	0.642
	25	0.101	0.387	0.497	0.64
N8	0	0.135	0.471	0.638	0.656
	5	0.127	0.45	0.612	0.653
	10	0.12	0.429	0.548	0.653
	15	0.118	0.419	0.539	0.653
	20	0.109	0.381	0.531	0.65
	25	0.095	0.376	0.503	0.646
N9	0	0.138	0.434	0.635	0.654
	5	0.137	0.428	0.632	0.651
	10	0.135	0.411	0.566	0.65
	15	0.128	0.403	0.543	0.646
	20	0.124	0.399	0.508	0.645
	25	0.102	0.376	0.503	0.643
N10	0	0.151	0.456	0.579	0.653
	5	0.145	0.436	0.557	0.648
	10	0.135	0.433	0.557	0.648
	15	0.127	0.433	0.551	0.645
	20	0.119	0.419	0.54	0.645
	25	0.099	0.402	0.529	0.64
N11	0	0.15	0.471	0.63	0.656
	5	0.132	0.462	0.607	0.656
	10	0.116	0.458	0.576	0.653
	15	0.106	0.44	0.572	0.648
	20	0.094	0.416	0.567	0.648
	25	0.093	0.394	0.533	0.642
N12	0	0.144	0.468	0.619	0.654
	5	0.132	0.465	0.619	0.651
	10	0.122	0.444	0.582	0.65
	15	0.122	0.382	0.548	0.648
	20	0.107	0.375	0.503	0.645
	25	0.1	0.366	0.498	0.643

Table 18. Table shows the F-NC scores obtained by aligning the original synthetic network with its noisy versions for all the networks. The altered networks are generated by randomly adding edges. Table shows a significant improvement of the quality of the alignments when considering networks with many colours. The improvement is also stable for all the networks.

Network	Altered Networks	F-NC on 1 coloured version	F-NC on 2 coloured version	F-NC on 3 coloured version	F-NC on 4 coloured version
N1	0	0.371	0.69	0.789	0.807
	5	0.362	0.673	0.782	0.807
	10	0.341	0.668	0.779	0.807
	15	0.33	0.664	0.74	0.806
	20	0.317	0.663	0.739	0.804
	25	0.31	0.621	0.716	0.804
N2	0	0.37	0.681	0.796	0.809
	5	0.367	0.681	0.785	0.808
	10	0.35	0.68	0.785	0.804
	15	0.348	0.653	0.772	0.803
	20	0.338	0.628	0.753	0.803
	25	0.336	0.62	0.752	0.8
N3	0	0.398	0.651	0.779	0.809
	5	0.39	0.65	0.772	0.808
	10	0.383	0.606	0.771	0.807
	15	0.38	0.605	0.706	0.804
	20	0.357	0.604	0.705	0.803
	25	0.31	0.601	0.705	0.801
N4	0	0.376	0.65	0.792	0.81
	5	0.375	0.641	0.79	0.81
	10	0.337	0.64	0.786	0.809
	15	0.332	0.633	0.718	0.808
	20	0.324	0.613	0.717	0.806
	25	0.309	0.601	0.716	0.804
N5	0	0.386	0.674	0.796	0.807
	5	0.386	0.659	0.78	0.807
	10	0.376	0.659	0.778	0.806
	15	0.359	0.657	0.775	0.805
	20	0.339	0.652	0.727	0.805
	25	0.328	0.65	0.712	0.802
N6	0	0.39	0.683	0.787	0.81
	5	0.355	0.652	0.786	0.808
	10	0.34	0.642	0.774	0.808
	15	0.333	0.634	0.769	0.806
	20	0.317	0.63	0.714	0.804
	25	0.311	0.605	0.708	0.802
N7	0	0.378	0.655	0.794	0.81
	5	0.376	0.654	0.751	0.805
	10	0.36	0.64	0.75	0.804
	15	0.351	0.636	0.746	0.802
	20	0.325	0.632	0.731	0.801
	25	0.318	0.622	0.705	0.8
N8	0	0.368	0.686	0.799	0.81
	5	0.356	0.671	0.782	0.808
	10	0.346	0.655	0.74	0.808
	15	0.343	0.647	0.734	0.808
	20	0.33	0.617	0.729	0.806
	25	0.309	0.613	0.709	0.804
N9	0	0.371	0.659	0.797	0.809
	5	0.37	0.654	0.795	0.807
	10	0.368	0.641	0.752	0.806
	15	0.358	0.635	0.737	0.804
	20	0.352	0.632	0.713	0.803
	25	0.32	0.613	0.709	0.802
N10	0	0.388	0.675	0.761	0.808
	5	0.381	0.66	0.746	0.805
	10	0.367	0.658	0.746	0.805
	15	0.357	0.658	0.742	0.803
	20	0.345	0.647	0.735	0.803
	25	0.314	0.634	0.727	0.8
N11	0	0.387	0.686	0.794	0.81
	5	0.363	0.68	0.779	0.81
	10	0.341	0.677	0.759	0.808
	15	0.326	0.663	0.756	0.805
	20	0.307	0.645	0.753	0.805
	25	0.305	0.628	0.73	0.801
N12	0	0.379	0.684	0.787	0.809
	5	0.363	0.682	0.787	0.807
	10	0.35	0.666	0.763	0.806
	15	0.349	0.618	0.74	0.805
	20	0.327	0.612	0.709	0.803
	25	0.317	0.605	0.706	0.802

Table 19. Table summarises NCV-GS^3 scores obtained by aligning the original Hetionet network with its noisy versions obtained by adding edges to the first one. Table shows a remarkable trend of improvement when considering networks with many colours.

Network	Altered Networks	NCV-GS^3 on 1 coloured version	NCV-GS^3 on 2 coloured version	NCV-GS^3 on 3 coloured version	NCV-GS^3 on 4 coloured version
Hetionet	0% of noise	0.448	0.521	0.673	0.898
	5% of noise	0.446	0.517	0.672	0.896
	10% of noise	0.421	0.515	0.663	0.89
	15% of noise	0.419	0.515	0.659	0.889
	20% of noise	0.411	0.504	0.635	0.88
	25% of noise	0.407	0.502	0.615	0.869

Table 20. Table summarises GS^3 scores obtained by aligning the original Hetionet network with its noisy versions. Altered networks are obtained by adding edges randomly. Table shows that the alignment quality is constant when considering networks with many colours.

Network	Altered Networks	GS^3 on 1 coloured version	GS^3 on 2 coloured version	GS^3 on 3 coloured version	GS^3 on 4 coloured version
Hetionet	0% of noise	1	1	1	1
	5% of noise	1	1	1	1
	10% of noise	1	1	1	1
	15% of noise	1	1	1	1
	20% of noise	1	1	1	1
	25% of noise	1	1	1	1

Table 21. Table summarises the NCV scores obtained by aligning the original Hetionet network with its noisy versions. Altered networks presents more random edges. Table shows a remarkable trend of improvement when considering networks with many colours for all the networks.

Network	Altered Networks	NCV on 1 coloured version	NCV on 2 coloured version	NCV on 3 coloured version	NCV on 4 coloured version
Hetionet	0% of noise	0.201	0.271	0.453	0.803
	5% of noise	0.199	0.267	0.452	0.803
	10% of noise	0.177	0.265	0.44	0.792
	15% of noise	0.176	0.265	0.434	0.79
	20% of noise	0.169	0.254	0.403	0.774
	25% of noise	0.166	0.252	0.378	0.755

Table 22. Table summarises P-NC scores obtained by aligning the original Hetionet network with its noisy versions. Table shows that the alignment quality is constant when considering colours.

Network	Altered Networks	P-NC on 1 coloured version	P-NC on 2 coloured version	P-NC on 3 coloured version	P-NC on 4 coloured version
Hetionet	0% of noise	1	1	1	1
	5% of noise	1	1	1	1
	10% of noise	1	1	1	1
	15% of noise	1	1	1	1
	20% of noise	1	1	1	1
	25% of noise	1	1	1	1

Table 23. Table summarises the R-NC scores obtained by aligning the original Hetionet network with its noisy versions when adding edges. Table shows a remarkable trend of improvement when considering networks with many colours.

Network	Altered Networks	R-NC on 1 coloured version	R-NC on 2 coloured version	R-NC on 3 coloured version	R-NC on 4 coloured version
Hetionet	0% of noise	0.158	0.471	0.638	0.656
	5% of noise	0.152	0.462	0.612	0.648
	10% of noise	0.147	0.458	0.548	0.646
	15% of noise	0.144	0.44	0.539	0.643
	20% of noise	0.127	0.416	0.531	0.642
	25% of noise	0.096	0.394	0.503	0.64

Table 24. Table summarises the F-NC scores obtained by aligning the original Hetionet network with its noisy versions when adding edges. Table shows a remarkable trend of improvement when considering networks with many colours.

Network	Altered Networks	F-NC on 1 coloured version	F-NC on 2 coloured version	F-NC on 3 coloured version	F-NC on 4 coloured version
Hetionet	0% of noise	0.398	0.686	0.799	0.81
	5% of noise	0.39	0.68	0.782	0.805
	10% of noise	0.383	0.677	0.74	0.804
	15% of noise	0.38	0.663	0.734	0.802
	20% of noise	0.357	0.645	0.729	0.801
	25% of noise	0.31	0.628	0.709	0.8

Table 25. The table shows the NCV-GS^3 scores obtained by aligning the original synthetic network with its noisy versions when adding nodes for all the networks. Table shows a significant improvement of the quality of the alignments when considering networks with many colours. The improvement is also stable for all the networks.

Network	Altered Networks	NCV-GS^3 on 1 coloured version	NCV-GS^3 on 2 coloured version	NCV-GS^3 on 3 coloured version	NCV-GS^3 on 4 coloured version
N1	0	0.281	0.409	0.564	0.746
	5	0.28	0.407	0.559	0.732
	10	0.278	0.405	0.557	0.723
	15	0.274	0.402	0.552	0.705
	20	0.266	0.397	0.549	0.686
	25	0.257	0.395	0.52	0.683
N2	0	0.299	0.403	0.566	0.719
	5	0.295	0.402	0.564	0.717
	10	0.292	0.4	0.55	0.708
	15	0.292	0.396	0.548	0.69
	20	0.257	0.389	0.53	0.681
	25	0.251	0.384	0.505	0.68
N3	0	0.295	0.401	0.559	0.746
	5	0.288	0.398	0.557	0.72
	10	0.277	0.392	0.555	0.712
	15	0.27	0.391	0.555	0.708
	20	0.264	0.387	0.538	0.696
	25	0.25	0.382	0.521	0.687
N4	0	0.281	0.398	0.558	0.746
	5	0.278	0.39	0.554	0.741
	10	0.277	0.388	0.55	0.72
	15	0.275	0.386	0.538	0.707
	20	0.266	0.382	0.535	0.698
	25	0.263	0.38	0.508	0.683
N5	0	0.296	0.403	0.566	0.747
	5	0.27	0.402	0.556	0.742
	10	0.267	0.399	0.554	0.736
	15	0.265	0.399	0.534	0.726
	20	0.264	0.398	0.532	0.689
	25	0.258	0.381	0.51	0.688
N6	0	0.297	0.407	0.558	0.744
	5	0.297	0.4	0.547	0.733
	10	0.288	0.388	0.54	0.729
	15	0.286	0.382	0.531	0.718
	20	0.269	0.381	0.523	0.683
	25	0.255	0.38	0.518	0.681
N7	0	0.292	0.404	0.556	0.737
	5	0.274	0.4	0.545	0.705
	10	0.272	0.393	0.517	0.704
	15	0.271	0.391	0.51	0.691
	20	0.27	0.389	0.506	0.689
	25	0.256	0.386	0.503	0.688
N8	0	0.294	0.41	0.564	0.741
	5	0.292	0.402	0.546	0.731
	10	0.291	0.401	0.524	0.716
	15	0.287	0.395	0.52	0.712
	20	0.281	0.388	0.507	0.705
	25	0.273	0.388	0.504	0.68
N9	0	0.294	0.406	0.565	0.728
	5	0.289	0.399	0.56	0.721
	10	0.261	0.386	0.537	0.719
	15	0.257	0.384	0.513	0.704
	20	0.257	0.383	0.506	0.687
	25	0.257	0.382	0.503	0.687
N10	0	0.295	0.409	0.56	0.73
	5	0.284	0.404	0.556	0.714
	10	0.277	0.395	0.546	0.702
	15	0.257	0.393	0.544	0.7
	20	0.256	0.392	0.531	0.697
	25	0.25	0.381	0.501	0.689
N11	0	0.286	0.404	0.555	0.73
	5	0.28	0.404	0.55	0.718
	10	0.274	0.401	0.535	0.711
	15	0.271	0.396	0.512	0.708
	20	0.269	0.392	0.509	0.694
	25	0.256	0.381	0.507	0.687
N12	0	0.297	0.399	0.558	0.741
	5	0.287	0.399	0.558	0.727
	10	0.266	0.398	0.531	0.722
	15	0.262	0.397	0.521	0.7
	20	0.261	0.39	0.52	0.693
	25	0.256	0.387	0.517	0.682

Table 26. Table shows the GS^3 scores obtained by aligning the original synthetic network with its noisy versions when adding nodes for all the networks. Table shows that the alignment quality is constant.

Network	Altered Networks	GS^3 on 1 coloured version	GS^3 on 2 coloured version	GS^3 on 3 coloured version	GS^3 on 4 coloured version
N1	0	1	1	1	1
	5	1	1	1	1
	10	1	1	1	1
	15	1	1	1	1
	20	1	1	1	1
	25	1	1	1	1
N2	0	1	1	1	1
	5	1	1	1	1
	10	1	1	1	1
	15	1	1	1	1
	20	1	1	1	1
	25	1	1	1	1
N3	0	1	1	1	1
	5	1	1	1	1
	10	1	1	1	1
	15	1	1	1	1
	20	1	1	1	1
	25	1	1	1	1
N4	0	1	1	1	1
	5	1	1	1	1
	10	1	1	1	1
	15	1	1	1	1
	20	1	1	1	1
	25	1	1	1	1
N5	0	1	1	1	1
	5	1	1	1	1
	10	1	1	1	1
	15	1	1	1	1
	20	1	1	1	1
	25	1	1	1	1
N6	0	1	1	1	1
	5	1	1	1	1
	10	1	1	1	1
	15	1	1	1	1
	20	1	1	1	1
	25	1	1	1	1
N7	0	1	1	1	1
	5	1	1	1	1
	10	1	1	1	1
	15	1	1	1	1
	20	1	1	1	1
	25	1	1	1	1
N8	0	1	1	1	1
	5	1	1	1	1
	10	1	1	1	1
	15	1	1	1	1
	20	1	1	1	1
	25	1	1	1	1
N9	0	1	1	1	1
	5	1	1	1	1
	10	1	1	1	1
	15	1	1	1	1
	20	1	1	1	1
	25	1	1	1	1
N10	0	1	1	1	1
	5	1	1	1	1
	10	1	1	1	1
	15	1	1	1	1
	20	1	1	1	1
	25	1	1	1	1
N11	0	1	1	1	1
	5	1	1	1	1
	10	1	1	1	1
	15	1	1	1	1
	20	1	1	1	1
	25	1	1	1	1
N12	0	1	1	1	1
	5	1	1	1	1
	10	1	1	1	1
	15	1	1	1	1
	20	1	1	1	1
	25	1	1	1	1

Table 27. The table shows the NCV scores obtained by aligning the original synthetic network with its noisy versions when adding nodes for all the networks. Table shows a significant improvement of the quality of the alignments when considering networks with many colours. The improvement is also stable for all the networks.

Network	Altered Networks	NCV on 1 coloured version	NCV on 2 coloured version	NCV on 3 coloured version	NCV on 4 coloured version
N1	0	0.079	0.167	0.36	0.557
	5	0.078	0.166	0.356	0.536
	10	0.077	0.164	0.356	0.523
	15	0.075	0.162	0.352	0.497
	20	0.071	0.158	0.341	0.471
	25	0.066	0.156	0.335	0.466
N2	0	0.089	0.162	0.359	0.517
	5	0.087	0.162	0.355	0.514
	10	0.085	0.16	0.35	0.501
	15	0.085	0.157	0.338	0.476
	20	0.066	0.151	0.338	0.464
	25	0.063	0.147	0.326	0.462
N3	0	0.087	0.161	0.355	0.557
	5	0.083	0.158	0.342	0.518
	10	0.077	0.154	0.341	0.507
	15	0.073	0.153	0.338	0.501
	20	0.07	0.15	0.336	0.484
	25	0.062	0.146	0.332	0.472
N4	0	0.079	0.158	0.36	0.557
	5	0.077	0.152	0.36	0.549
	10	0.077	0.151	0.353	0.518
	15	0.076	0.149	0.339	0.5
	20	0.071	0.146	0.338	0.487
	25	0.069	0.144	0.336	0.466
N5	0	0.088	0.162	0.358	0.558
	5	0.073	0.162	0.352	0.551
	10	0.071	0.159	0.342	0.542
	15	0.07	0.159	0.342	0.527
	20	0.07	0.158	0.333	0.475
	25	0.067	0.145	0.333	0.473
N6	0	0.088	0.166	0.361	0.554
	5	0.088	0.16	0.36	0.537
	10	0.083	0.151	0.348	0.531
	15	0.082	0.146	0.347	0.516
	20	0.072	0.145	0.346	0.466
	25	0.065	0.144	0.327	0.464
N7	0	0.085	0.163	0.358	0.543
	5	0.075	0.16	0.345	0.497
	10	0.074	0.154	0.338	0.496
	15	0.073	0.153	0.338	0.477
	20	0.073	0.151	0.336	0.475
	25	0.066	0.149	0.333	0.473
N8	0	0.086	0.168	0.36	0.549
	5	0.085	0.162	0.349	0.534
	10	0.085	0.161	0.342	0.513
	15	0.082	0.156	0.34	0.507
	20	0.079	0.151	0.334	0.497
	25	0.075	0.151	0.326	0.462
N9	0	0.086	0.165	0.361	0.53
	5	0.084	0.159	0.361	0.52
	10	0.068	0.149	0.35	0.517
	15	0.066	0.147	0.35	0.496
	20	0.066	0.147	0.342	0.472
	25	0.066	0.146	0.326	0.472
N10	0	0.087	0.167	0.358	0.533
	5	0.081	0.163	0.354	0.51
	10	0.077	0.156	0.353	0.493
	15	0.066	0.154	0.353	0.49
	20	0.066	0.154	0.341	0.486
	25	0.062	0.145	0.336	0.475
N11	0	0.082	0.163	0.358	0.533
	5	0.078	0.163	0.345	0.516
	10	0.075	0.161	0.345	0.506
	15	0.073	0.157	0.335	0.501
	20	0.072	0.154	0.333	0.482
	25	0.066	0.145	0.325	0.472
N12	0	0.088	0.159	0.352	0.549
	5	0.082	0.159	0.349	0.529
	10	0.071	0.158	0.348	0.521
	15	0.069	0.158	0.338	0.49
	20	0.068	0.152	0.336	0.48
	25	0.066	0.15	0.326	0.465

Table 28. P-NC scores obtained by aligning the original synthetic network with its noisy versions for all the networks. Networks are obtained by adding nodes. Table shows that the alignment quality is constant.

Network	Altered Networks	P-NC on 1 coloured version	P-NC on 2 coloured version	P-NC on 3 coloured version	P-NC on 4 coloured version
N1	0	1	1	1	1
	5	1	1	1	1
	10	1	1	1	1
	15	1	1	1	1
	20	1	1	1	1
	25	1	1	1	1
N2	0	1	1	1	1
	5	1	1	1	1
	10	1	1	1	1
	15	1	1	1	1
	20	1	1	1	1
	25	1	1	1	1
N3	0	1	1	1	1
	5	1	1	1	1
	10	1	1	1	1
	15	1	1	1	1
	20	1	1	1	1
	25	1	1	1	1
N4	0	1	1	1	1
	5	1	1	1	1
	10	1	1	1	1
	15	1	1	1	1
	20	1	1	1	1
	25	1	1	1	1
N5	0	1	1	1	1
	5	1	1	1	1
	10	1	1	1	1
	15	1	1	1	1
	20	1	1	1	1
	25	1	1	1	1
N6	0	1	1	1	1
	5	1	1	1	1
	10	1	1	1	1
	15	1	1	1	1
	20	1	1	1	1
	25	1	1	1	1
N7	0	1	1	1	1
	5	1	1	1	1
	10	1	1	1	1
	15	1	1	1	1
	20	1	1	1	1
	25	1	1	1	1
N8	0	1	1	1	1
	5	1	1	1	1
	10	1	1	1	1
	15	1	1	1	1
	20	1	1	1	1
	25	1	1	1	1
N9	0	1	1	1	1
	5	1	1	1	1
	10	1	1	1	1
	15	1	1	1	1
	20	1	1	1	1
	25	1	1	1	1
N10	0	1	1	1	1
	5	1	1	1	1
	10	1	1	1	1
	15	1	1	1	1
	20	1	1	1	1
	25	1	1	1	1
N11	0	1	1	1	1
	5	1	1	1	1
	10	1	1	1	1
	15	1	1	1	1
	20	1	1	1	1
	25	1	1	1	1
N12	0	1	1	1	1
	5	1	1	1	1
	10	1	1	1	1
	15	1	1	1	1
	20	1	1	1	1
	25	1	1	1	1

Table 29. The table shows the R-NC scores obtained by aligning the original synthetic network with its noisy versions when adding nodes for all the networks. Table shows a significant improvement of the quality of the alignments when considering networks with many colours. The improvement is also stable for all the networks.

Network	Altered Networks	R-NC on 1 coloured version	R-NC on 2 coloured version	R-NC on 3 coloured version	R-NC on 4 coloured version
N1	0	0.084	0.167	0.101	0.539
	5	0.084	0.162	0.097	0.523
	10	0.075	0.154	0.096	0.508
	15	0.066	0.151	0.093	0.497
	20	0.065	0.151	0.091	0.491
	25	0.064	0.149	0.073	0.491
N2	0	0.083	0.166	0.102	0.552
	5	0.08	0.165	0.101	0.542
	10	0.078	0.160	0.092	0.511
	15	0.076	0.154	0.090	0.504
	20	0.07	0.151	0.079	0.493
	25	0.069	0.148	0.065	0.484
N3	0	0.088	0.166	0.097	0.534
	5	0.088	0.163	0.096	0.529
	10	0.085	0.160	0.095	0.526
	15	0.079	0.159	0.095	0.52
	20	0.075	0.158	0.084	0.498
	25	0.062	0.154	0.073	0.479
N4	0	0.086	0.166	0.097	0.557
	5	0.079	0.154	0.094	0.533
	10	0.071	0.151	0.092	0.516
	15	0.069	0.149	0.084	0.5
	20	0.068	0.148	0.082	0.496
	25	0.067	0.145	0.067	0.477
N5	0	0.085	0.168	0.102	0.555
	5	0.083	0.165	0.095	0.536
	10	0.082	0.162	0.094	0.527
	15	0.081	0.150	0.081	0.507
	20	0.074	0.149	0.080	0.498
	25	0.064	0.149	0.068	0.472
N6	0	0.084	0.168	0.097	0.542
	5	0.08	0.167	0.089	0.523
	10	0.076	0.162	0.085	0.518
	15	0.075	0.154	0.080	0.507
	20	0.073	0.147	0.075	0.482
	25	0.071	0.147	0.072	0.469
N7	0	0.088	0.166	0.095	0.54
	5	0.081	0.165	0.088	0.531
	10	0.077	0.161	0.071	0.517
	15	0.072	0.160	0.068	0.506
	20	0.071	0.152	0.066	0.473
	25	0.068	0.146	0.064	0.465
N8	0	0.084	0.167	0.101	0.552
	5	0.082	0.164	0.089	0.534
	10	0.08	0.154	0.076	0.531
	15	0.077	0.150	0.073	0.51
	20	0.075	0.148	0.066	0.49
	25	0.062	0.145	0.065	0.482
N9	0	0.088	0.159	0.102	0.554
	5	0.08	0.154	0.099	0.513
	10	0.075	0.154	0.083	0.501
	15	0.075	0.153	0.069	0.489
	20	0.063	0.151	0.066	0.486
	25	0.063	0.144	0.064	0.465
N10	0	0.089	0.167	0.099	0.546
	5	0.088	0.159	0.095	0.54
	10	0.081	0.154	0.089	0.49
	15	0.078	0.154	0.088	0.486
	20	0.077	0.150	0.080	0.48
	25	0.072	0.144	0.063	0.471
N11	0	0.082	0.168	0.095	0.543
	5	0.08	0.166	0.092	0.531
	10	0.077	0.162	0.082	0.526
	15	0.076	0.154	0.069	0.521
	20	0.076	0.153	0.067	0.514
	25	0.064	0.150	0.066	0.484
N12	0	0.08	0.164	0.097	0.554
	5	0.072	0.163	0.097	0.546
	10	0.072	0.155	0.080	0.546
	15	0.069	0.154	0.073	0.521
	20	0.067	0.149	0.073	0.466
	25	0.065	0.144	0.071	0.464

Table 30. The table shows the F-NC scores obtained by aligning the original synthetic network with its noisy versions when adding nodes for all the networks. Table shows a significant improvement of the quality of the alignments when considering networks with many colours. The improvement is also stable for all the networks.

Network	Altered Networks	F-NC on 1 coloured version	F-NC on 2 coloured version	F-NC on 3 coloured version	F-NC on 4 coloured version
N1	0	0.289	0.409	0.318	0.734
	5	0.289	0.402	0.312	0.723
	10	0.274	0.393	0.31	0.713
	15	0.256	0.389	0.305	0.705
	20	0.255	0.388	0.301	0.701
	25	0.253	0.386	0.27	0.701
N2	0	0.288	0.407	0.32	0.743
	5	0.282	0.406	0.318	0.736
	10	0.28	0.4	0.303	0.715
	15	0.276	0.393	0.3	0.71
	20	0.264	0.388	0.281	0.702
	25	0.262	0.385	0.255	0.696
N3	0	0.297	0.408	0.312	0.731
	5	0.296	0.404	0.31	0.727
	10	0.292	0.4	0.308	0.725
	15	0.281	0.399	0.308	0.721
	20	0.274	0.397	0.289	0.706
	25	0.25	0.392	0.271	0.692
N4	0	0.293	0.408	0.311	0.746
	5	0.281	0.393	0.307	0.73
	10	0.267	0.388	0.303	0.718
	15	0.263	0.386	0.289	0.707
	20	0.26	0.385	0.286	0.704
	25	0.259	0.381	0.258	0.691
N5	0	0.291	0.41	0.32	0.745
	5	0.288	0.406	0.309	0.732
	10	0.287	0.403	0.307	0.726
	15	0.285	0.387	0.285	0.712
	20	0.272	0.386	0.283	0.706
	25	0.252	0.386	0.26	0.687
N6	0	0.289	0.41	0.311	0.736
	5	0.282	0.409	0.299	0.723
	10	0.275	0.402	0.292	0.72
	15	0.274	0.392	0.282	0.712
	20	0.271	0.384	0.274	0.694
	25	0.266	0.383	0.268	0.685
N7	0	0.296	0.407	0.309	0.735
	5	0.285	0.406	0.297	0.729
	10	0.278	0.401	0.267	0.719
	15	0.268	0.4	0.26	0.711
	20	0.266	0.39	0.256	0.688
	25	0.26	0.382	0.253	0.682
N8	0	0.289	0.409	0.318	0.743
	5	0.286	0.405	0.298	0.731
	10	0.282	0.393	0.275	0.729
	15	0.277	0.387	0.27	0.714
	20	0.273	0.385	0.257	0.7
	25	0.25	0.381	0.254	0.694
N9	0	0.296	0.399	0.319	0.744
	5	0.283	0.393	0.314	0.716
	10	0.273	0.392	0.288	0.708
	15	0.273	0.391	0.263	0.699
	20	0.251	0.389	0.256	0.697
	25	0.251	0.38	0.253	0.682
N10	0	0.299	0.409	0.314	0.739
	5	0.296	0.399	0.309	0.735
	10	0.285	0.393	0.298	0.7
	15	0.28	0.392	0.296	0.697
	20	0.277	0.387	0.282	0.693
	25	0.269	0.38	0.251	0.686
N11	0	0.287	0.41	0.308	0.737
	5	0.282	0.407	0.303	0.729
	10	0.277	0.403	0.286	0.725
	15	0.276	0.392	0.262	0.722
	20	0.275	0.391	0.259	0.717
	25	0.252	0.387	0.257	0.696
N12	0	0.282	0.405	0.311	0.744
	5	0.269	0.404	0.311	0.739
	10	0.268	0.394	0.282	0.739
	15	0.263	0.392	0.271	0.722
	20	0.258	0.386	0.27	0.683
	25	0.255	0.38	0.267	0.681

Table 31. Table summarises the NCV-GS^3 scores obtained by aligning the original Hetionet network with its noisy versions. Altered networks are obtained by adding nodes randomly. Table shows a remarkable trend of improvements when considering networks with many colours for all the networks.

Network	Altered Networks	NCV-GS^3 on 1 coloured version	NCV-GS^3 on 2 coloured version	NCV-GS^3 on 3 coloured version	NCV-GS^3 on 4 coloured version
Hetionet	0% of noise	0.281	0.404	0.56	0.741
	5% of noise	0.278	0.4	0.556	0.727
	10% of noise	0.277	0.393	0.546	0.722
	15% of noise	0.275	0.391	0.544	0.7
	20% of noise	0.266	0.389	0.531	0.693
	25% of noise	0.263	0.386	0.501	0.682

Table 32. Table summarises the GS^3 scores obtained by aligning the original Hetionet network with its noisy versions when adding nodes. Table shows that the alignment quality is constant.

Network	Altered Networks	GS^3 on 1 coloured version	GS^3 on 2 coloured version	GS^3 on 3 coloured version	GS^3 on 4 coloured version
Hetionet	0% of noise	1	1	1	1
	5% of noise	1	1	1	1
	10% of noise	1	1	1	1
	15% of noise	1	1	1	1
	20% of noise	1	1	1	1
	25% of noise	1	1	1	1

Table 33. The table shows the NCV scores obtained by aligning the original synthetic network with its noisy versions when adding nodes for all the networks. Table shows a significant improvement of the quality of the alignments when considering networks with many colours. The improvement is also stable for all the networks.

Network	Altered Networks	NCV on 1 coloured version	NCV on 2 coloured version	NCV on 3 coloured version	NCV on 4 coloured version
Hetionet	0% of noise	0.079	0.163	0.358	0.549
	5% of noise	0.077	0.16	0.354	0.529
	10% of noise	0.077	0.154	0.353	0.521
	15% of noise	0.076	0.153	0.353	0.49
	20% of noise	0.071	0.151	0.341	0.48
	25% of noise	0.069	0.149	0.336	0.465

Table 34. Table shows the P-NC scores obtained by aligning the original Hetionet network with its noisy versions. Altered networks presents more nodes. Table shows that the alignment quality is constant.

Network	Altered Networks	P-NC on 1 coloured version	P-NC on 2 coloured version	P-NC on 3 coloured version	P-NC on 4 coloured version
Hetionet	0% of noise	1	1	1	1
	5% of noise	1	1	1	1
	10% of noise	1	1	1	1
	15% of noise	1	1	1	1
	20% of noise	1	1	1	1
	25% of noise	1	1	1	1

Table 35. The table shows the R-N scores obtained by aligning the original synthetic network with its noisy versions when adding nodes for all the networks. Table shows a significant improvement of the quality of the alignments when considering networks with many colours. The improvement is also stable for all the networks.

Network	Altered Networks	R-NC on 1 coloured version	R-NC on 2 coloured version	R-NC on 3 coloured version	R-NC on 4 coloured version
Hetionet	0% of noise	0.088	0.168	0.102	0.554
	5% of noise	0.088	0.167	0.099	0.546
	10% of noise	0.085	0.162	0.083	0.546
	15% of noise	0.079	0.154	0.069	0.521
	20% of noise	0.075	0.147	0.066	0.466
	25% of noise	0.062	0.147	0.064	0.464

Table 36. The table shows the F-NC scores obtained by aligning the original synthetic network with its noisy versions when adding nodes for all the networks. Table shows a significant improvement of the quality of the alignments when considering networks with many colours. The improvement is also stable for all the networks.

Network	Altered Networks	F-NC on 1 coloured version	F-NC on 2 coloured version	F-NC on 3 coloured version	F-NC on 4 coloured version
Hetionet	0% of noise	0.297	0.41	0.319	0.744
	5% of noise	0.296	0.409	0.314	0.739
	10% of noise	0.292	0.402	0.288	0.739
	15% of noise	0.281	0.392	0.263	0.722
	20% of noise	0.274	0.384	0.256	0.683
	25% of noise	0.25	0.383	0.253	0.681

Quality of the alignments synthetic heterogeneous networks obtained by aligning each network with its noisy versions built by removing a percentage of nodes randomly.

Table 37, Table 38, Table 39, Table 40, Table 41, Table 42 report the NCV-GS^3, GS^3, NCV, P-NC, R-NC, F-NC measures computed on each module for the four versions of synthetic heterogeneous networks obtained by aligning each network with its noisy versions built by removing a percentage of nodes.

Table 37. Table summarises the NCV-GS^3 scores obtained by aligning the original synthetic network with its noisy versions obtained by removing nodes. Table shows a significant improvement of the quality of the alignments when considering networks with many colours. The improvement is also stable for all the considered networks.

Network	Altered Networks	NCV-GS^3 on 1 coloured version	NCV-GS^3 on 2 coloured version	NCV-GS^3 on 3 coloured version	NCV-GS^3 on 4 coloured version
N1	0	0.339	0.468	0.6	0.817
	5	0.338	0.465	0.597	0.789
	10	0.333	0.46	0.597	0.78
	15	0.321	0.451	0.593	0.758
	20	0.317	0.442	0.584	0.756
	25	0.311	0.424	0.579	0.753
N2	0	0.348	0.46	0.599	0.819
	5	0.346	0.454	0.596	0.814
	10	0.345	0.448	0.592	0.805
	15	0.341	0.446	0.581	0.804
	20	0.326	0.44	0.581	0.778
	25	0.307	0.439	0.571	0.774
N3	0	0.342	0.466	0.596	0.812
	5	0.336	0.465	0.585	0.789
	10	0.333	0.461	0.584	0.778
	15	0.324	0.452	0.581	0.778
	20	0.318	0.449	0.58	0.768
	25	0.314	0.434	0.576	0.754
N4	0	0.347	0.469	0.6	0.807
	5	0.321	0.461	0.6	0.806
	10	0.314	0.459	0.594	0.79
	15	0.311	0.449	0.582	0.786
	20	0.31	0.437	0.581	0.772
	25	0.302	0.431	0.58	0.762
N5	0	0.348	0.465	0.598	0.816
	5	0.346	0.461	0.593	0.799
	10	0.342	0.457	0.585	0.787
	15	0.325	0.455	0.585	0.777
	20	0.308	0.45	0.577	0.774
	25	0.306	0.437	0.577	0.768
N6	0	0.342	0.461	0.601	0.814
	5	0.337	0.458	0.6	0.814
	10	0.331	0.448	0.59	0.811
	15	0.33	0.444	0.589	0.788
	20	0.309	0.442	0.588	0.778
	25	0.301	0.429	0.572	0.766
N7	0	0.349	0.464	0.598	0.812
	5	0.349	0.462	0.587	0.798
	10	0.342	0.461	0.581	0.79
	15	0.34	0.443	0.581	0.79
	20	0.316	0.442	0.58	0.787
	25	0.309	0.435	0.577	0.765
N8	0	0.336	0.469	0.6	0.799
	5	0.329	0.464	0.591	0.797
	10	0.329	0.455	0.585	0.785
	15	0.307	0.448	0.583	0.785
	20	0.302	0.423	0.578	0.764
	25	0.301	0.421	0.571	0.755
N9	0	0.341	0.468	0.601	0.792
	5	0.333	0.454	0.601	0.789
	10	0.328	0.453	0.592	0.776
	15	0.327	0.446	0.592	0.76
	20	0.317	0.436	0.585	0.754
	25	0.301	0.43	0.571	0.75
N10	0	0.346	0.461	0.598	0.815
	5	0.33	0.453	0.595	0.814
	10	0.325	0.452	0.594	0.8
	15	0.319	0.441	0.594	0.796
	20	0.317	0.436	0.584	0.795
	25	0.313	0.424	0.58	0.764
N11	0	0.347	0.467	0.598	0.804
	5	0.335	0.466	0.587	0.785
	10	0.33	0.445	0.587	0.765
	15	0.329	0.444	0.579	0.758
	20	0.322	0.438	0.577	0.753
	25	0.303	0.429	0.57	0.752
N12	0	0.336	0.465	0.593	0.814
	5	0.336	0.462	0.591	0.803
	10	0.332	0.457	0.59	0.802
	15	0.328	0.453	0.581	0.783
	20	0.327	0.45	0.58	0.764
	25	0.3	0.446	0.571	0.761

Table 38. Table shows GS^3 scores obtained by aligning the original synthetic network with its noisy versions for all the networks. Table shows that the alignment quality is constant.

Network	Altered Networks	GS^3 on 1 coloured version	GS^3 on 2 coloured version	GS^3 on 3 coloured version	GS^3 on 4 coloured version
N1	0	1	1	1	1
	5	1	1	1	1
	10	1	1	1	1
	15	1	1	1	1
	20	1	1	1	1
	25	1	1	1	1
N2	0	1	1	1	1
	5	1	1	1	1
	10	1	1	1	1
	15	1	1	1	1
	20	1	1	1	1
	25	1	1	1	1
N3	0	1	1	1	1
	5	1	1	1	1
	10	1	1	1	1
	15	1	1	1	1
	20	1	1	1	1
	25	1	1	1	1
N4	0	1	1	1	1
	5	1	1	1	1
	10	1	1	1	1
	15	1	1	1	1
	20	1	1	1	1
	25	1	1	1	1
N5	0	1	1	1	1
	5	1	1	1	1
	10	1	1	1	1
	15	1	1	1	1
	20	1	1	1	1
	25	1	1	1	1
N6	0	1	1	1	1
	5	1	1	1	1
	10	1	1	1	1
	15	1	1	1	1
	20	1	1	1	1
	25	1	1	1	1
N7	0	1	1	1	1
	5	1	1	1	1
	10	1	1	1	1
	15	1	1	1	1
	20	1	1	1	1
	25	1	1	1	1
N8	0	1	1	1	1
	5	1	1	1	1
	10	1	1	1	1
	15	1	1	1	1
	20	1	1	1	1
	25	1	1	1	1
N9	0	1	1	1	1
	5	1	1	1	1
	10	1	1	1	1
	15	1	1	1	1
	20	1	1	1	1
	25	1	1	1	1
N10	0	1	1	1	1
	5	1	1	1	1
	10	1	1	1	1
	15	1	1	1	1
	20	1	1	1	1
	25	1	1	1	1
N11	0	1	1	1	1
	5	1	1	1	1
	10	1	1	1	1
	15	1	1	1	1
	20	1	1	1	1
	25	1	1	1	1
N12	0	1	1	1	1
	5	1	1	1	1
	10	1	1	1	1
	15	1	1	1	1
	20	1	1	1	1
	25	1	1	1	1

Quality of the alignments of Hetionet network obtained by aligning each network with its noisy versions built by removing a percentage of nodes

Table 43, Table 44, Table 45, Table 46, Table 47, Table 48 report the NCV-GS^3, GS^3, NCV, P-NC, R-NC, F-NC measures computed on each module for the four versions of the Hetionet network obtained by aligning each network with its noisy counterpart built by removing a percentage of nodes.

Experiments on different Network Models.

We generated the synthetic networks with different models to test the performances of our algorithm on different network structure. The aim of this experiment is to demonstrate the robustness of our approach on the change of network structure. Following results highlight that the algorithm has good performances in almost all the network models.

We built 5 synthetic networks having respectively 5000, 25000, 50000, 75000, 95000 nodes and a scale-free model. We built 5 synthetic networks having respectively 5000, 25000, 50000, 75000, 95000 nodes, and a geometric network model. We built 5 synthetic networks having respectively 5000, 25000, 50000, 75000, 95000 nodes using and a Erdos-Renyi network model. We built 5 synthetic networks having respectively 5000, 25000, 50000, 75000, 95000 nodes, and a small-world network model. The, we randomly assign each node a colour out of n possible colours. We vary n from one to four. That is, for each synthetic network, we built heterogeneous versions with one, two, three, and four colours. Then, we built the synthetic versions for all network models by random removing 5%, 10%, 15%, 20% and 25% of edges from the original network.

Then, we applied L-HetNetAligner to align the synthetic networks with its noisy versions. Finally, we compute NCV-GS^3 and F-NC measures for each synthetic network model.

Table 49 and Table 53 report the NCV-GS^3 and F-NC measures for all the network models in one colour version. Table 50 and Table 54 report the NCV-GS^3 and F-NC measures for all the network models in two colour versions. Table 51 and Table 55 report the NCV-GS^3 and F-NC measures for all the network models in three colour versions. Table 52 and Table 56 report the NCV-GS^3 and F-NC measures for all the network models in four colour versions. In terms of quality, we expect that for a given noise level, the more colours are used, the better the alignment quality should be. Moreover, the use of colours should also improve the robustness to noise compared to the use of fewer colours. The analysis of results shows that for a given level of noise the use of colours improves the quality of the alignment. Besides, the robustness to the impact of noise is better.

Table 39. Table summarises the NCV scores obtained by aligning the original synthetic network with its noisy versions obtained by removing nodes. Table shows a significant improvement of the quality of the alignments when considering networks with many colours. The improvement is also stable for all the considered networks.

Network	Altered Networks	NCV on 1 coloured version	NCV on 2 coloured version	NCV on 3 coloured version	NCV on 4 coloured version
N1	0	0.115	0.219	0.36	0.667
	5	0.114	0.216	0.356	0.623
	10	0.111	0.212	0.356	0.608
	15	0.103	0.203	0.352	0.575
	20	0.1	0.195	0.341	0.572
	25	0.097	0.18	0.335	0.567
N2	0	0.121	0.212	0.359	0.671
	5	0.12	0.206	0.355	0.663
	10	0.119	0.201	0.35	0.648
	15	0.116	0.199	0.338	0.646
	20	0.106	0.194	0.338	0.605
	25	0.094	0.193	0.326	0.599
N3	0	0.117	0.217	0.355	0.659
	5	0.113	0.216	0.342	0.623
	10	0.111	0.213	0.341	0.605
	15	0.105	0.204	0.338	0.605
	20	0.101	0.202	0.336	0.59
	25	0.099	0.188	0.332	0.569
N4	0	0.12	0.22	0.36	0.651
	5	0.103	0.213	0.36	0.65
	10	0.099	0.211	0.353	0.624
	15	0.097	0.202	0.339	0.618
	20	0.096	0.191	0.338	0.596
	25	0.091	0.186	0.336	0.581
N5	0	0.121	0.216	0.358	0.666
	5	0.12	0.213	0.352	0.638
	10	0.117	0.209	0.342	0.619
	15	0.106	0.207	0.342	0.604
	20	0.095	0.202	0.333	0.599
	25	0.094	0.191	0.333	0.59
N6	0	0.117	0.213	0.361	0.663
	5	0.114	0.21	0.36	0.663
	10	0.11	0.201	0.348	0.658
	15	0.109	0.197	0.347	0.621
	20	0.095	0.195	0.346	0.605
	25	0.091	0.184	0.327	0.587
N7	0	0.122	0.215	0.358	0.659
	5	0.122	0.213	0.345	0.637
	10	0.117	0.213	0.338	0.624
	15	0.116	0.196	0.338	0.624
	20	0.1	0.195	0.336	0.619
	25	0.095	0.189	0.333	0.585
N8	0	0.113	0.22	0.36	0.638
	5	0.108	0.215	0.349	0.635
	10	0.108	0.207	0.342	0.616
	15	0.094	0.201	0.34	0.616
	20	0.091	0.179	0.334	0.584
	25	0.091	0.177	0.326	0.57
N9	0	0.116	0.219	0.361	0.627
	5	0.111	0.206	0.361	0.623
	10	0.108	0.205	0.35	0.602
	15	0.107	0.199	0.35	0.578
	20	0.1	0.19	0.342	0.569
	25	0.091	0.185	0.326	0.562
N10	0	0.12	0.213	0.358	0.664
	5	0.109	0.205	0.354	0.663
	10	0.106	0.204	0.353	0.64
	15	0.102	0.194	0.353	0.634
	20	0.1	0.19	0.341	0.632
	25	0.098	0.18	0.336	0.584
N11	0	0.12	0.218	0.358	0.646
	5	0.112	0.217	0.345	0.616
	10	0.109	0.198	0.345	0.585
	15	0.108	0.197	0.335	0.575
	20	0.104	0.192	0.333	0.567
	25	0.092	0.184	0.325	0.566
N12	0	0.113	0.216	0.352	0.663
	5	0.113	0.213	0.349	0.645
	10	0.11	0.209	0.348	0.643
	15	0.108	0.205	0.338	0.613
	20	0.107	0.202	0.336	0.584
	25	0.09	0.199	0.326	0.579

Table 40. P-NC scores obtained by aligning the original synthetic network with its noisy versions for all the networks. Table shows that the alignment quality is constant.

Network	Altered Networks	P-NC on 1 coloured version	P-NC on 2 coloured version	P-NC on 3 coloured version	P-NC on 4 coloured version
N1	0<br>5<br>10<br>15<br>20<br>25	1<br>1<br>1<br>1<br>1<br>1	1<br>1<br>1<br>1<br>1<br>1	1<br>1<br>1<br>1<br>1<br>1	1<br>1<br>1<br>1<br>1<br>1
N2	0<br>5<br>10<br>15<br>20<br>25	1<br>1<br>1<br>1<br>1<br>1	1<br>1<br>1<br>1<br>1<br>1	1<br>1<br>1<br>1<br>1<br>1	1<br>1<br>1<br>1<br>1<br>1
N3	0<br>5<br>10<br>15<br>20<br>25	1<br>1<br>1<br>1<br>1<br>1	1<br>1<br>1<br>1<br>1<br>1	1<br>1<br>1<br>1<br>1<br>1	1<br>1<br>1<br>1<br>1<br>1
N4	0<br>5<br>10<br>15<br>20<br>25	1<br>1<br>1<br>1<br>1<br>1	1<br>1<br>1<br>1<br>1<br>1	1<br>1<br>1<br>1<br>1<br>1	1<br>1<br>1<br>1<br>1<br>1
N5	0<br>5<br>10<br>15<br>20<br>25	1<br>1<br>1<br>1<br>1<br>1	1<br>1<br>1<br>1<br>1<br>1	1<br>1<br>1<br>1<br>1<br>1	1<br>1<br>1<br>1<br>1<br>1
N6	0<br>5<br>10<br>15<br>20<br>25	1<br>1<br>1<br>1<br>1<br>1	1<br>1<br>1<br>1<br>1<br>1	1<br>1<br>1<br>1<br>1<br>1	1<br>1<br>1<br>1<br>1<br>1
N7	0<br>5<br>10<br>15<br>20<br>25	1<br>1<br>1<br>1<br>1<br>1	1<br>1<br>1<br>1<br>1<br>1	1<br>1<br>1<br>1<br>1<br>1	1<br>1<br>1<br>1<br>1<br>1
N8	0<br>5<br>10<br>15<br>20<br>25	1<br>1<br>1<br>1<br>1<br>1	1<br>1<br>1<br>1<br>1<br>1	1<br>1<br>1<br>1<br>1<br>1	1<br>1<br>1<br>1<br>1<br>1
N9	0<br>5<br>10<br>15<br>20<br>25	1<br>1<br>1<br>1<br>1<br>1	1<br>1<br>1<br>1<br>1<br>1	1<br>1<br>1<br>1<br>1<br>1	1<br>1<br>1<br>1<br>1<br>1
N10	0<br>5<br>10<br>15<br>20<br>25	1<br>1<br>1<br>1<br>1<br>1	1<br>1<br>1<br>1<br>1<br>1	1<br>1<br>1<br>1<br>1<br>1	1<br>1<br>1<br>1<br>1<br>1
N11	0<br>5<br>10<br>15<br>20<br>25	1<br>1<br>1<br>1<br>1<br>1	1<br>1<br>1<br>1<br>1<br>1	1<br>1<br>1<br>1<br>1<br>1	1<br>1<br>1<br>1<br>1<br>1
N12	0<br>5<br>10<br>15<br>20<br>25	1<br>1<br>1<br>1<br>1<br>1	1<br>1<br>1<br>1<br>1<br>1	1<br>1<br>1<br>1<br>1<br>1	1<br>1<br>1<br>1<br>1<br>1

Table 41. Table summarises the R-NC scores obtained by aligning the original synthetic network with its noisy versions obtained by removing nodes. Table shows a significant improvement of the quality of the alignments when considering networks with many colours. The improvement is also stable for all the considered networks.

Network	Altered Networks (percentage of nodes removed)	R-NC on 1 coloured version	R-NC on 2 coloured version	R-NC on 3 coloured version	R-NC on 4 coloured version
N1	0	0.106	0.193	0.323	0.545
	5	0.102	0.192	0.31	0.543
	10	0.089	0.185	0.304	0.542
	15	0.089	0.181	0.296	0.531
	20	0.088	0.166	0.279	0.516
	25	0.086	0.161	0.264	0.511
N2	0	0.111	0.202	0.334	0.54
	5	0.108	0.181	0.326	0.527
	10	0.097	0.171	0.318	0.524
	15	0.094	0.168	0.31	0.508
	20	0.09	0.165	0.298	0.506
	25	0.089	0.154	0.286	0.497
N3	0	0.108	0.176	0.345	0.546
	5	0.102	0.174	0.339	0.526
	10	0.1	0.167	0.308	0.514
	15	0.099	0.164	0.3	0.504
	20	0.094	0.162	0.294	0.5
	25	0.087	0.145	0.277	0.498
N4	0	0.112	0.202	0.336	0.539
	5	0.096	0.193	0.327	0.534
	10	0.091	0.18	0.317	0.516
	15	0.089	0.166	0.287	0.514
	20	0.088	0.15	0.277	0.501
	25	0.086	0.147	0.267	0.494
N5	0	0.113	0.196	0.345	0.523
	5	0.106	0.194	0.338	0.506
	10	0.105	0.187	0.32	0.501
	15	0.102	0.171	0.291	0.501
	20	0.102	0.154	0.278	0.5
	25	0.099	0.145	0.268	0.493
N6	0	0.112	0.202	0.339	0.536
	5	0.106	0.2	0.318	0.534
	10	0.106	0.193	0.314	0.529
	15	0.095	0.174	0.278	0.527
	20	0.093	0.167	0.278	0.521
	25	0.084	0.15	0.265	0.508
N7	0	0.1	0.201	0.339	0.539
	5	0.097	0.193	0.332	0.529
	10	0.095	0.175	0.325	0.52
	15	0.095	0.171	0.311	0.517
	20	0.094	0.165	0.303	0.498
	25	0.092	0.156	0.264	0.497
N8	0	0.106	0.184	0.332	0.539
	5	0.101	0.183	0.331	0.51
	10	0.099	0.176	0.295	0.506
	15	0.09	0.176	0.293	0.506
	20	0.086	0.166	0.286	0.503
	25	0.086	0.158	0.286	0.503
N9	0	0.115	0.185	0.336	0.543
	5	0.104	0.181	0.327	0.542
	10	0.104	0.175	0.294	0.54
	15	0.097	0.165	0.292	0.534
	20	0.092	0.156	0.283	0.531
	25	0.091	0.148	0.281	0.508
N10	0	0.116	0.202	0.333	0.531
	5	0.106	0.186	0.304	0.531
	10	0.106	0.176	0.284	0.529
	15	0.1	0.171	0.278	0.527
	20	0.092	0.158	0.277	0.524
	25	0.085	0.147	0.276	0.506
N11	0	0.11	0.198	0.334	0.537
	5	0.105	0.187	0.32	0.526
	10	0.104	0.171	0.304	0.516
	15	0.103	0.163	0.301	0.507
	20	0.1	0.151	0.297	0.501
	25	0.089	0.15	0.284	0.494
N12	0	0.104	0.19	0.338	0.536
	5	0.104	0.187	0.327	0.524
	10	0.097	0.181	0.31	0.521
	15	0.096	0.176	0.291	0.514
	20	0.09	0.161	0.269	0.514
	25	0.087	0.158	0.266	0.497

Table 42. Table summarises the F-NC scores obtained by aligning the original synthetic network with its noisy versions obtained by removing nodes. Table shows a significant improvement of the quality of the alignments when considering networks with many colours. The improvement is also stable for all the considered networks..

Network	Altered Networks (percentage of remotion)	F-NC on 1 coloured version	F-NC on 2 coloured version	F-NC on 3 coloured version	F-NC on 4 coloured version
N1	0	0.325	0.439	0.568	0.738
	5	0.32	0.438	0.557	0.737
	10	0.299	0.43	0.551	0.736
	15	0.298	0.426	0.544	0.729
	20	0.297	0.407	0.528	0.718
	25	0.293	0.401	0.514	0.715
N2	0	0.333	0.449	0.578	0.735
	5	0.329	0.425	0.571	0.726
	10	0.311	0.413	0.564	0.724
	15	0.306	0.41	0.557	0.713
	20	0.3	0.406	0.546	0.711
	25	0.298	0.392	0.535	0.705
N3	0	0.328	0.42	0.587	0.739
	5	0.319	0.417	0.582	0.725
	10	0.316	0.409	0.555	0.717
	15	0.314	0.405	0.548	0.71
	20	0.307	0.403	0.542	0.707
	25	0.295	0.381	0.526	0.706
N4	0	0.335	0.449	0.58	0.734
	5	0.31	0.439	0.572	0.731
	10	0.302	0.424	0.563	0.718
	15	0.299	0.407	0.536	0.717
	20	0.296	0.387	0.526	0.708
	25	0.294	0.384	0.517	0.703
N5	0	0.336	0.443	0.587	0.723
	5	0.325	0.44	0.581	0.711
	10	0.324	0.433	0.566	0.708
	15	0.32	0.414	0.539	0.708
	20	0.319	0.392	0.527	0.707
	25	0.315	0.381	0.518	0.702
N6	0	0.335	0.449	0.582	0.732
	5	0.326	0.447	0.564	0.731
	10	0.325	0.439	0.56	0.727
	15	0.309	0.417	0.527	0.726
	20	0.305	0.409	0.527	0.722
	25	0.29	0.387	0.515	0.713
N7	0	0.316	0.448	0.582	0.734
	5	0.311	0.439	0.576	0.727
	10	0.308	0.418	0.57	0.721
	15	0.308	0.413	0.558	0.719
	20	0.306	0.406	0.55	0.706
	25	0.304	0.395	0.514	0.705
N8	0	0.326	0.429	0.576	0.734
	5	0.318	0.428	0.575	0.714
	10	0.315	0.42	0.543	0.711
	15	0.3	0.419	0.541	0.711
	20	0.294	0.407	0.535	0.709
	25	0.294	0.397	0.535	0.709
N9	0	0.339	0.43	0.58	0.737
	5	0.323	0.426	0.572	0.736
	10	0.323	0.418	0.542	0.735
	15	0.311	0.406	0.54	0.731
	20	0.303	0.395	0.532	0.729
	25	0.302	0.385	0.53	0.713
N10	0	0.34	0.449	0.577	0.729
	5	0.326	0.431	0.551	0.729
	10	0.326	0.419	0.533	0.727
	15	0.316	0.414	0.527	0.726
	20	0.304	0.398	0.526	0.724
	25	0.292	0.383	0.525	0.711
N11	0	0.331	0.445	0.578	0.733
	5	0.324	0.432	0.566	0.725
	10	0.322	0.413	0.551	0.718
	15	0.321	0.404	0.549	0.712
	20	0.317	0.388	0.545	0.708
	25	0.299	0.387	0.533	0.703
N12	0	0.323	0.436	0.581	0.732
	5	0.322	0.433	0.572	0.724
	10	0.312	0.425	0.557	0.722
	15	0.31	0.42	0.539	0.717
	20	0.3	0.401	0.519	0.717
	25	0.295	0.397	0.516	0.705

Table 43. NCV-GS^3 scores obtained by aligning the original Hetionet network with its noisy versions (removing nodes). Table shows a remarkable trend of improvements when considering networks with many colours for all the networks.

Network	Altered Networks	NCV-GS^3 on 1 coloured version	NCV-GS^3 on 2 coloured version	NCV-GS^3 on 3 coloured version	NCV-GS^3 on 4 coloured version
Hetionet	0% of noise	0.347	0.464	0.598	0.817
	5% of noise	0.321	0.462	0.593	0.789
	10% of noise	0.314	0.461	0.585	0.78
	15% of noise	0.311	0.443	0.585	0.758
	20% of noise	0.31	0.442	0.577	0.756
	25% of noise	0.302	0.435	0.577	0.753

Table 44. GS^3 scores obtained by aligning the original Hetionet network with its noisy versions (removing nodes). Table shows that the alignment quality is constant when considering networks with many colours.

Network	Altered Networks	GS^3 on 1 coloured version	GS^3 on 2 coloured version	GS^3 on 3 coloured version	GS^3 on 4 coloured version
Hetionet	0% of noise	1	1	1	1
	5% of noise	1	1	1	1
	10% of noise	1	1	1	1
	15% of noise	1	1	1	1
	20% of noise	1	1	1	1
	25% of noise	1	1	1	1

Table 45. Table shows the NCV scores obtained by aligning the original Hetionet network with its noisy versions when removing nodes. Table shows a remarkable trend of improvement when considering networks with many colours for all the networks.

Network	Altered Networks (percentage of remotion)	NCV on 1 coloured version	NCV on 2 coloured version	NCV on 3 coloured version	NCV on 4 coloured version
Hetionet	0% of noise	0.12	0.215	0.358	0.667
	5% of noise	0.103	0.213	0.352	0.623
	10% of noise	0.099	0.213	0.342	0.608
	15% of noise	0.097	0.196	0.342	0.575
	20% of noise	0.096	0.195	0.333	0.572
	25% of noise	0.091	0.189	0.333	0.567

Table 46. Table shows the P-NC scores obtained by aligning the original Hetionet network with its noisy versions (when removing nodes). Table shows that the alignment quality is constant by considering both un-coloured and more colours networks.

Network	Altered Networks (percentage of remotion)	P-NC on 1 coloured version	P-NC on 2 coloured version	P-NC on 3 coloured version	P-NC on 4 coloured version
Hetionet	0% of noise	1	1	1	1
	5% of noise	1	1	1	1
	10% of noise	1	1	1	1
	15% of noise	1	1	1	1
	20% of noise	1	1	1	1
	25% of noise	1	1	1	1

Table 47. Table shows the R-NC scores obtained by aligning the original Hetionet network with its noisy versions when removing nodes. Table shows a remarkable trend of improvements when considering networks with many colours for all the networks.

Network	Altered Networks (percentage of remotion)	R-NC on 1 coloured version	R-NC on 2 coloured version	R-NC on 3 coloured version	R-NC on 4 coloured version
Hetionet	0% of noise	0.12	0.215	0.358	0.667
	5% of noise	0.103	0.213	0.352	0.623
	10% of noise	0.099	0.213	0.342	0.608
	15% of noise	0.097	0.196	0.342	0.575
	20% of noise	0.096	0.195	0.333	0.572
	25% of noise	0.091	0.189	0.333	0.567

Table 48. Table shows the F-NC scores obtained by aligning the original Hetionet network with its noisy versions when removing nodes. Table shows a remarkable trend of improvements when considering networks with many colours for all the networks.

Network	Altered Networks (percentage of remotion)	F-NC on 1 coloured version	F-NC on 2 coloured version	F-NC on 3 coloured version	F-NC on 4 coloured version
Hetionet	0% of noise	0.335	0.449	0.581	0.734
	5% of noise	0.326	0.431	0.572	0.727
	10% of noise	0.325	0.419	0.557	0.721
	15% of noise	0.309	0.414	0.539	0.719
	20% of noise	0.305	0.398	0.519	0.706
	25% of noise	0.29	0.383	0.516	0.705

Table 49. Table shows the NCV-GS^3 scores obtained by aligning the original synthetic network with its noisy versions when removing edges for all the network models having a single colour.

Model	Altered Networks (percentage of remotion)	5000 nodes	25000 nodes	50000 nodes	75000 nodes	95000 nodes
Scale-free	0	0.468	0.478	0.498	0.51	0.529
	5	0.467	0.477	0.497	0.509	0.528
	10	0.466	0.475	0.491	0.509	0.524
	15	0.466	0.474	0.491	0.507	0.521
	20	0.463	0.473	0.488	0.506	0.516
	25	0.461	0.473	0.486	0.504	0.514
Geometric	0	0.468	0.484	0.499	0.517	0.544
	5	0.467	0.484	0.498	0.515	0.543
	10	0.464	0.482	0.497	0.515	0.542
	15	0.464	0.482	0.496	0.514	0.541
	20	0.462	0.481	0.494	0.513	0.539
	25	0.461	0.48	0.491	0.51	0.539
Erdos-Renyi	0	0.466	0.479	0.495	0.507	0.53
	5	0.462	0.474	0.494	0.507	0.529
	10	0.461	0.473	0.494	0.506	0.529
	15	0.459	0.473	0.492	0.505	0.527
	20	0.453	0.472	0.488	0.504	0.527
	25	0.451	0.472	0.486	0.502	0.521
Small-world	0	0.469	0.478	0.497	0.51	0.528
	5	0.467	0.478	0.491	0.509	0.528
	10	0.458	0.474	0.491	0.505	0.527
	15	0.457	0.474	0.491	0.504	0.526
	20	0.454	0.471	0.486	0.504	0.526
	25	0.454	0.471	0.485	0.503	0.52

Table 50. Table shows the NCV-GS^3 scores obtained by aligning the original synthetic network with its noisy versions. Table presents two colored networks for all the network models.

Model	Altered Networks (percentage of edge remotion)	5000 nodes	25000 nodes	50000 nodes	75000 nodes	95000 nodes
Scale-free	0	0.656	0.682	0.728	0.764	0.81
	5	0.637	0.682	0.723	0.76	0.809
	10	0.63	0.681	0.722	0.752	0.802
	15	0.624	0.68	0.718	0.75	0.8
	20	0.621	0.678	0.716	0.743	0.785
	25	0.607	0.678	0.702	0.732	0.773
Geometric	0	0.658	0.68	0.724	0.764	0.811
	5	0.65	0.68	0.723	0.756	0.792
	10	0.649	0.675	0.72	0.755	0.781
	15	0.646	0.673	0.717	0.753	0.779
	20	0.632	0.673	0.706	0.744	0.775
	25	0.631	0.672	0.705	0.737	0.775
Erdos-Renyi	0	0.663	0.684	0.713	0.768	0.811
	5	0.658	0.684	0.71	0.76	0.808
	10	0.643	0.684	0.708	0.755	0.8
	15	0.626	0.677	0.706	0.752	0.797
	20	0.615	0.675	0.702	0.75	0.795
	25	0.612	0.671	0.702	0.748	0.783
Small-world	0	0.666	0.679	0.726	0.769	0.803
	5	0.641	0.678	0.723	0.763	0.793
	10	0.641	0.677	0.721	0.749	0.787
	15	0.64	0.677	0.72	0.745	0.787
	20	0.62	0.676	0.719	0.744	0.785
	25	0.616	0.674	0.705	0.735	0.779

Table 51. Table shows the NCV-GS^3 scores obtained by aligning the original synthetic network with three colors with its noisy versions when removing edges for all the network models.

Model	Altered Networks (Percentage of remotion)	5000 nodes	25000 nodes	50000 nodes	75000 nodes	95000 nodes
Scale-free	0	0.818	0.828	0.834	0.84	0.883
	5	0.817	0.825	0.833	0.84	0.883
	10	0.816	0.824	0.833	0.839	0.883
	15	0.815	0.823	0.833	0.839	0.871
	20	0.813	0.822	0.832	0.838	0.865
	25	0.813	0.822	0.831	0.837	0.843
Geometric	0	0.82	0.828	0.834	0.84	0.882
	5	0.819	0.827	0.834	0.839	0.879
	10	0.818	0.826	0.833	0.839	0.869
	15	0.816	0.824	0.833	0.837	0.848
	20	0.815	0.823	0.832	0.837	0.847
	25	0.813	0.822	0.831	0.836	0.844
Erdos-Renyi	0	0.819	0.827	0.833	0.839	0.869
	5	0.818	0.825	0.832	0.839	0.866
	10	0.816	0.825	0.831	0.838	0.855
	15	0.815	0.825	0.831	0.838	0.855
	20	0.814	0.824	0.83	0.838	0.851
	25	0.813	0.822	0.83	0.837	0.847
Small-world	0	0.819	0.827	0.834	0.839	0.871
	5	0.818	0.826	0.834	0.838	0.863
	10	0.818	0.826	0.832	0.838	0.855
	15	0.817	0.825	0.832	0.837	0.848
	20	0.814	0.823	0.832	0.837	0.846
	25	0.814	0.822	0.83	0.836	0.843

Table 52. Table summarises the NCV-GS^3 scores obtained by aligning the original synthetic network with four colours with its noisy versions when removing edges for all the network models.

Model	Altered Networks (percentage of remotion)	5000 nodes	25000 nodes	50000 nodes	75000 nodes	95000 nodes
Scale-free	0	0.898	0.898	0.939	0.948	0.997
	5	0.898	0.898	0.939	0.947	0.988
	10	0.898	0.898	0.939	0.946	0.986
	15	0.888	0.888	0.937	0.945	0.979
	20	0.887	0.887	0.936	0.945	0.976
	25	0.885	0.885	0.932	0.941	0.953
Geometric	0	0.898	0.898	0.936	0.951	0.992
	5	0.898	0.898	0.933	0.948	0.991
	10	0.895	0.895	0.932	0.947	0.99
	15	0.894	0.894	0.93	0.946	0.984
	20	0.889	0.889	0.93	0.945	0.981
	25	0.889	0.889	0.93	0.944	0.967
Erdos-Renyi	0	0.898	0.898	0.936	0.952	0.995
	5	0.892	0.892	0.935	0.95	0.994
	10	0.892	0.892	0.935	0.948	0.993
	15	0.891	0.891	0.934	0.945	0.975
	20	0.887	0.887	0.931	0.943	0.962
	25	0.885	0.885	0.93	0.942	0.959
Small-world	0	0.899	0.899	0.938	0.948	0.993
	5	0.896	0.896	0.936	0.948	0.988
	10	0.895	0.895	0.936	0.947	0.985
	15	0.895	0.895	0.936	0.946	0.97
	20	0.889	0.889	0.934	0.945	0.96
	25	0.884	0.884	0.931	0.942	0.955

Table 53. Table shows the F-NC scores obtained by aligning the original synthetic network with one color with four colours with its noisy versions when removing edges for all the network models.

Model	Altered Networks (percentage of remotion)	5000 nodes	25000 nodes	50000 nodes	75000 nodes	95000 nodes
Scale-free	0	0.609	0.628	0.649	0.69	0.706
	5	0.608	0.627	0.648	0.688	0.704
	10	0.606	0.626	0.647	0.685	0.699
	15	0.606	0.625	0.646	0.684	0.698
	20	0.604	0.624	0.645	0.683	0.695
	25	0.604	0.624	0.644	0.681	0.695
Geometric	0	0.609	0.629	0.648	0.689	0.811
	5	0.606	0.628	0.648	0.687	0.792
	10	0.606	0.626	0.648	0.686	0.781
	15	0.605	0.626	0.644	0.685	0.779
	20	0.605	0.626	0.643	0.685	0.775
	25	0.603	0.624	0.642	0.684	0.775
Erdos-Renyi	0	0.607	0.629	0.649	0.689	0.811
	5	0.607	0.628	0.649	0.689	0.808
	10	0.607	0.626	0.647	0.688	0.8
	15	0.606	0.623	0.645	0.687	0.797
	20	0.606	0.623	0.641	0.686	0.795
	25	0.601	0.622	0.64	0.682	0.783
Small-world	0	0.61	0.63	0.65	0.69	0.803
	5	0.609	0.628	0.647	0.689	0.793
	10	0.608	0.628	0.647	0.689	0.787
	15	0.608	0.627	0.646	0.687	0.787
	20	0.602	0.626	0.643	0.685	0.785
	25	0.601	0.623	0.642	0.683	0.779

Table 54. Table shows the F-NC scores obtained by aligning the original synthetic network with two colors and its noisy versions for all the network models.

Model	Altered Networks (Percentage of Noise)	5000 nodes	25000 nodes	50000 nodes	75000 nodes	95000 nodes
Scale-free	0	0.609	0.628	0.649	0.69	0.706
	5	0.608	0.627	0.648	0.688	0.704
	10	0.606	0.626	0.647	0.685	0.699
	15	0.606	0.625	0.646	0.684	0.698
	20	0.604	0.624	0.645	0.683	0.695
	25	0.604	0.624	0.644	0.681	0.695
Geometric	0	0.609	0.629	0.648	0.689	0.811
	5	0.606	0.628	0.648	0.687	0.792
	10	0.606	0.626	0.648	0.686	0.781
	15	0.605	0.626	0.644	0.685	0.779
	20	0.605	0.626	0.643	0.685	0.775
	25	0.603	0.624	0.642	0.684	0.775
Erdos-Renyi	0	0.607	0.629	0.649	0.689	0.811
	5	0.607	0.628	0.649	0.689	0.808
	10	0.607	0.626	0.647	0.688	0.8
	15	0.606	0.623	0.645	0.687	0.797
	20	0.606	0.623	0.641	0.686	0.795
	25	0.601	0.622	0.64	0.682	0.783
Small-world	0	0.61	0.63	0.65	0.69	0.803
	5	0.609	0.628	0.647	0.689	0.793
	10	0.608	0.628	0.647	0.689	0.787
	15	0.608	0.627	0.646	0.687	0.787
	20	0.602	0.626	0.643	0.685	0.785
	25	0.601	0.623	0.642	0.683	0.779

Table 55. Table shows F-NC scores obtained by aligning the original synthetic network with three colours and its noisy versions when removing edges for all the network models.

Model	Altered Networks (Percentage of removed edges)	5000 nodes	25000 nodes	50000 nodes	75000 nodes	95000 nodes
Scale-free	0	0.729	0.75	0.754	0.765	0.841
	5	0.727	0.75	0.753	0.764	0.835
	10	0.725	0.743	0.751	0.762	0.798
	15	0.719	0.742	0.751	0.76	0.783
	20	0.717	0.74	0.748	0.759	0.762
	25	0.711	0.735	0.747	0.756	0.76
Geometric	0	0.727	0.748	0.754	0.766	0.824
	5	0.724	0.743	0.752	0.764	0.82
	10	0.719	0.737	0.752	0.762	0.804
	15	0.717	0.737	0.75	0.76	0.782
	20	0.717	0.735	0.747	0.759	0.775
	25	0.714	0.732	0.743	0.757	0.77
Erdos-Renyi	0	0.729	0.749	0.752	0.765	0.823
	5	0.726	0.744	0.751	0.764	0.822
	10	0.72	0.741	0.751	0.764	0.802
	15	0.718	0.738	0.749	0.762	0.783
	20	0.717	0.736	0.746	0.761	0.778
	25	0.717	0.732	0.743	0.76	0.769
Small-world	0	0.729	0.75	0.755	0.763	0.834
	5	0.728	0.75	0.748	0.76	0.82
	10	0.725	0.74	0.747	0.759	0.812
	15	0.72	0.74	0.747	0.759	0.781
	20	0.717	0.739	0.744	0.759	0.78
	25	0.715	0.732	0.743	0.758	0.772

Table 56. Table shows the F-NC scores obtained by aligning the original synthetic network with four colours and its noisy versions when removing edges for all the network models.

**Model**	**Altered Networks (percentage of removed edges)**	**5000 nodes**	**25000 nodes**	**50000 nodes**	**75000 nodes**	**95000 nodes**
Scale-free	0	0.849	0.857	0.873	0.917	0.98
	5	0.847	0.857	0.866	0.91	0.964
	10	0.847	0.857	0.865	0.908	0.957
	15	0.846	0.854	0.857	0.905	0.953
	20	0.846	0.853	0.855	0.904	0.943
	25	0.844	0.85	0.854	0.904	0.904
Geometric	0	0.847	0.86	0.87	0.919	0.975
	5	0.847	0.858	0.866	0.917	0.966
	10	0.846	0.857	0.862	0.912	0.965
	15	0.846	0.856	0.862	0.911	0.959
	20	0.845	0.855	0.854	0.904	0.956
	25	0.845	0.855	0.852	0.903	0.954
Erdos-Renyi	0	0.849	0.856	0.875	0.92	0.957
	5	0.848	0.856	0.87	0.913	0.955
	10	0.847	0.854	0.867	0.91	0.954
	15	0.845	0.852	0.867	0.908	0.953
	20	0.844	0.852	0.865	0.905	0.947
	25	0.843	0.851	0.861	0.903	0.945
Small-world	0	0.849	0.857	0.874	0.92	0.974
	5	0.848	0.857	0.871	0.916	0.969
	10	0.847	0.856	0.868	0.914	0.968
	15	0.847	0.854	0.867	0.913	0.967
	20	0.846	0.853	0.858	0.906	0.963
	25	0.845	0.852	0.857	0.903	0.957

Table 57. NCV-GS^3 scores obtained by aligning the original network with its noisy versions (edges removing). Table shows a remarkable trend of improvements when considering networks with many colours for all the networks.

Network	Altered Networks	1 colour	2 colour	3 colour	4 colour	5 colour	6 colour	7 colour	8 colour
N	0% of noise	0.418	0.533	0.665	0.704	0.813	0.867	0.898	1
	5% of noise	0.413	0.53	0.647	0.703	0.803	0.856	0.898	0.996
	10% of noise	0.411	0.53	0.632	0.701	0.774	0.854	0.897	0.986
	15% of noise	0.411	0.526	0.629	0.701	0.763	0.843	0.897	0.982
	20% of noise	0.401	0.519	0.625	0.699	0.76	0.834	0.888	0.98
	25% of noise	0.392	0.518	0.602	0.698	0.745	0.825	0.88	0.963

Synthetic Networks with eight colors.

The input dataset consists of a synthetic networks built using scale-free networks (SF) graph generator. The network has 950 nodes and 4124. Then, we assign each node a colour out of n possible colours. We vary n from 1 to 8 in order to build four heterogeneous versions for each synthetic network as follows:

- 1 coloured version;
- 2 coloured version (in which 580 nodes present one colour and 382 nodes have another colour);
- 3 coloured version where we randomly assign one colour to 358 nodes, a second colour to 256 nodes and a third colour to 336 nodes;
- 4 coloured version where we randomly assign one colour to 170 nodes, a second colour to 288 nodes, a third colour to 192 nodes and a fourth to 300 nodes.
- 5 coloured version where we randomly assign one colour to 110 nodes, a second colour to 210 nodes, a third colour to 157 nodes, a fourth colour to 314 nodes and a fifth to 159 node;
- 6 coloured version where we randomly assign one colour to 98 nodes, a second colour to 124 nodes, a third colour to 242 nodes, a fourth colour to 103 nodes, a fifth colour to 211 nodes and sixth to 172 nodes;
- 7 coloured version where we randomly assign one colour to 110 nodes, a second colour to 124 nodes, a third colour to 170 nodes, a fourth colour to 223 nodes, a fifth colour to 94 nodes, a sixth to 115 nodes and seventh to 114 nodes;
- 8 coloured version where we randomly assign one colour to 110 nodes, a second colour to 94 nodes, a third colour to 121 nodes, a fourth to 97 nodes, a fifth colour to 128 nodes, a sixth to 100 nodes, seventh colour to 130 nodes and eighth to 170 nodes.

We built the synthetic versions by random removing 5%, 10%, 15%, 20% and 25% of edges from the original network.

Then, we applied L-HetNetAligner to align the synthetic network with its noisy versions. Finally, we compute NCV-GS^3 and F-NC measures for each synthetic network.

Table 57 and Table 58 report the NCV-GS^3 and F-NC the measures related to the alignment of the original synthetic network with its versions at 0%, 5%, 10%, 15%, 20% and 25% of added noise for all synthetic networks.

In terms of quality, we expect that for a given noise level, the more colours are used, the better the alignment quality should be. Moreover, the use of colours should also improve the robustness to noise compared to the use of fewer colours. The analysis of results shows that for a given level of noise the use of colours improves the quality of the alignment. Besides, the robustness to the impact of noise is better.

Table 58. F-NC scores obtained by aligning the original network with its noisy versions (edges removing). Table shows a remarkable trend of improvements when considering networks with many colours for all the networks.

Network	Altered Networks	1 colour	2 colour	3 colour	4 colour	5 colour	6 colour	7 colour	8 colour
N	0% of noise	0.412	0.558	0.664	0.705	0.725	0.839	0.877	0.976
	5% of noise	0.412	0.551	0.654	0.705	0.724	0.831	0.894	0.935
	10% of noise	0.41	0.542	0.622	0.704	0.705	0.818	0.893	0.935
	15% of noise	0.407	0.527	0.62	0.703	0.703	0.814	0.885	0.925
	20% of noise	0.397	0.475	0.617	0.695	0.702	0.785	0.882	0.907
	25% of noise	0.393	0.473	0.615	0.694	0.701	0.775	0.879	0.904

Comparison with respect to Single Color Alignments.

To demonstrate the effectiveness of L-HetNaligner we want to test if our algorithm is able to obtain better results when we apply it on the original network than on different subnetworks obtained from this one.

We considered two cases 1) synthetic networks, and 2) Hetionet network.

We consider the same synthetic network with 950 nodes and 3410 edges and four node colors. Then we split this network into four network considering nodes of the same color.

We obtain the **subnetwork 1** has 170 nodes and 474 edges, the **subnetwork 2** has 250 nodes and 422 edges, the **subnetwork 3** has 330 nodes and 528 edges, and the **subnetwork 4** has 220 nodes and 404 edges. Please note that the sum of the edges of the four subnetworks is lower than the number of the initial network since all the *cross*-edges, i.e. edges among node of different colors, have been removed.

We select the Hetionet network (with 37142 nodes and 6014211 edges) in four coloured version and from it we created four subnetwork according to four node types (i.e. colours.) According to this, the subnetwork 1 has 2095 nodes and 26567 edges, subnetwork 2 has 136 nodes and 543 edges, subnetwork 3 has 405 nodes and 742 edges, subnetwork 15056 has 78234 nodes and 404 edges.

Then, we built the synthetic versions for the synthetic network and Hetionet network and their subnetworks by random removing 5%, 10%, 15%, 20% and 25% of edges from the original network.

Then, we applied L-HetNetAligner to align the synthetic networks and Hetionet network with their sub networks with their noisy versions. Then, we compute NCV-GS^3 and F-NC measures for the synthetic network and Hetionet network and for the sub networks. Finally, we tested the ability of our algorithm to infer missing links from input networks (link prediction) by count how many homogeneous and heterogeneous gaps are found in the alignment graph of the synthetic network and Hetionet network and for their sub networks.

Table 59 and Table 60 report NCV-GS^3 and F-NC scores the synthetic network and its four subnetwork and Hetionet network and its four subnetwork. As evident the NCV-GS^3 and F-NC values in original network outperform NCV-GS^3 and F-NC values in subnetworks for both synthetic network and Hetionet network.

Table 59. NCV-GS^3 and F-NC scores obtained by aligning the synthetic network with its noisy versions and the four subnetworks with their noisy versions.

Network	Altered Networks	NCV-GS^3	F-NC
	0% of noise	0.99	0.98
	5% of noise	0.989	0.964
Synthetic	10% of noise	0.983	0.957
	15% of noise	0.978	0.953
	20% of noise	0.96	0.943
	25% of noise	0.957	0.904
	0% of noise	0.687	0.619
	5% of noise	0.68	0.619
subn 1	10% of noise	0.677	0.616
	15% of noise	0.677	0.606
	20% of noise	0.664	0.604
	25% of noise	0.663	0.604
	0% of noise	0.697	0.633
	5% of noise	0.696	0.627
subn 2	10% of noise	0.694	0.619
	15% of noise	0.662	0.609
	20% of noise	0.657	0.606
	25% of noise	0.655	0.606
	0% of noise	0.702	0.631
	5% of noise	0.696	0.629
subn 3	10% of noise	0.689	0.629
	15% of noise	0.672	0.625
	20% of noise	0.665	0.623
	25% of noise	0.657	0.61
	0% of noise	0.694	0.63
	5% of noise	0.69	0.625
subn 4	10% of noise	0.685	0.625
	15% of noise	0.665	0.611
	20% of noise	0.658	0.608
	25% of noise	0.656	0.602

Table 60. NCV-GS^3 and F-NC scores obtained by aligning the Hetionet network with its noisy versions and the four subnetworks with their noisy versions .

Network	Altered Networks	NCV-GS^3	F-NC
Hetionet	0% of noise	1	0.989
	5% of noise	0.996	0.961
	10% of noise	0.996	0.952
	15% of noise	0.966	0.943
	20% of noise	0.954	0.923
	25% of noise	0.951	0.912
subn 1	0% of noise	0.623	0.607
	5% of noise	0.623	0.601
	10% of noise	0.618	0.597
	15% of noise	0.601	0.583
	20% of noise	0.595	0.581
	25% of noise	0.592	0.57
subn 2	0% of noise	0.612	0.611
	5% of noise	0.606	0.608
	10% of noise	0.604	0.605
	15% of noise	0.604	0.601
	20% of noise	0.593	0.598
	25% of noise	0.59	0.567
subn 3	0% of noise	0.612	0.611
	5% of noise	0.606	0.609
	10% of noise	0.595	0.591
	15% of noise	0.594	0.576
	20% of noise	0.589	0.571
	25% of noise	0.588	0.57
subn 4	0% of noise	0.621	0.609
	5% of noise	0.62	0.592
	10% of noise	0.612	0.588
	15% of noise	0.606	0.586
	20% of noise	0.605	0.576
	25% of noise	0.601	0.568

Predicted Links Missed by Single Color Alignments

Table 61 reports the number of correctly predicted links obtained by aligning original synthetic network and it subnetworks with its noisy versions obtained by random removal of pair-matched nodes for all the networks. Table 62 reports the number of correctly predicted links obtained by aligning Hetionet network and it subnetworks with its noisy versions obtained by random removal of pair-matched nodes for all the networks.

We should note L-HetNetAligner can predict a high number of link for the synthetic network respect to its sub networks and for Hetionet respect to its sub networks.

Finally, we reported an examples of predicted link in Hetionet networks:

- Metaedge **Anatomy::UBERON:0000955-Gene::3892** is missing in one coloured version and it is predicted in two, three and four colour versions;
- Metaedges **Gene::3725- Molecular Function::GO:0070412, Gene::6259-Biological Process::GO:0032990, Anatomy::UBERON:0002030-Gene::79944** are predicted only four colour versions.
- Metaedges **Gene::9585-Molecular Function::GO:0017111, Gene::3433 -Gene::3875, Anatomy::UBERON:0002037-Gene::56288** is missing in one coloured version and it is predicted in two, three and four colour versions;
- Metaedges **Disease::DOID:3393-Gene::28, Gene::25-Biological Process::GO:1903530, Anatomy::UBERON:0000473-Gene::124961, Gene::51111 Molecular Function::GO:0016278, Disease::DOID:1793 Gene::4893** is missing in one and two coloured version and it is predicted in three and four colour versions;
- Metaedges **Disease::DOID:3393-Gene::28, Gene::25-Biological Process::GO:1903530, Anatomy::UBERON:0000473-Gene::124961, Gene::51111 Molecular Function::GO:0016278, Disease::DOID:1793 Gene::4893, Disease::DOID:0050156-Gene::664, Gene::3385-Molecular Function::GO:0005178, Gene::57835-Molecular Function::GO:0015081, Anatomy::UBERON:0002240-Gene::81502, Anatomy::UBERON:0001044-Gene::80790, Anatomy::UBERON:0000473-Gene::9778, Gene::2624-Biological Process::GO:0030100, Gene::7903-Biological Process::GO:0006664, Gene::64220-Biological Process::GO:0048592, Gene::7321-Gene::84231, Gene::6737-Gene::8202, Gene::10987-Gene::26054, Gene::3320-Gene::23118, Gene::3146-Gene::55806, Gene::7525-Gene::8751, Disease::DOID:3393-Gene::9314, Disease::DOID:8778-Gene::10285, Disease::DOID:1324-Gene::23532, Disease::DOID:2841-Gene::6701, Disease::DOID:1936-Gene::3569, Disease::DOID:2986-Gene::10550, Disease::DOID:1790-Gene::5734** are predicted only four colour versions.

Table 61. Corrected predicted links obtained by aligning the original synthetic network and four subnetworks with its noisy versions for all the networks.

Network	Nodes	Edges	5% of noise	10% of noise	15% of noise	20% of noise	25% of noise
Synthetic	950	3410	190	360	490	550	600
sb1	170	474	24	44	78	84	112
sb2	250	422	34	49	67	79	123
sb3	330	528	55	63	77	82	104
sb4	200	404	21	45	63	98	132

Table 62. Corrected predicted links obtained by aligning the original Hetionet network and four subnetworks with its noisy versions for all the networks.

Network	Nodes	Edges	5% of noise	10% of noise	15% of noise	20% of noise	25% of noise
Hetionet	37142	6014211	5439	7592	8357	12686	21678
sb1	2095	26567	634	1252	1472	2689	3567
sb2	136	543	12	31	78	87	98
sb3	405	742	22	51	89	112	167
sb4	15056	78234	1278	1310	2014	3244	4543

Running L-HetNetAligner using all the pairs of nodes as input.

To demonstrated the effectiveness of L-HetNaligner we also performed the alignment of synthetic network with its noisy versions by providing, instead of similarity function among the nodes, all pair of nodes in the input step. We selected one synthetic network (previously generated) with 950 nodes and 3410 edges. Then, we built the synthetic versions by random removing 5%, 10%, 15%, 20% and 25% of edges from the original network. Then, we applied L-HetNetAligner to align the synthetic networks with its noisy versions. Then, we applied L-HetNetAligner to align the synthetic networks with its noisy versions. Finally, we computed NCV-GS^3 and F-NC measures for each synthetic network model.

L-HetNetAligner completed the process of alignment of synthetic networks with its noisy counterpart in almost 67 minutes, and the process occupies 8 GB of Memory. Table 63 and Table 66 report the NCV-GS^3 and F-NC scores. The NCV-GS^3 and F-NC values are much lower than NCV-GS^3 and F-NC value obtained when the similarity function among nodes is used in the alignment building.

Impact of the Variation of the Parameters to the Alignment Quality

Table 63. NCV-GS^3 scores obtained by aligning the original synthetic network with its noisy versions for all the networks. In the alignment graph building all pair of nodes were selected.

Network	Altered Networks	NCV-GS^3 on 1 coloured version	NCV-GS^3 on 2 coloured version	NCV-GS^3 on 3 coloured version	NCV-GS^3 on 4 coloured version
N1	0	0.367	0.49	0.59	0.776
	5	0.359	0.476	0.59	0.774
	10	0.355	0.474	0.589	0.768
	15	0.351	0.474	0.586	0.765
	20	0.346	0.47	0.584	0.764
	25	0.335	0.462	0.574	0.755
N2	0	0.364	0.483	0.595	0.783
	5	0.364	0.482	0.593	0.777
	10	0.363	0.48	0.589	0.776
	15	0.358	0.477	0.586	0.776
	20	0.353	0.471	0.582	0.771
	25	0.342	0.457	0.573	0.765
N3	0	0.352	0.488	0.595	0.788
	5	0.349	0.476	0.588	0.785
	10	0.342	0.468	0.581	0.784
	15	0.34	0.464	0.576	0.778
	20	0.336	0.459	0.57	0.77
	25	0.336	0.456	0.57	0.762
N4	0	0.368	0.487	0.593	0.787
	5	0.364	0.487	0.588	0.786
	10	0.363	0.475	0.584	0.779
	15	0.345	0.468	0.583	0.758
	20	0.337	0.462	0.577	0.755
	25	0.335	0.452	0.574	0.752
N5	0	0.371	0.487	0.598	0.786
	5	0.367	0.472	0.593	0.783
	10	0.365	0.47	0.585	0.782
	15	0.345	0.468	0.579	0.766
	20	0.343	0.466	0.579	0.766
	25	0.342	0.461	0.571	0.756
N6	0	0.379	0.48	0.597	0.789
	5	0.377	0.479	0.593	0.784
	10	0.35	0.476	0.593	0.784
	15	0.348	0.473	0.592	0.779
	20	0.347	0.455	0.588	0.771
	25	0.344	0.451	0.579	0.771
N7	0	0.366	0.482	0.598	0.782
	5	0.357	0.481	0.584	0.779
	10	0.355	0.472	0.583	0.779
	15	0.353	0.457	0.575	0.752
	20	0.336	0.457	0.573	0.752
	25	0.334	0.451	0.572	0.752
N8	0	0.377	0.484	0.591	0.787
	5	0.376	0.48	0.591	0.786
	10	0.376	0.467	0.583	0.785
	15	0.371	0.463	0.581	0.757
	20	0.363	0.46	0.579	0.756
	25	0.344	0.451	0.571	0.756
N9	0	0.372	0.48	0.591	0.789
	5	0.365	0.478	0.587	0.782
	10	0.36	0.476	0.583	0.781
	15	0.357	0.474	0.582	0.78
	20	0.349	0.455	0.58	0.761
	25	0.34	0.451	0.579	0.754
N10	0	0.368	0.484	0.598	0.785
	5	0.359	0.475	0.595	0.785
	10	0.354	0.471	0.595	0.78
	15	0.353	0.469	0.577	0.778
	20	0.347	0.462	0.575	0.755
	25	0.338	0.456	0.572	0.753
N11	0	0.374	0.483	0.596	0.788
	5	0.374	0.475	0.588	0.77
	10	0.367	0.474	0.588	0.77
	15	0.365	0.472	0.586	0.768
	20	0.355	0.467	0.573	0.762
	25	0.343	0.464	0.569	0.752
N12	0	0.377	0.489	0.584	0.79
	5	0.363	0.488	0.578	0.783
	10	0.361	0.48	0.578	0.766
	15	0.351	0.471	0.575	0.764
	20	0.344	0.469	0.572	0.761
	25	0.341	0.468	0.572	0.753

Table 64. F-NC scores obtained by aligning the original synthetic network with its noisy versions for all the networks. In the alignment graph building all pair of nodes were selected.

Network	Altered Networks	F-NC on 1 coloured version	F-NC on 2 coloured version	F-NC on 3 coloured version	F-NC on 4 coloured version
N1	0	0.32	0.45	0.568	0.72
	5	0.314	0.444	0.531	0.719
	10	0.309	0.441	0.529	0.711
	15	0.308	0.44	0.507	0.709
	20	0.304	0.435	0.506	0.704
	25	0.304	0.434	0.501	0.703
N2	0	0.32	0.454	0.595	0.713
	5	0.32	0.454	0.592	0.71
	10	0.317	0.438	0.591	0.71
	15	0.311	0.437	0.59	0.71
	20	0.305	0.436	0.566	0.708
	25	0.304	0.434	0.522	0.707
N3	0	0.314	0.452	0.595	0.719
	5	0.311	0.448	0.592	0.716
	10	0.31	0.446	0.572	0.713
	15	0.31	0.445	0.544	0.712
	20	0.303	0.444	0.537	0.711
	25	0.302	0.441	0.533	0.707
N4	0	0.314	0.45	0.556	0.718
	5	0.314	0.444	0.553	0.718
	10	0.308	0.441	0.535	0.713
	15	0.305	0.439	0.523	0.709
	20	0.305	0.437	0.505	0.703
	25	0.302	0.436	0.504	0.703
N5	0	0.319	0.448	0.599	0.715
	5	0.318	0.446	0.58	0.715
	10	0.314	0.441	0.574	0.714
	15	0.311	0.439	0.571	0.713
	20	0.31	0.436	0.569	0.71
	25	0.307	0.435	0.523	0.71
N6	0	0.318	0.448	0.589	0.717
	5	0.318	0.447	0.589	0.717
	10	0.315	0.443	0.588	0.71
	15	0.314	0.443	0.558	0.707
	20	0.308	0.441	0.53	0.707
	25	0.302	0.44	0.522	0.703
N7	0	0.319	0.454	0.556	0.718
	5	0.319	0.452	0.555	0.716
	10	0.317	0.45	0.506	0.714
	15	0.315	0.45	0.506	0.709
	20	0.302	0.445	0.505	0.708
	25	0.301	0.435	0.501	0.703
N8	0	0.32	0.449	0.555	0.72
	5	0.311	0.449	0.545	0.72
	10	0.311	0.441	0.544	0.718
	15	0.309	0.439	0.536	0.716
	20	0.305	0.438	0.514	0.712
	25	0.303	0.434	0.501	0.709
N9	0	0.319	0.451	0.581	0.714
	5	0.318	0.451	0.565	0.714
	10	0.318	0.449	0.565	0.713
	15	0.31	0.445	0.563	0.712
	20	0.305	0.441	0.557	0.71
	25	0.301	0.438	0.555	0.705
N10	0	0.318	0.452	0.591	0.719
	5	0.318	0.444	0.557	0.716
	10	0.315	0.441	0.546	0.716
	15	0.312	0.44	0.538	0.712
	20	0.308	0.436	0.532	0.709
	25	0.307	0.435	0.505	0.706
N11	0	0.316	0.449	0.561	0.719
	5	0.315	0.449	0.559	0.711
	10	0.313	0.445	0.544	0.709
	15	0.312	0.443	0.54	0.705
	20	0.305	0.438	0.535	0.704
	25	0.303	0.436	0.524	0.702
N12	0	0.32	0.447	0.595	0.72
	5	0.318	0.444	0.578	0.717
	10	0.315	0.442	0.56	0.717
	15	0.311	0.442	0.552	0.717
	20	0.31	0.439	0.518	0.713
	25	0.308	0.434	0.514	0.709

Table 65. F-NC scores obtained by aligning the original synthetic network with its noisy versions for all the networks. In the alignment graph building all pair of nodes were selected.

**NCV-GS^3**	**L-HetNetAligner Parameters**
0.957	Homogeneous Match : 1<br>Heterogeneous Match: 0.9<br>Homogeneous Mismatch: 0.5<br>Heterogeneous Mismatch: 0.4<br>Homogeneous Gap: 0.2<br>Heterogeneous Gap: 0.1
0.95	Homogeneous Match : **0.95**<br>Heterogeneous Match: 0.9<br>Homogeneous Mismatch: 0.5<br>Heterogeneous Mismatch: 0.4<br>Homogeneous Gap: 0.2<br>Heterogeneous Gap: 0.1
0.947	Homogeneous Match : 1<br>Heterogeneous Match: **0.8**<br>Homogeneous Mismatch: 0.5<br>Heterogeneous Mismatch: 0.4<br>Homogeneous Gap: 0.2<br>Heterogeneous Gap: 0.1
0.951	Homogeneous Match : 1<br>Heterogeneous Match: 0.9<br>Homogeneous Mismatch: **0.45**<br>Heterogeneous Mismatch: 0.4<br>Homogeneous Gap: 0.2<br>Heterogeneous Gap: 0.1
0.951	Homogeneous Match : 1<br>Heterogeneous Match: 0.9<br>Homogeneous Mismatch: 0.5<br>Heterogeneous Mismatch: **0.3**<br>Homogeneous Gap: 0.2<br>Heterogeneous Gap: 0.1
0.954	Homogeneous Match : 1<br>Heterogeneous Match: 0.9<br>Homogeneous Mismatch: 0.5<br>Heterogeneous Mismatch: 0.4<br>Homogeneous Gap: **0.15**<br>Heterogeneous Gap: 0.1
0.952	Homogeneous Match : 1<br>Heterogeneous Match: 0.9<br>Homogeneous Mismatch: 0.5<br>Heterogeneous Mismatch: 0.4<br>Homogeneous Gap: 0.2<br>Heterogeneous Gap: **0.05**

Table 66. F-NC scores obtained by aligning the original synthetic network with its noisy versions for all the networks. In the alignment graph building all pair of nodes were selected.

F-NC	L-HetNetAligner Parameters
0.817	Homogeneous Match : 1<br>Heterogeneous Match: 0.9<br>Homogeneous Mismatch: 0.5<br>Heterogeneous Mismatch: 0.4<br>Homogeneous Gap: 0.2<br>Heterogeneous Gap: 0.1
0.811	Homogeneous Match : **0.95**<br>Heterogeneous Match: 0.9<br>Homogeneous Mismatch: 0.5<br>Heterogeneous Mismatch: 0.4<br>Homogeneous Gap: 0.2<br>Heterogeneous Gap: 0.1
0.79	Homogeneous Match : 1<br>Heterogeneous Match: **0.813**<br>Homogeneous Mismatch: 0.5<br>Heterogeneous Mismatch: 0.4<br>Homogeneous Gap: 0.2<br>Heterogeneous Gap: 0.1
0.813	Homogeneous Match : 1<br>Heterogeneous Match: 0.9<br>Homogeneous Mismatch: **0.45**<br>Heterogeneous Mismatch: 0.4<br>Homogeneous Gap: 0.2<br>Heterogeneous Gap: 0.1
0.812	Homogeneous Match : 1<br>Heterogeneous Match: 0.9<br>Homogeneous Mismatch: 0.5<br>Heterogeneous Mismatch: **0.3**<br>Homogeneous Gap: 0.2<br>Heterogeneous Gap: 0.1
0.814	Homogeneous Match : 1<br>Heterogeneous Match: 0.9<br>Homogeneous Mismatch: 0.5<br>Heterogeneous Mismatch: 0.4<br>Homogeneous Gap: **0.15**<br>Heterogeneous Gap: 0.1
0.814	Homogeneous Match : 1<br>Heterogeneous Match: 0.9<br>Homogeneous Mismatch: 0.5<br>Heterogeneous Mismatch: 0.4<br>Homogeneous Gap: 0.2<br>Heterogeneous Gap: **0.05**
